# Dysbiosis in the Gut Microbiota in Patients with Inflammatory Bowel Disease during Remission

**DOI:** 10.1128/spectrum.00616-22

**Published:** 2022-05-09

**Authors:** Anthea Pisani, Philipp Rausch, Corinna Bang, Sarah Ellul, Trevor Tabone, Claire Marantidis Cordina, Graziella Zahra, Andre Franke, Pierre Ellul

**Affiliations:** a Department of Medicine, Mater Dei Hospital, Msida, Malta; b Institute of Clinical Molecular Biology, Christian-Albrechts-University of Kiel, Kiel, Germany; c Department of Surgery, Mater Dei Hospital, Msida, Malta; d Department of Pathology, Mater Dei Hospital, Msida, Malta; University of Nebraska-Lincoln

**Keywords:** inflammatory bowel disease, remission, dysbiosis, flavonoid-degrading bacteria, *Enterobacteriaceae*, microbiota

## Abstract

Inflammatory bowel disease (IBD) is a chronic, relapsing, inflammatory disorder which comprises two main conditions: Crohn’s disease (CD) and ulcerative colitis (UC). Although the etiology of IBD has not been fully elucidated, the gut microbiota is hypothesized to play a vital role in its development. The aim of this cross-sectional study was to characterize the fecal microbiota in CD or UC patients in a state of remission to reveal potential factors sustaining residual levels of inflammation and triggering disease relapses. Ninety-eight IBD patients in a state of clinical remission (66 UC, 32 CD) and 97 controls were recruited, and stool samples, as well as detailed patient data, were collected. After DNA extraction, the variable regions V1 and V2 of the 16S rRNA gene were amplified and sequenced. Patients with IBD had a decrease in alpha diversity compared to that of healthy controls, and the beta diversity indices showed dissimilarity between the cohorts. Healthy controls were associated with the beneficial organisms unclassified *Akkermansia* species (*Akkermansia uncl*.), *Oscillibacter uncl*., and *Coprococcus uncl*., while flavonoid-degrading bacteria were associated with IBD. Network analysis identified highly central and influential disease markers and a strongly correlated network module of *Enterobacteriaceae* which was associated with IBD and could act as drivers for residual inflammatory processes sustaining and triggering IBD, even in a state of low disease activity. The microbiota in IBD patients is significantly different from that of healthy controls, even in a state of remission, which implicates the microbiota as an important driver of chronicity in IBD.

**IMPORTANCE** Dysbiosis in inflammatory bowel disease (IBD) has been implicated as a causal or contributory factor to the pathogenesis of the disease. This study, done on patients in remission while accounting for various confounding factors, shows significant community differences and altered community dynamics, even after acute inflammation has subsided. A cluster of *Enterobacteriaceae* was linked with Crohn’s disease, suggesting that this cluster, which contains members known to disrupt colonization resistance and form biofilms, persists during quiescence and can lead to chronic inflammation. Flavonoid-degrading bacteria were also associated with IBD, raising the possibility that modification of dietary flavonoids might induce and maintain remission in IBD.

## INTRODUCTION

The gut microbiota has been implicated to have a major role in health and disease. With an estimate of 4 × 10^13^ microbes (1) in the human intestinal tract comprising thousands of different species of bacteria, the abundant and diverse members of the gut microbiota collectively possess a large number of genes that enable them to have critical functions that contribute to the host’s health. These roles include, among others, fermentation of complex carbohydrates to produce short-chain fatty acids (SCFA) (2), synthesis of amino acids (3), and synthesis of certain vitamins (e.g., biotin and phylloquinone) (4). The microbiome is also involved in the development and function of both the local and systemic immune systems with the uncompromised gut microbiota protecting against pathogen infections via a phenomenon called “colonization resistance” (5–7).

The gut microbiome is continually exposed to external perturbations such as infections, antibiotics, and dietary changes. This ecosystem has the ability to respond to perturbations by reaching a limited number of “stable equilibrium states” that are beneficial to both the host and the microbiota (8). A few of these states, however, can disrupt the symbiosis between the host and the bacterial community. Such unfavorable states are termed “dysbiosis.” Dysbiosis can be defined as a “stable” and detrimental disturbance in the complex biological system of the microbiome. In the structure of the microbiome, this can have long-lasting compositional and functional implications for the microbiota and the host and can be an important factor in the pathogenesis or chronicity of certain diseases (8). One such disease attributed to the microbiota is inflammatory bowel disease (IBD). Ulcerative colitis (UC) and Crohn’s disease (CD), the two main types of IBD, are chronic conditions resulting in relapsing and remitting inflammation of the intestine. Patients with IBD can be either in the active phase of their disease or in remission. Remission is characterized by a decrease or absence of symptoms, together with lack of biochemical, endoscopic, or radiological evidence of inflammation. The recurrent and long-lasting nature of IBD has still not been completely elucidated, with studies postulating that environmental factors, genetics, immunological abnormalities, and particularly the intestinal microbiota are contributory to the onset and course of the disease.

Changes in complexity and composition of communities have been reported before in CD and UC (9–11), which can result in decreased abundances of anaerobic producers of SCFAs and increases in oxidative metabolism, both detrimental to the gut mucosa. In addition, amino acid biosynthesis and carbohydrate metabolism are decreased in favor of nutrient uptake by the microbiota (12). Studies have identified several species or clusters to be differentially abundant between controls and patients with IBD in remission (11, 13–15). Thus, this state of dysbiosis might persist during quiescence, despite the patient not having any clinically significant signs of inflammation, and may contribute to the relapsing and chronic nature of IBD.

We set out to compare the fecal bacterial microbiota in adult patients with a diagnosis of IBD (CD or UC) who are in the state of remission with a cohort of healthy controls. Analyzing patients in the quiescent stage of their disease allows us to assess whether significant and stable community differences and altered community dynamics persist, even after acute inflammation has subsided. This study, therefore, provides insight into potential microbial factors which can have a role in the maintenance of chronicity in IBD or which can act as possible triggers to new flare-ups.

## RESULTS

Patients above the age of 18 years diagnosed with IBD according to the Copenhagen Diagnostic Criteria for either UC or CD who were in remission were recruited from Mater Dei Hospital, Malta. Ninety-eight (*n* = 98) patients were included in the study (Table 1, Fig. S1). The mean age was 46.1 years (standard deviation [SD] of 16.59) and 62.9% were male. In total, we analyzed 66 UC patients (67.3%) and 32 CD patients. With regard to smoking status, 64.3% of patients with IBD were nonsmokers (had never actively smoked cigarettes), 10.2% were smokers (still actively smoking), and 25.5% were ex-smokers (had stopped smoking for at least 6 months). Ninety-seven (*n* = 97) healthy controls were recruited, of which 51.5% were male. The mean age was 44.8 years (SD = 15.88). Gender distribution between the patient cohorts was not significant (*P* = 0.2622; Fisher’s exact test), while CD patients were slightly younger than UC patients (χ^2^ = 7.8428, degrees of freedom [DF] = 2, *P* = 0.01981; Kruskal-Wallis test) and body mass index (BMI) was not different between groups (χ^2^ = 5.7864, DF = 2, *P* > 0.050; Kruskal-Wallis test).

**TABLE 1:** Demographics of patients with UC and CD and of controls

Demographic	UC	CD	Control
Gender			
Male	43 (65.2%)	18 (56.3%)	50 (51.5%)
Female	23 (34.8%)	14 (43.7%)	47 (48.5%)
Age			
<30	11 (16.7%)	13 (40.6%)	30 (30.9%)
31–60	34 (51.5%)	14 (43.8%)	51 (52.6%)
>61	21 (31.8%)	5 (15.6%)	16 (16.5%)
Smoking status			
Nonsmoker	45 (68.2%)	18 (56.3%)	81 (83.5%)
Smoker	4 (6%)	6 (18.8%)	6 (6.2%)
Ex-smoker	17 (25.8%)	8 (25%)	10 (10.3%)
BMI			
18.5–24.9	21 (31.8%)	16 (50%)	41 (42.3%)
25–29.9	25 (37.9%)	11 (34.4%)	37 (38.1%)
>30	20 (30.3%)	5 (15.6%)	19 (19.6%)
UC classification			
E1: ulcerative proctitis	20 (30.3%)		
E2: left sided UC	24 (36.4%)		
E3: pancolitis	22 (33.3%)		
CD classification			
Age at onset			
A1: <16 yrs		6 (18.8%)	
A2: 17–39 yrs		21 (65.6%)	
A3: >40 yrs		5 (15.6%)	
Disease behavior			
B1: inflammatory		23 (71.9%)	
B2/B3: stricturing and/or penetrating		9 (28.1%)	
Disease location			
L1: terminal ileum		11 (34.4%)	
L2: colon		8 (25%)	
L3: ileocolon		13 (40.6%)	
Medication			
5-ASA only	39 (59.1%)	6 (18.8%)	
5-ASA + thiopurine	14 (21.2%)	6 (18.8%)	
5-ASA + thiopurine + biological	8 (12.1%)	8 (25%)	
5-ASA + biological	5 (7.6%)	4 (12.5%)	
Thiopurine only	0	2 (6.3%)	
Thiopurine + biological	0	6 (18.8%)	

### Disease-specific differences in community diversity and composition persist in the absence of active inflammation.

**FIG 1:**
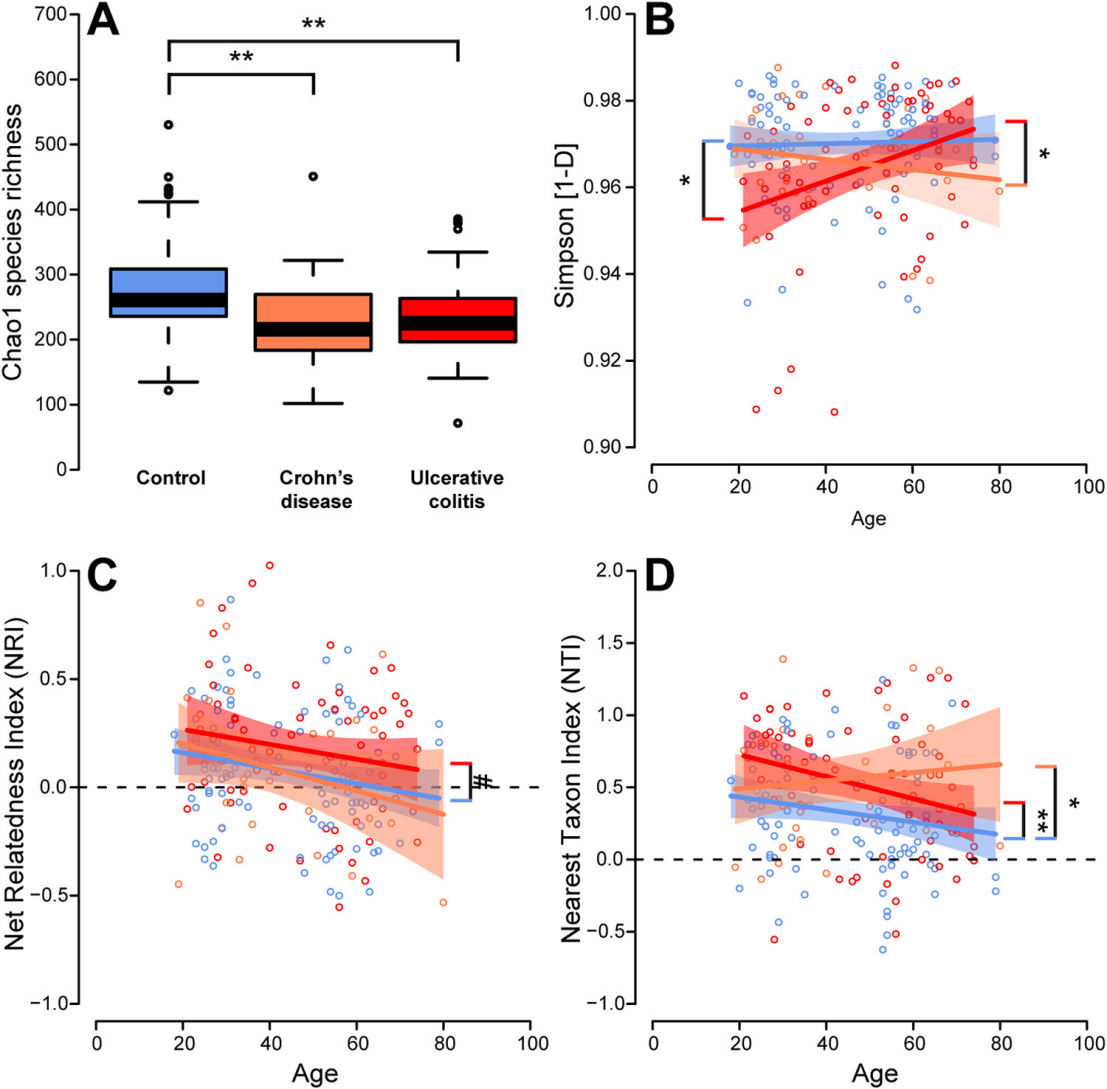
(A) Alpha diversity via the Chao1 species richness in the different cohorts showing a lower alpha diversity in patients with IBD than in controls. (B) Correlation of Simpson diversity with age for the different cohorts. (C and D) Correlation of the phylogenetic measures net relatedness index (NRI) and nearest taxon index (NTI) with age for the different disease cohorts. The dashed line marks the cutoff between phylogenetic overdispersion and phylogenetic clustering (*, *P ≤ *0.050; **, *P ≤ *0.010; ***, *P ≤ *0.001; *#*, *P ≤ *0.1000). Correlation of community diversity and age within the respective patient groups has been assessed for Simpson diversity (*R*^2^_Con._ = 0.00110, *R*^2^_UC_ = 0.09695, *R*^2^_CD_ = 0.0266), NRI (*R*^2^_Con._ = 0.04094, *R*^2^_UC_ = 0.02767, *R*^2^_CD_ = 0.07245), and NTI (*R*^2^_Con._ = 0.03094, *R*^2^_UC_ = 0.07355, *R*^2^_CD_ = 0.01193).

Alpha diversity describes the complexity of communities of a single environment by measuring the number of species (richness), their abundance patterns (evenness), and their phylogenetic complexity. The strongest factor influencing the complexity of the fecal microbial communities was the difference in health condition (healthy control, CD, UC). Patients with IBD displayed reduced average species richness and community evenness compared to those of healthy controls (Fig. 1; Table 2). Correlations of diversity and subject age were observed, which differed with respect to the underlying health condition. Simpson diversity appeared relatively stable across ages for healthy individuals and CD patients, while under UC, communities became more diverse with increasing age (Fig. 1B). This points toward a possible age-dependent decrease of colonization resistance or simply continuing succession of the bacterial communities (16, 17). Phylogenetic measures of alpha diversity include nearest taxon index (NTI) and net relatedness index (NRI), with positive values indicating phylogenetic clustering, values close to zero indicating neutral or random community assembly, and negative values indicating phylogenetic overdispersion, either over the whole phylogenetic tree (NRI) or across the closest related species/tips of the phylogenetic tree (NTI). This study showed an overall decrease in phylogenetic clustering and a trend toward random assembly to phylogenetically overdispersed communities with increasing age. However, on average, phylogenetic clustering could be observed, which was strongest in patients suffering from UC and/or CD compared to the healthy controls, hinting toward either loss of species or replacement by a phylogenetically restricted group of bacteria (Fig. 1C and D; Table 1). Thus, CD and UC patients show on average a less diverse and phylogenetically constrained microbial community compared to that of healthy controls.

**TABLE 2:** Linear model results of the analysis of the different alpha diversity metrics with respect to health condition and age^*a*^

Index	Model factor	DF	*F*	*P*	Comparison	*P* pairwise
Chao	IBD	2,191	8.63883	0.00026	Contr.-CD	0.0041
	IBD				Contr.-UC	0.0016
	IBD				CD-UC	0.8882
Simpson	Age	1,188	2.24911	0.13537	Age:Contr.-CD	0.3515
	IBD	2,188	3.57301	0.02999	Age:Contr.-UC	0.0258
	Age:IBD	2,188	4.00957	0.01971	Age:CD-UC	0.0284
Net relatedness index	Age	1,190	6.19897	0.01364	Contr.-CD	0.9986
	IBD	2,190	2.90330	0.05728	Contr.-UC	0.0566
	IBD				CD-UC	0.2269
Nearest taxon index	Age	1,190	4.70107	0.03139	Contr.-CD	0.0499
	IBD	2,190	5.59379	0.00436	Contr.-UC	0.0094
	IBD				CD-UC	0.9995

a Models were reduced by stepwise model selection, minimizing AIC. DF, degrees of freedom; *F*, *F* value (ANOVA model); *P*, *P* value. Age/BMI was employed as a covariate, and where appropriate, its interaction with IBD pathology was analyzed. “Age:IBD” refers to differences in slope of the age by diversity relationship between the different pathologies. To describe the patterns in more detail, the data were subsetted to describe all potential pairwise differences in slope or average values in the diversity by age relationships. The notation “Age:Contr.-CD” refers to potential differences in slope between control and CD patients, considering its relationship to alpha diversity.

Analyses were also done within the respective disease cohort to investigate the potential differences in diversity between IBD subtypes, following the Montreal classification for CD and UC (18) as well as the influence of medication (e.g., 5-ASA, thiopurine, anti-tumor necrosis factor [TNF]). Although BMI did not have significant effects on alpha diversity in a global analysis and was removed during model selection procedures, it showed minor effects in interaction with different disease subtypes (Table A1). Overall, subtypes had only minor effects on the diversity in CD and UC patients (Table A1), while medication (namely, 5-ASA) in CD patients increased evenness and decreased phylogenetic clustering with age (NRI and NTI). In UC patients (all of whom where on 5-ASA), evenness and richness of the communities increased with increasing age under thiopurine treatment and decreased without it (Table A2). This suggests that, with an advancing age, certain medications can have different effects on the microbiome depending on a person’s age and, thus, may differentially influence inflammatory processes.

**FIG 2:**
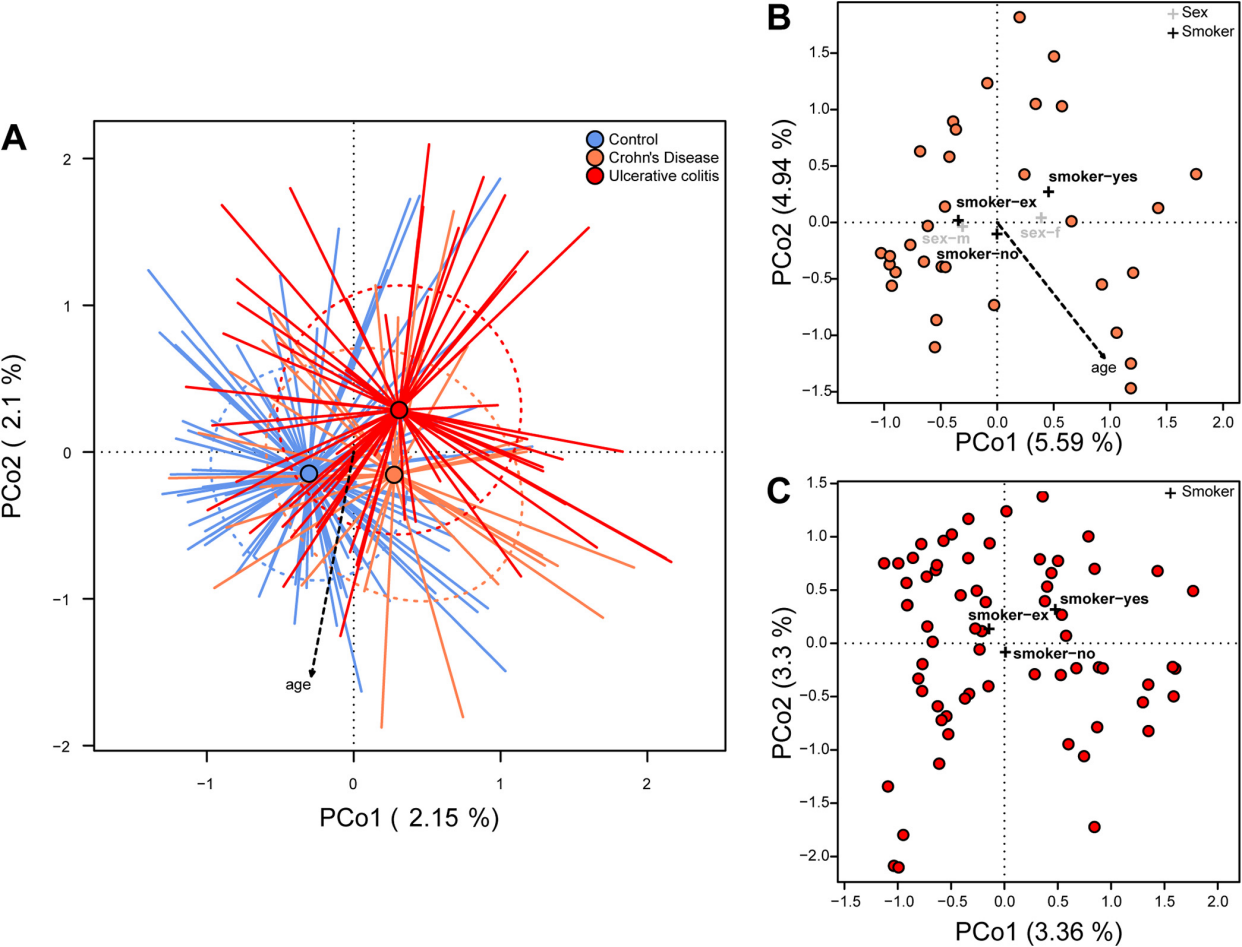
(A) Principal coordinate analysis (PCoA) of Bray-Curtis dissimilarity with respect to IBD status, including the significant correlation of community distance with age (Table 2). The tips of the colored lines each characterize a sample of a specific IBD status (blue, orange, and red representing controls, CD, and UC, respectively). The point where the lines converge, shown as a dot, depicts the center of weight of the respective cohort (centroid). The distance between the centers of weight visualizes the average dissimilarity between the IBD pathologies. Arrows point in the direction of increasing values for that variable, so the lower the PCo1, the greater the age. The amount of variation captured by the respective dimension/axis is shown for the first two axes. (B) PCoA to visualize community differences among only CD patients and (C) UC patients (Appendix Table A3). Crosses indicate the centroid for the nominal significant parameters as detected via permutative ANOVA (PERMANOVA). See Fig. A4 for PCoA of controls.

When analyzing beta diversity, significant differences in community composition and structure on the taxonomic (Bray-Curtis dissimilarity and Jaccard distance) and phylogenetic levels (generalized UniFrac distance) were detected with respect to health condition (control, CD, UC; *P_BC_* = 0.00010, *P_J_* = 0.00010, *P_gUF_* = 0.00010), as well as to age (Fig. 2A; Table 3; Appendix Fig. A2A, A3A, A4). Even after correcting for differences in age, BMI, and gender among individuals, these effects were highly significant. In particular, differences between microbial communities of healthy individuals to either disease were comparably high, while the separation of UC communities was consistently highest in pairwise comparisons (see Table 3). Interestingly, both IBD pathologies differed significantly from each other, although less strongly than they differed from the healthy subjects. Gender significantly influenced beta diversity under Bray-Curtis dissimilarity (*P_BC_* = 0.04162) and Jaccard distance (*P_J_* = 0.01050), while smoking status had no significant effect on overall community composition (Table 3).

**TABLE 3:** PERMANOVA results with respect to gender, smoking status, and IBD using different beta diversity metrics focusing on differential abundance of ASVs (Bray-Curtis dissimilarity), differential occurrence of ASVs (Jaccard distance, see Fig. S2), and phylogenetic differences between communities (generalized UniFrac distance, see Fig. S3)^*a*^

Model or comparison	DF	*F* value	*P* value	adj. *P* value (FDR)	*R* ^2^	adj. *R*^2^
Bray-Curtis dissimilarity						
IBD	2, 192	1.44428	0.00010	0.00054	0.00589	0.00458
Gender	1, 191	1.13670	0.01850	0.04162	0.01098	0.00071
Smoking status	2, 191	1.06048	0.06919	0.12509	0.01490	0.00063
Contr., CD	1, 126	1.31345	0.00010	0.00015	0.01032	0.00246
Contr., UC	1, 160	1.72652	0.00010	0.00015	0.01068	0.00449
CD, UC	1, 96	1.15183	0.01710	0.01710	0.01186	0.00156
Jaccard distance						
IBD	2, 192	1.25200	0.00010	0.00045	0.00564	0.00260
Gender	1, 191	1.08846	0.00350	0.01050	0.01028	0.00046
Smoking status	2, 191	0.99202	0.66913	0.70993	0.01294	−0.00008
Contr., CD	1, 126	1.19526	0.00010	0.00015	0.00940	0.00154
Contr., UC	1, 160	1.42725	0.00010	0.00015	0.00884	0.00265
CD, UC	1, 96	1.03809	0.05249	0.05249	0.01070	0.00039
Generalized UniFrac distance						
IBD	2, 192	2.07221	0.00010	0.00067	0.00629	0.01099
Gender	1, 191	1.21542	0.06159	0.10985	0.01099	0.00111
Smoking status	2, 191	1.06161	0.21498	0.27640	0.02124	0.00064
Contr., CD	1, 126	1.82581	0.00010	0.00015	0.01428	0.00646
Contr., UC	1, 160	2.77997	0.00010	0.00015	0.01708	0.01093
CD, UC	1, 96	1.22807	0.05329	0.05329	0.01263	0.00235

a Contr, control.

Further analyses assessing the influence of various factors on beta diversity among patients with CD and UC showed that age also affected community composition in both CD (*P_BC_* = 0.00639, *P_gUF_* = 0.00350) and UC (*P_J_* = 0.00260) (Table A3). Among individuals suffering from CD, gender was also a factor influencing community composition (*P_J_* = 0.00710). The location of inflammation (subtype L as per Montreal classification) left only weak traces in the taxonomic (Jaccard) and phylogenetic composition of the microbial communities, while age of disease onset (subtype A) significantly influenced the phylogenetic makeup of the communities in CD (Fig. 1B; Fig. A2B, A3B; Table A3). Among UC patients, gender, most medications, and disease subtype (E) did not significantly influence the microbiota composition, while age and smoking exerted some effect, although the associations were no longer significant after correction for multiple testing (Fig. 2C; Fig. A2C, A3C; Table A3).

### Differential abundance and indicator taxa.

**FIG 3:**
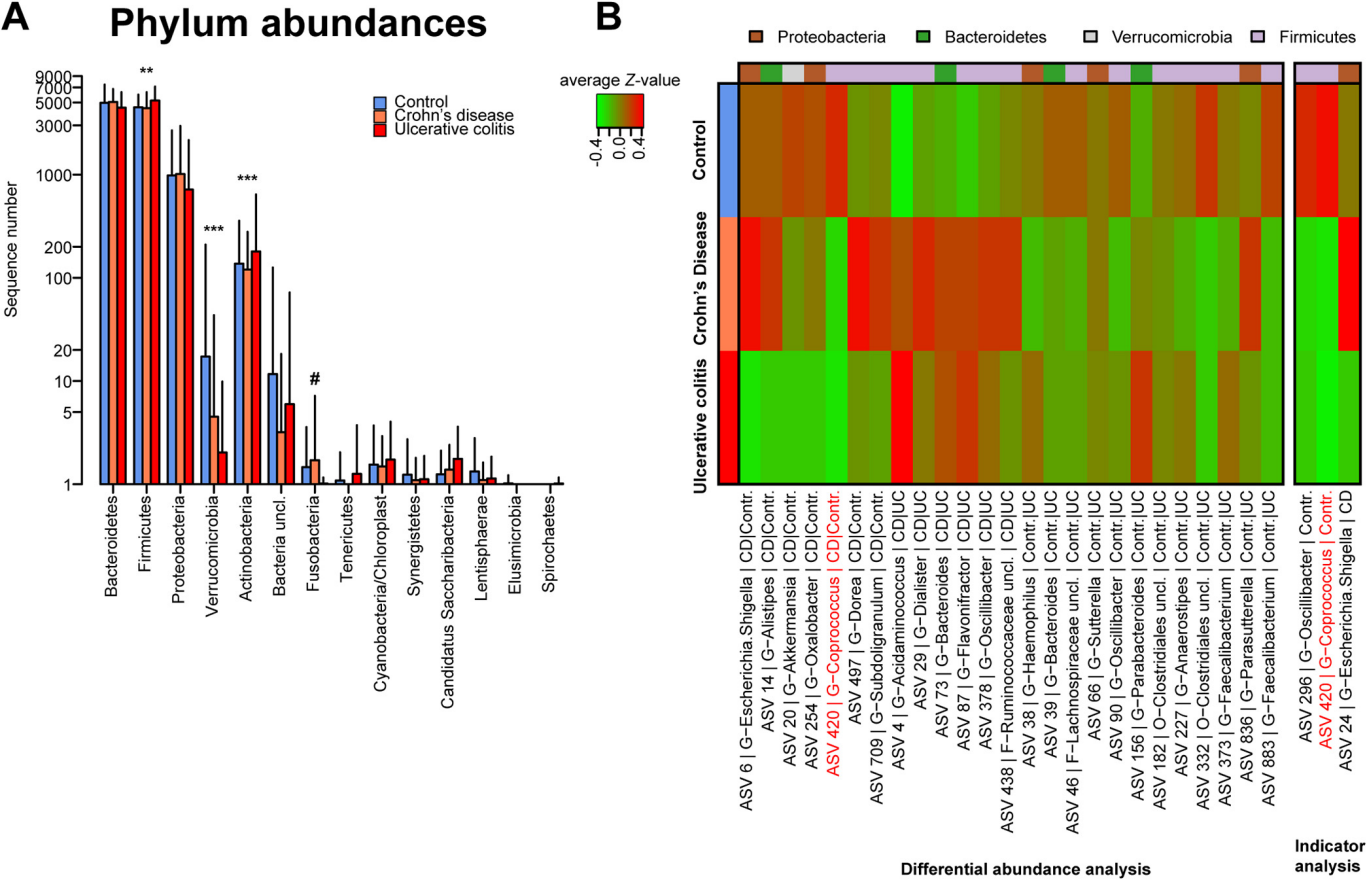
(A) Phylum abundances with respect to IBD status/health condition analyzed via DESeq2. *Firmicutes* (*P*_FDR_ = 0.00264) and *Actinobacteria* (*P*_FDR_ = 0.00005) are more abundant in UC patients, while *Fusobacteria* are increased in CD patients (*P*_FDR_ = 0.05931) and *Verrucomicrobia* are more abundant in healthy controls (*P*_FDR_ = 7.42962 × 10^−11^). (B) Heatmap showing significantly differentially abundant and indicative bacteria (Appendix Tables A5 and A6). *, *P ≤ *0.050; **, *P ≤ *0.010; ***, *P ≤ *0.001; *#*, *P ≤ *0.1000.

Differential abundance analysis was performed to detect differences in taxonomic groups between the different health conditions, correcting for potential influences of age, BMI, and gender. At the phylum level, abundances differed significantly with respect to health condition. *Firmicutes* and *Actinobacteria* were identified as being more abundant in UC patients (false-discovery rate *P* value [*P*_FDR_] of 0.00264 and 0.00005, respectively), while *Fusobacteria* was slightly increased among CD patients (*P*_FDR_ = 0.05931). Only the phylum *Verrucomicrobia* was significantly more abundant in healthy controls (*P*_FDR_ = 7.42962 × 10^−11^) (Fig. 3A; Table A4). Several of these patterns were also represented at the species/amplicon sequence variant (ASV) level. An ASV is a representative, theoretically true sequence derived from error-corrected next-generation sequencing (NGS) amplicon marker experiments, representing a group of bacteria carrying this sequence (19). Twenty-five ASVs that were differentially abundant between the different health conditions were identified (Fig. 3B; Table A5); 13 were overabundant in healthy individuals (e.g., ASV 883 *Faecalibacterium uncl.* and ASV 20 Akkermansia muciniphila), while 6 were the least abundant among the controls. Three ASVs showed higher abundances among CD patients (ASV 6 *Escherichia/Shigella uncl*., ASV 497 *Dorea uncl.*, and ASV-709 *Subdoligranulum uncl.*), while 3 other ASVs had higher abundances in UC patients (ASV 156-*Parabacteroides uncl.*, ASV 373-*Faecalibacterium uncl.*, and ASV 836-*Parasutturella uncl.*) than in patients with other health conditions. Indicator species analysis, done as a complementary analysis to determine characteristic bacteria associated with the different health conditions, showed that healthy controls were characterized through significant associations with two butyrate producers: ASV 296 *Oscillibacter uncl*. (*P = *0.00040, *P_FDR_* < 0.1000) and the also differentially abundant ASV 420 *Coprococcus uncl*. (*P = *0.00050, *P_FDR_* < 0.1000; Fig. 3A; Table A6). Butyrate is vital in maintaining homeostasis within the intestinal epithelium, and a decrease in butyrate-producing bacteria has been linked with IBD (20). Another member of the *Escherichia/Shigella* cluster (ASV 24) was also detected to be significantly predictive of CD (*P* = 0.00090, *P_FDR_* < 0.1000) (Table A6).

Differential abundance analyses with respect to disease subtypes as per Montreal classification (18) were also performed. Within the disease cohorts (CD and UC), a total of 181 ASVs were found to significantly differ in abundance depending on age of onset (A), disease behavior (B), or disease location (L, E) (Table A7; Fig. A5 to A8). Interestingly, several members of the *Proteobacteria* showed a higher abundance in patients with stricturing phenotype (B2 as per Montreal classification) and/or penetrating disease (B3) in CD *Escherichia/Shigella uncl.* (ASVs 13, 31, 282, and 422), *Klebisella uncl.* (ASVs 75 and 101) and *Enterobacter uncl.* (ASV 219; Table A7, Fig. A6). Previous studies have documented a relative increase in bacterial species belonging to the phylum *Proteobacteria*, such as *Enterobacteriaceae*, in patients with IBD (12, 21–23).

Not only did smoking affect beta diversity as described above but associations were also identified with different bacteria, like ASV 227 *Anaerostipes uncl.*, which was more abundant in healthy subjects as well as in nonsmoking individuals, or ASV 373 *Faecalibacterium uncl.*, which showed increased abundance in UC patients and people who actively smoked (Table A8). Stratified by health condition, the influence of three major treatment options, 5-ASA, thiopurine, and infliximab/adalimumab (anti-TNF), on the abundance and occurrence of bacteria was investigated (Table A9). Several bacteria appeared to be influenced in similar ways by the same treatment in both UC and CD, including ASV 1278 Veillonella parvula and ASV 14 *Alistipes uncl.*, which were abundant under the influence of infliximab, or ASV 13-*Escherichia/Shigella uncl.* and ASV 8-*Dialister uncl*., which were more abundant under thiopurine treatment. Conversely, some bacteria were influenced in opposite directions by the same treatment, e.g., Collinsella aerofaciens (ASV 60). In CD patients, this ASV was associated with thiopurine and anti-TNF but not 5-ASA. In UC (where all patients were treated with 5-ASA), this bacterium was associated with individuals without thiopurine or anti-TNF (Table A9).

### Correlation network analysis.

**FIG 4:**
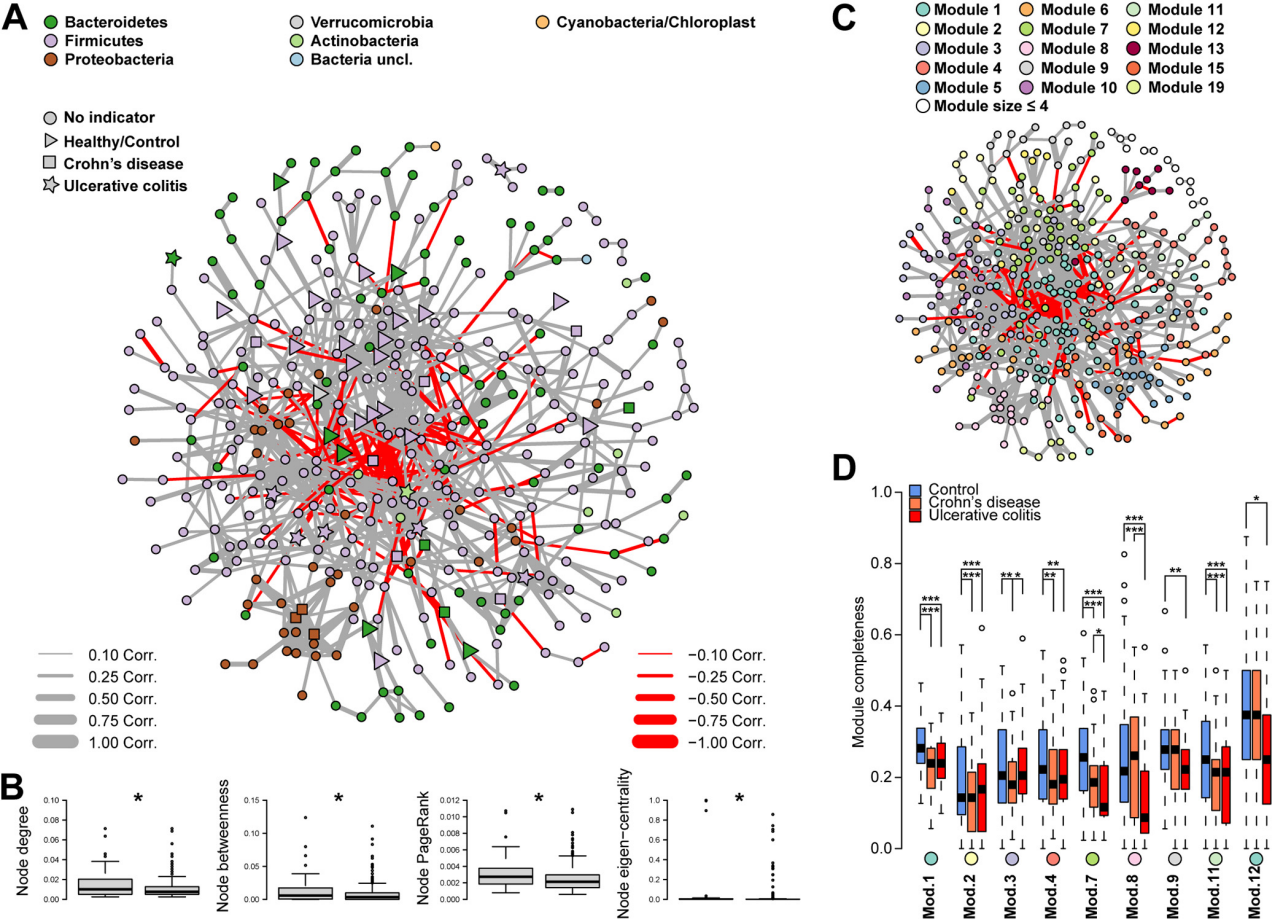
(A) Species level ASV correlation network based on SparCC. (B) Comparison of different average node centralities (degree, betweenness, PageRank, and eigenvector centrality) between IBD indicators and nonindicators tested via permutative Wilcoxon test (*P*_FDR_ ≤ 0.05). (C) Community modules as detected via the fast-greedy algorithm and (D) the association of these community modules to disease are demonstrated by showing the average completeness of the respective module in the respective cohort (*, *P ≤ *0.050; **, *P ≤ *0.010; ***, *P ≤ *0.001; *#*, *P ≤ *0.1000; Appendix Tables A10 and A11).

To investigate the bacterial community as a system of interacting agents whose interactions may change as a cause or consequence of IBD, an ASV coabundance network was constructed and analyzed (Fig. 4). Different bacterial network characteristics were measured to approximate the structural importance of the single bacterial taxa within the network, where the degree of a node refers to the number of connections (number of correlated bacteria) and betweenness measured its importance in connecting other nodes (being in the shortest path between nodes). Eigenvector centrality and PageRank index give importance to ASVs that relate with other important ASVs, with eigenvector centrality emphasizing highly connected groups (24–26). On average, network members which were indicators for IBD or differentially abundant among health conditions showed an increased centrality/importance within the correlation networks (Fig. 4B), suggesting that they play more important roles than other bacteria in the community.

Directly testing the observed importance of single network members showed several indicators and differentially abundant species to be more important than expected by chance. These included ASV 87 Flavonifractor plautii (formerly known as Clostridium orbiscindens or Eubacterium plautii [27, 28]), a flavonoid-degrading bacteria which was associated with IBD patients more than with controls. Flavonifractor plautii showed a high network importance on several levels, with a very central position (betweenness) and many associations with other community members (node degree), which were themselves important mediators (PageRank). Intriguingly, this bacterium was identified before as being more abundant among CD patients than among controls (Table A10). Eggerthella lenta (ASV 648), another flavonoid-degrading bacterium, also appeared to be highly important in the network and associated with patients suffering from UC (Fig. A9; Table A10). Flavonoids, which are plant metabolites, are known to have multiple beneficial properties which include anti-inflammatory effects (29). In addition, several bacteria which occupied highly central positions in the community network were associated with controls, including *Oscillibacter uncl.* (ASV 296 and ASV 90), Alistipes shahii (ASV 16), and members of the order *Clostridiales* (ASV 321 and ASV 749).

Through the analysis of compositional differences of modules between health conditions, closely interacting bacterial groups, which structurally changed due to disease, were identified (Table A11). Four modules (including module 8) were influenced by IBD, which themselves also included several indicator taxa for the different disease conditions. A strongly correlated group of *Proteobacteria* ASVs was identified by having a very high eigenvector centrality and forming a clearly defined network module (module 8; fast-greedy algorithm [30], Table A11; Fig. 4C). This module was characterized by almost exclusive interactions among *Enterobacteriaceae* genera (*Klebsiella*, *Escherichia/Shigella*, *Enterobacter*, *Citrobacter*; 18/23) and some less dominant taxonomic groups. This particular network module further displayed a significantly low completeness in UC (Table A11; Fig. 4D), and its completeness was positively associated with SCCAI (simple clinical colitis activity index) (31) in UC patients and negatively associated with CDAI (Crohn’s disease activity index) (32) in CD patients (Table A11; Fig. A10), adding more evidence that this cluster of *Enterobacteriaceae* may play a role in the pathogenesis of IBD (33). Module completeness refers to how “complete” or “whole” a group of apparently associated bacteria is, as derived from the correlation network (i.e., module). The analyses are based on evaluating how complete the membership of these bacterial groups (ranging from 0, no group members present, to 1, all group members present) is within each individual subject. The resulting measure was then subjected to further analyses with regard to its association to patient characteristics (e.g., IBD condition, age). The *Enterobacteriaceae* have previously been linked to intestinal inflammation via production of lipopolysaccharide that can trigger TLR4 signaling (34). Thus, depending on the type of disease, this bacterial cluster may be able to influence disease severity. Other network modules showed patterns of reduced completeness in the cases of pathology and were also enriched in bacteria associated with changes in health condition (Table A12). This translated to specific subgroups within the microbiome which either drive changes in relation to inflammation or are particularly sensitive to a changing gut environment due to inflammation.

## DISCUSSION

### Alpha and beta diversity differ between patients with IBD in remission and healthy controls.

In this cohort of Maltese IBD patients in remission, significant differences were observed compared to healthy controls. A decrease in alpha diversity in IBD patients is in accordance with other studies (9, 35, 36). This reduction in diversity suggests that the microbiota has less functional capability and redundancy, making it more susceptible to detrimental effects of external perturbations (8). While alpha diversity, as measured by the Simpson index, was relatively stable with increasing age in the controls and CD patients, it increased with age in patients with UC. This points toward a possible age-dependent decrease of colonization resistance or simply continuing succession of the bacterial communities (16, 17). Significant differences were notable in the community composition (beta diversity) between the different cohorts, showing that the communities varied between disease conditions and among disease subtypes even in the absence of active inflammation. Age also influenced beta diversity, suggesting changes in composition with time due to natural progression of communities with host age as observed elsewhere (37). So far, most studies have been conducted with individuals in a state of active disease, which therefore does not reflect the condition of quiescence found between flare-ups as was demonstrated in this study.

### Differential abundance analysis shows community differences in an inactive disease state.

Certain ASVs demonstrated higher abundances among IBD patients, like. *Dorea uncl.* (ASV 497) and *Subdoligranulum uncl.* (ASV-709), which were more abundant in CD patients. Studies have reported a decrease in these species in patients with IBD; however, either these patients were newly diagnosed and thus in a state of active disease or no information was available on the disease activity of these patients (12, 38). In UC, the *Parabacteroides uncl.* (ASV 156) was more abundant than in the other health conditions, as shown by Rajilić-Stojanović (11). *Parasutturella uncl.* (ASV 836), a member of the *Proteobacteria*, was also noted to be more abundant in UC, and this tallies with another study (39). Members of *Verrucomicrobia*, including Akkermansia muciniphila (ASV 20), were more abundant in controls. A. muciniphila degrades mucin, producing acetate and propionate (40). Levels of *A. muciniphilia* are decreased in obesity and metabolic disorders (41), while administration of this bacterium to mice resulted in partial protection against diet-induced obesity and was beneficial to the gut mucosal barrier (42). *Akkermansia* spp. have been documented to be decreased in IBD patients in remission (11) and also in active disease (43, 44). In mouse studies, *A. muciniphila* improved dextran sulfate sodium-induced UC (45), though another study showed an increase in the levels of *A. muciniphila* in mice with dextran sulfate sodium-induced colitis (46). The decreased abundance in our patients with IBD compared to that in our healthy controls supports the notion that *A. muciniphilia* or its components could be used as a therapeutic agent (41).

The observed differences in bacterial abundances show that although patients are in the quiescent phase of their disease (lack of symptoms, normal fecal calprotectin, normal endoscopy, normal imaging), a high degree of disease-related community differences remain. This pattern is well reflected in the significant differences in community composition between the different disease cohorts in this study. A study assessing whether the presence of certain microbes is associated with a greater risk of relapse when anti-TNF treatment is stopped found that lower levels of *Bacteroides* spp., Faecalibacterium prausnitzii, and Clostridium coccoides result in a higher risk of relapse (47). This suggests that further assessment of the microbiome during remission can influence treatment decisions.

### Flavonoid-degrading bacteria have a central role in a correlation network.

Two flavonoid-degrading bacteria, Flavonifractor plautii (ASV 87) and Eggerthella lenta (ASV 648), showed a high and consistent network importance on several levels and are also strongly associated with CD and UC, respectively. Flavonoids are secondary plant metabolites with antioxidant properties, which have been shown to suppress inflammation in both *in vivo* and *in vitro* studies, decreasing the severity of various inflammatory diseases, including IBD (29, 48, 49). Furthermore, many flavonoids have been described to possess antibacterial, antifungal, and even antiviral properties (50). Flavonoids have been studied as a potential therapeutic option for IBD through various mechanisms, including preservation of the epithelial barrier, various immunomodulatory functions influencing the gut microbiota, and modulation of enterohormone secretion (51). Though not commonly recognized, Flavonifractor plautii was shown to be more abundant in controls than in IBD patients in remission (11) or cases of colorectal cancer (52). Thus, the role of flavonoid-degrading bacteria in IBD remains to be elucidated, but one can hypothesize that the increased breakdown or modification of dietary flavonoids might add to chronicity of IBD by reducing the antibacterial and anti-inflammatory effects of these dietary compounds (29, 50, 52).

### *Enterobacteriaceae* cluster is linked to CD.

Differential abundance analyses revealed that patients with the stricturing and/or penetrating subtype of CD (higher severity) had a higher abundance of *Escherichia/Shigella uncl.* (ASVs 13, 31, 282, and 422), *Klebsiella uncl.* (ASVs 75 and 101), and *Enterobacter uncl.* (ASV 219) than patients with nonstricturing, nonpenetrating phenotype. In addition, ASV 6 *Escherichia/Shigella* was significantly reduced among healthy individuals. Network analyses revealed a strongly correlated group of *Proteobacteria* ASVs (*Klebsiella*, *Escherichia/Shigella*, *Enterobacter*, *Citrobacter*). Some of the ASVs in this group were strong indicators of CD and also showed repeatedly high network importance in other measures. Various studies have linked *Enterobacteriaceae* with inflammatory processes. Taxa in the *Enterobacteriaceae* have been shown to disrupt colonization resistance and form biofilms (7), which might be reflected in the tightly connected (strongly coabundant) module 8. Biofilm formation may be beneficial during inflammation and ecological disturbance and increases persistence in the gut environment (53), albeit at the expense of potential competitive abilities (54). Adherent-invasive Escherichia coli has been isolated from ileal mucosa of patients with CD and has been shown to disrupt the gut mucosal barrier by attachment to the epithelial cells or mucosal tissue invasion (33). *Klebsiella* spp. have been previously associated with IBD, especially in active CD (36), possibly by a TH1 cell induction capability (55) as evidenced by research on the microbiota, immunological investigations, and mouse studies (56). *Klebsiella* spp. have also been associated with primary sclerosing cholangitis (57), which in turn is associated with IBD (mainly UC). Citrobacter has been documented to trigger inflammation by induction of Th17 cells (6) and was also found to elicit colitis in mice (58). The persistence of the *Enterobacteriaceae* can therefore be part of the explanation for the chronicity of IBD, as the completeness of this particular bacterial group also correlates with measures of severity.

In conclusion, a high degree of dysbiosis remains in IBD patients in remission, further supporting the notion of long-lasting dysbiotic patterns supporting and maintaining inflammation and chronicity in IBD. The persistent decrease in alpha diversity and clear differences in community composition in patients with IBD may leave these individuals in a state more vulnerable to external and internal perturbations despite being in remission. In view of the association of *Enterobacteriaceae* and flavonoid-degrading bacteria to IBD, one could hypothesize that these bacteria, some of which are known pathobionts, can flourish during a perturbation, resulting in a relapse of active inflammation. Future studies might indicate whether abundance of certain pathobionts is associated with a shorter time to relapse and might influence decisions pertaining to management of IBD.

## MATERIALS AND METHODS

### Patient recruitment.

Ethical approval was obtained from the University of Malta Research Ethics Committee (Ref 32/2017) and from Mater Dei Hospital. Maltese patients above the age of 18 years diagnosed with IBD according to the Copenhagen Diagnostic Criteria for either UC (59) or CD (60) were recruited from the Gastroenterology Outpatients department at Mater Dei Hospital, Malta between September 2018 and September 2019. Patients had to be in the quiescent phase of the disease. Quiescence was defined as a fecal calprotectin (FCP) value below 250 μg/g (61) and/or normal recent (<6 months) endoscopic examination and/or normal recent (<6 months) small bowel imaging. All FCP tests were carried out at the Bioscientia Institute for Medical Diagnostics, while all endoscopies and small bowel imaging were carried out at Mater Dei Hospital.

Exclusion criteria were (i) usage of antibiotics, probiotics, bowel-cleansing medications, or systemic corticosteroids within the previous 3 months, (ii) acute illness, chronic autoimmune or inflammatory medical conditions, a history of malignancy, previous gastrointestinal surgery, or current other active gastrointestinal disease, and (iii) females who were pregnant or lactating.

Control samples were randomly collected from a healthy cohort. Controls had the same inclusion and exclusion criteria, with the exception that they were not known to suffer from IBD.

### DNA extraction.

The microbiota analysis was carried out at the Institute of Clinical Molecular Biology in Kiel, Germany. DNA was extracted and purified using the QIAamp fast DNA stool minikit automated on the QIAcube (Qiagen, Hilden, Germany) as described by Trautmann et al. (62).

### Bacterial 16S rRNA gene sequencing.

Variable regions V1 and V2 of the 16S rRNA gene were amplified using the primer pair 27F-338R in a dual-barcoding approach according to Caporaso et al. (63). DNA was diluted 1:10 prior to PCR, and PCR products were verified using the electrophoresis in agarose gel and normalized using the SequalPrep normalization plate kit (Thermo Fischer Scientific, Waltham, MA, USA). Subsequently, PCR products were pooled equimolarly and sequenced on the Illumina MiSeq v3 2 by 300 bp (Illumina Inc., San Diego, CA, USA). Demultiplexing after sequencing was based on 0 mismatches in the barcode sequences *bcl2fastq*.

### Quality control, classification, and binning of sequences.

Data processing was performed using the DADA2 version 1.10 (64) workflow for big data sets resulting in abundance tables of amplicon sequence variants (ASVs). All sequencing runs were handled separately for error correction, read merging, and combined chimera detection. ASVs underwent taxonomic annotation using the naive Bayesian classifier implemented in DADA2 using the RDP database (v16) as a taxonomic reference and, if possible, classified sequences at the species level (65, 66). ASV sequences were aligned via NAST-alignment to the SILVA core database and filtered for informative sites (constant gaps, constant bases) in *mothur* (67–70). Phylogenetic tree construction on ASV alignment generated was carried out using *FastTree 2.1* using the CAT substitution model with Γ-correction and improved accuracy, employing more minimum evolution rounds for initial tree search (-spr 4), more exhaustive tree search (-mlacc 2), and a slower initial tree search (-slownni) (71).

### Statistical methods for microbiome analyses.

Microbiome data were rarefied to 11,800 reads/sample to ensure comparable and sufficient coverage across samples (average Good’s coverage of 99.87% ± 0.001 SD). Species diversity indices were calculated in *vegan* for *R* 3.5.1 (72), analyzed via multivariate linear models, and simplified via backwards selection. Phylogenetic measures of alpha diversity (NTI and NRI) were derived using the *picante* package, based on 999 permutations against a null model preserving relative species richness within the communities (73, 74). Multivariate analyses of alpha diversity measures were carried out with linear models using BMI and age as covariates in interaction with IBD and using model selection to derive the most optimal statistical model (minimizing Akaike information criterium [AIC] criterion, likelihood ratio tests) in *R* v3.5.3. For beta diversity analyses, taxon-based beta diversity metrics were calculated in the *vegan* package in *R* v3.5.3 (72, 75, 76). As measure of phylogenetic differentiation between bacterial communities, the generalized UniFrac measure (d^0.5^) was utilized (77, 78). Distances were ordinated via principal coordinate analysis (PCoA) avoiding negative eigenvalues by square root transformation. Testing for community separation between the different health conditions or other patient characteristics was performed by distance-based (conditional) redundancy analyses and permutative analysis of variance (PERMANOVA; 10,000 permutations) (79–81) including evaluation of fit via *R*^2^ and adjusted *R*^2^ (82). The fit of centroids and/or biplots of continuous variables was assessed via a goodness-of-fit test (10,000 permutations), based on the results of the PERMANOVAs.

Indicator species analysis was based on 10,000 permutations utilizing the generalized indicator value (*IndVal.g*) as implemented in the *indicspecies* package for *R*. Differential abundances were tested via general linear models (GLMs) using a negative binomial error structure as implemented in *DESeq2* using bacteria present in at least 5% of samples (83), correcting for the covariates’ genders and ages in the analysis (model: bacteria ~ gender + age + IBD + error), and correcting for the covariates gender and age in the analysis (model: bacteria ~ gender + age + IBD). *P* values of differential abundance analyses and indicator analyses were adjusted via the Benjamini-Hochberg procedure (84).

To generate coabundance networks, the SparCC algorithm (85) as implemented in *mothur* was employed (50 samplings, 100 iterations, 10,000 permutations, *P* value adjustment via Benjamini-Hochberg procedure) based on ASV abundances (shared among 10% samples) at a *P_FDR_* value cutoff of ≤0.05 and a correlation strength above the median of nonzero correlations. Network modules were detected by the fast-greedy algorithm (30), and node-based importance/centrality measures weighing different node characteristics were calculated as implemented in *igraph* 1.2.4.1 on the weighted networks (|R| values). (24–26). To assess whether bacteria are more important than expected by chance, bacterial vertices were permuted 10,000 times and their centrality within the random network was calculated. These individual statistics of random communities served as a baseline random distribution to compare the observed centralities against via a one-sided *Z*-test to determine significance. Module differentiation between health conditions was tested via redundancy analysis of Hellinger transformed community subsets based on module membership, comparable to so-called “*eigengene analysis*” (86). Binomial GLMs were employed to assess relative module completeness with respect to health condition and the indices CDAI (32) and SCCAI (31).

### Data availability.

Raw sequence data and relevant metadata can be accessed online at the European Nucleotide Archive (https://www.ebi.ac.uk/ena/) under the accession number PRJEB44440 for CD and UC cases, as well as under PRJEB47162 for the healthy controls.

## References

[1] Sender R, Fuchs S, Milo R. 2016. Are we really vastly outnumbered? Revisiting the ratio of bacterial to host cells in humans. Cell 164:337–340. doi:10.1016/j.cell.2016.01.013. 26824647

[2] Flint HJ, Scott KP, Duncan SH, Louis P, Forano E. 2012. Microbial degradation of complex carbohydrates in the gut. Gut Microbes 3:289–306. doi:10.4161/gmic.19897. 22572875 PMC3463488

[3] Metges CC. 2000. Contribution of microbial amino acids to amino acid homeostasis of the host. J Nutr 130:1857S–1864S. doi:10.1093/jn/130.7.1857S. 10867063

[4] Hooper LV, Midtvedt T, Gordon JI. 2002. How host-microbial interactions shape the nutrient environment of the mammalian intestine. Annu Rev Nutr 22:283–307. doi:10.1146/annurev.nutr.22.011602.092259. 12055347

[5] Bouskra D, Brézillon C, Bérard M, Werts C, Varona R, Boneca IG, Eberl G. 2008. Lymphoid tissue genesis induced by commensals through NOD1 regulates intestinal homeostasis. Nature 456:507–510. doi:10.1038/nature07450. 18987631

[6] Atarashi K, Tanoue T, Ando M, Kamada N, Nagano Y, Narushima S, Suda W, Imaoka A, Setoyama H, Nagamori T, Ishikawa E, Shima T, Hara T, Kado S, Jinnohara T, Ohno H, Kondo T, Toyooka K, Watanabe E, Yokoyama SI, Tokoro S, Mori H, Noguchi Y, Morita H, Ivanov II, Sugiyama T, Nuñez G, Camp JG, Hattori M, Umesaki Y, Honda K. 2015. Th17 cell induction by adhesion of microbes to intestinal epithelial cells. Cell 163:367–380. doi:10.1016/j.cell.2015.08.058. 26411289 PMC4765954

[7] Stecher B, Berry D, Loy A. 2013. Colonization resistance and microbial ecophysiology: using gnotobiotic mouse models and single-cell technology to explore the intestinal jungle. FEMS Microbiol Rev 37:793–829. doi:10.1111/1574-6976.12024. 23662775

[8] Sommer F, Anderson JM, Bharti R, Raes J, Rosenstiel P. 2017. The resilience of the intestinal microbiota influences health and disease. Nat Rev Microbiol 15:630–638. doi:10.1038/nrmicro.2017.58. 28626231

[9] Manichanh C, Rigottier-Gois L, Bonnaud E, Gloux K, Pelletier E, Frangeul L, Nalin R, Jarrin C, Chardon P, Marteau P, Roca J, Dore J. 2006. Reduced diversity of faecal microbiota in Crohn’s disease revealed by a metagenomic approach. Gut 55:205–211. doi:10.1136/gut.2005.073817. 16188921 PMC1856500

[10] Martinez C, Antolin M, Santos J, Torrejon A, Casellas F, Borruel N, Guarner F, Malagelada JR. 2008. Unstable composition of the fecal microbiota in ulcerative colitis during clinical remission. Am J Gastroenterol 103:643–648. doi:10.1111/j.1572-0241.2007.01592.x. 18341488

[11] Rajilić-Stojanović M, Shanahan F, Guarner F, De Vos WM. 2013. Phylogenetic analysis of dysbiosis in ulcerative colitis during remission. Inflamm Bowel Dis 19:481–488. doi:10.1097/MIB.0b013e31827fec6d. 23385241

[12] Morgan XC, Tickle TL, Sokol H, Gevers D, Devaney KL, Ward DV, Reyes JA, Shah SA, LeLeiko N, Snapper SB, Bousvaros A, Korzenik J, Sands BE, Xavier RJ, Huttenhower C. 2012. Dysfunction of the intestinal microbiome in inflammatory bowel disease and treatment. Genome Biol 13:R79. doi:10.1186/gb-2012-13-9-r79. 23013615 PMC3506950

[13] Wang W, Chen L, Zhou R, Wang X, Song L, Huang S, Wang G, Xia B. 2014. Increased proportions of Bifidobacterium and the Lactobacillus group and loss of butyrate-producing bacteria in inflammatory bowel disease. J Clin Microbiol 52:398–406. doi:10.1128/JCM.01500-13. 24478468 PMC3911339

[14] Sha S, Xu B, Wang X, Zhang Y, Wang H, Kong X, Zhu H, Wu K. 2013. The biodiversity and composition of the dominant fecal microbiota in patients with inflammatory bowel disease. Diagn Microbiol Infect Dis 75:245–251. doi:10.1016/j.diagmicrobio.2012.11.022. 23276768

[15] Machiels K, Joossens M, Sabino J, De Preter V, Arijs I, Eeckhaut V, Ballet V, Claes K, Van Immerseel F, Verbeke K, Ferrante M, Verhaegen J, Rutgeerts P, Vermeire S. 2014. A decrease of the butyrate-producing species Roseburia hominis and Faecalibacterium prausnitzii defines dysbiosis in patients with ulcerative colitis. Gut 63:1275–1283. doi:10.1136/gutjnl-2013-304833. 24021287

[16] Ghosh TS, Das M, Jeffery IB, O'Toole PW. 2020. Adjusting for age improves identification of gut microbiome alterations in multiple diseases. Elife 9. doi:10.7554/eLife.50240. PMC7065848 32159510

[17] Zhang X, Zhong H, Li Y, Shi Z, Zhang Z, Zhou X, Ren H, Tang S, Han X, Lin Y, Wang D, Yang F, Fang C, Fu Z, Wang L, Zhu S, Hou Y, Xu X, Yang H, Wang J, Kristiansen K, Li J, Ji L. 2019. Age-dependent sexual dimorphism in the adult human gut microbiota. bioRxiv. 10.1101/646620.

[18] Silverberg MS, Satsangi J, Ahmad T, Arnott IDR, Bernstein CN, Brant SR, Caprilli R, Colombel JF, Gasche C, Geboes K, Jewell DP, Karban A, Loftus EV, Peña AS, Riddell RH, Sachar DB, Schreiber S, Steinhart AH, Targan SR, Vermeire S, Warren BF. 2005. Toward an integrated clinical, molecular and serological classification of inflammatory bowel disease: report of a Working Party of the 2005 Montreal World Congress of Gastroenterology. Can J Gastroenterol 19 (Suppl A):5A–36A. doi:10.1155/2005/269076. 16151544

[19] Eren AM, Maignien L, Sul WJ, Murphy LG, Grim SL, Morrison HG, Sogin ML. 2013. Oligotyping: differentiating between closely related microbial taxa using 16S rRNA gene data. Methods Ecol Evol 4:1111–1119. doi:10.1111/2041-210X.12114. 24358444 PMC3864673

[20] Venegas DP, De La Fuente MK, Landskron G, González MJ, Quera R, Dijkstra G, Harmsen HJM, Faber KN, Hermoso MA. 2019. Short chain fatty acids (SCFAs)mediated gut epithelial and immune regulation and its relevance for inflammatory bowel diseases. Front Immunol 10:277. doi:10.3389/fimmu.2019.00277. 30915065 PMC6421268

[21] Seksik P, Rigottier-Gois L, Gramet G, Sutren M, Pochart P, Marteau P, Jian R, Doré J. 2003. Alterations of the dominant faecal bacterial groups in patients with Crohn’s disease of the colon. Gut 52:237–242. doi:10.1136/gut.52.2.237. 12524406 PMC1774977

[22] Kostic AD, Xavier RJ, Gevers D. 2014. The microbiome in inflammatory bowel disease: current status and the future ahead. Gastroenterology 146:1489–1499. doi:10.1053/j.gastro.2014.02.009. 24560869 PMC4034132

[23] Frank DN, Robertson CE, Hamm CM, Kpadeh Z, Zhang T, Chen H, Zhu W, Sartor RB, Boedeker EC, Harpaz N, Pace NR, Li E. 2011. Disease phenotype and genotype are associated with shifts in intestinal-associated microbiota in inflammatory bowel diseases. Inflamm Bowel Dis 17:179–184. doi:10.1002/ibd.21339. 20839241 PMC3834564

[24] Brin S, Page L. 1998. The anatomy of a large-scale hypertextual Web search engine. Comput Netw ISDN Syst 30:107–117. doi:10.1016/S0169-7552(98)00110-X.

[25] Freeman LC. 1978. Centrality in social networks conceptual clarification. Soc Networks 1:215–239. doi:10.1016/0378-8733(78)90021-7.

[26] Csardi G, Nepusz T. 2006. The igraph software package for complex network research. InterJournal Complex Syst:1695.

[27] Carlier JP, Bedora-Faure M, K’ouas G, Alauzet C, Mory F. 2010. Proposal to unify Clostridium orbiscindens Winter et al. 1991 and Eubacterium plautii (Séguin 1928) Hofstad and Aasjord 1982, with description of Flavonifractor plautii gen. nov., comb. nov., and reassignment of Bacteroides capillosus to Pseudoflavonifrac. Int J Syst Evol Microbiol 60:585–590. doi:10.1099/ijs.0.016725-0. 19654357

[28] Winter J, Popoff MR, Grimont P, Bokkenheuser VD. 1991. Clostridium orbiscindens sp. nov., a human intestinal bacterium capable of cleaving the flavonoid C-ring. Int J Syst Bacteriol 41:355–357. doi:10.1099/00207713-41-3-355. 1883711

[29] Yi YS. 2018. Regulatory roles of flavonoids on inflammasome activation during inflammatory responses. Mol Nutr Food Res 62:1800147. doi:10.1002/mnfr.201800147. 29774640

[30] Clauset A, Newman MEJ, Moore C. 2004. Finding community structure in very large networks. Phys Rev E 70:066111. doi:10.1103/PhysRevE.70.066111. 15697438

[31] Walmsley RS, Ayres RCSS, Pounder RE, Allan RN. 1998. A simple clinical colitis activity index. Gut 43:29–32. doi:10.1136/gut.43.1.29. 9771402 PMC1727189

[32] Best WR, Becktel JM, Singleton JW, Kern F. 1976. Development of a Crohn’s disease activity index: national cooperative Crohn’s disease study. Gastroenterology 70:439–444. doi:10.1016/S0016-5085(76)80163-1. 1248701

[33] Darfeuille-Michaud A, Boudeau J, Bulois P, Neut C, Glasser AL, Barnich N, Bringer MA, Swidsinski A, Beaugerie L, Colombel JF. 2004. High prevalence of adherent-invasive Escherichia coli associated with ileal mucosa in Crohn’s disease. Gastroenterology 127:412–421. doi:10.1053/j.gastro.2004.04.061. 15300573

[34] Poltorak A, He X, Smirnova I, Liu MY, Van Huffel C, Du X, Birdwell D, Alejos E, Silva M, Galanos C, Freudenberg M, Ricciardi-Castagnoli P, Layton B, Beutler B. 1998. Defective LPS signaling in C3H/HeJ and C57BL/10ScCr mice: mutations in Tlr4 gene. Science 282:2085–2088. doi:10.1126/science.282.5396.2085. 9851930

[35] Lloyd-Price J, Arze C, Ananthakrishnan AN, Schirmer M, Avila-Pacheco J, Poon TW, Andrews E, Ajami NJ, Bonham KS, Brislawn CJ, Casero D, Courtney H, Gonzalez A, Graeber TG, Hall AB, Lake K, Landers CJ, Mallick H, Plichta DR, Prasad M, Rahnavard G, Sauk J, Shungin D, Vázquez-Baeza Y, White RA, Bishai J, Bullock K, Deik A, Dennis C, Kaplan JL, Khalili H, McIver LJ, Moran CJ, Nguyen L, Pierce KA, Schwager R, Sirota-Madi A, Stevens BW, Tan W, ten Hoeve JJ, Weingart G, Wilson RG, Yajnik V, Braun J, Denson LA, Jansson JK, Knight R, Kugathasan S, McGovern DPB, Petrosino JF, Stappenbeck TS, Winter HS, IBDMDB Investigators, et al. 2019. Multi-omics of the gut microbial ecosystem in inflammatory bowel diseases. Nature 569:655–662. doi:10.1038/s41586-019-1237-9. 31142855 PMC6650278

[36] Ott SJ, Musfeldt M, Wenderoth DF, Hampe J, Brant O, Fölsch UR, Timmis KN, Schreiber S. 2004. Reduction in diversity of the colonic mucosa associated bacterial microflora in patients with active inflammatory bowel disease. Gut 53:685–693. doi:10.1136/gut.2003.025403. 15082587 PMC1774050

[37] Yatsunenko T, Rey FE, Manary MJ, Trehan I, Dominguez-Bello MG, Contreras M, Magris M, Hidalgo G, Baldassano RN, Anokhin AP, Heath AC, Warner B, Reeder J, Kuczynski J, Caporaso JG, Lozupone CA, Lauber C, Clemente JC, Knights D, Knight R, Gordon JI. 2012. Human gut microbiome viewed across age and geography. Nature 486:222–227. doi:10.1038/nature11053. 22699611 PMC3376388

[38] Gevers D, Kugathasan S, Denson LA, Vázquez-Baeza Y, Van Treuren W, Ren B, Schwager E, Knights D, Song SJ, Yassour M, Morgan XC, Kostic AD, Luo C, González A, McDonald D, Haberman Y, Walters T, Baker S, Rosh J, Stephens M, Heyman M, Markowitz J, Baldassano R, Griffiths A, Sylvester F, Mack D, Kim S, Crandall W, Hyams J, Huttenhower C, Knight R, Xavier RJ. 2014. The treatment-naive microbiome in new-onset Crohn’s disease. Cell Host Microbe 15:382–392. doi:10.1016/j.chom.2014.02.005. 24629344 PMC4059512

[39] Ricanek P, Lothe SM, Frye SA, Rydning A, Vatn MH, Tønjum T. 2012. Gut bacterial profile in patients newly diagnosed with treatment-naïve Crohn’s disease. Clin Exp Gastroenterol 5:173–186. doi:10.2147/CEG.S33858. 23049264 PMC3459595

[40] Derrien M, Vaughan EE, Plugge CM, de Vos WM. 2004. Akkermansia municiphila gen. nov., sp. nov., a human intestinal mucin-degrading bacterium. Int J Syst Evol Microbiol 54:1469–1476. doi:10.1099/ijs.0.02873-0. 15388697

[41] Cani PD, de Vos WM. 2017. Next-generation beneficial microbes: the Case of Akkermansia muciniphila. Front Microbiol 8:1765. doi:10.3389/fmicb.2017.01765. 29018410 PMC5614963

[42] Everard A, Belzer C, Geurts L, Ouwerkerk JP, Druart C, Bindels LB, Guiot Y, Derrien M, Muccioli GG, Delzenne NM, De Vos WM, Cani PD. 2013. Cross-talk between Akkermansia muciniphila and intestinal epithelium controls diet-induced obesity. Proc Natl Acad Sci USA 110:9066–9071. doi:10.1073/pnas.1219451110. 23671105 PMC3670398

[43] Png CW, Lindén SK, Gilshenan KS, Zoetendal EG, McSweeney CS, Sly LI, McGuckin MA, Florin THJ. 2010. Mucolytic bacteria with increased prevalence in IBD mucosa augment in vitro utilization of mucin by other bacteria. Am J Gastroenterol 105:2420–2428. doi:10.1038/ajg.2010.281. 20648002

[44] Lo Presti A, Del Chierico F, Altomare A, Zorzi F, Cella E, Putignani L, Luca Guarino MP, Monteleone G, Cicala M, Angeletti S, Ciccozzi M. 2019. Exploring the genetic diversity of the 16S rRNA gene of Akkermansia muciniphila in IBD and IBS. Future Microbiol 14:1497–1509. doi:10.2217/fmb-2019-0175. 31850811

[45] Zhai R, Xue X, Zhang L, Yang X, Zhao L, Zhang C. 2019. Strain-specific anti-inflammatory properties of two Akkermansia muciniphila strains on chronic colitis in mice. Front Cell Infect Microbiol 9. doi:10.3389/fcimb.2019.00239. PMC6624636 31334133

[46] Berry D, Schwab C, Milinovich G, Reichert J, Mahfoudh KB, Decker T, Engel M, Hai B, Hainzl E, Heider S, Kenner L, Müller M, Rauch I, Strobl B, Wagner M, Schleper C, Urich T, Loy A. 2012. Phylotype-level 16S rRNA analysis reveals new bacterial indicators of health state in acute murine colitis. ISME J 6:2091–2106. doi:10.1038/ismej.2012.39. 22572638 PMC3475367

[47] Rajca S, Grondin V, Louis E, Vernier-Massouille G, Grimaud JC, Bouhnik Y, Laharie D, Dupas JL, Pillant H, Picon L, Veyrac M, Flamant M, Savoye G, Jian R, Devos M, Paintaud G, Piver E, Allez M, Mary JY, Sokol H, Colombel JF, Seksik P. 2014. Alterations in the intestinal microbiome (Dysbiosis) as a predictor of relapse after infliximab withdrawal in Crohn’s disease. Inflamm Bowel Dis 20:978–986. doi:10.1097/MIB.0000000000000036. 24788220

[48] Braune A, Blaut M. 2016. Bacterial species involved in the conversion of dietary flavonoids in the human gut. Gut Microbes 7:216–234. doi:10.1080/19490976.2016.1158395. 26963713 PMC4939924

[49] Pérez-Cano FJ, Massot-Cladera M, Rodríguez-Lagunas MJ, Castell M. 2014. Flavonoids affect host-microbiota crosstalk through TLR modulation. Antioxidants 3:649–670. doi:10.3390/antiox3040649. 26785232 PMC4665504

[50] Cushnie TPT, Lamb AJ. 2005. Antimicrobial activity of flavonoids. Int J Antimicrob Agents 26:343–356. doi:10.1016/j.ijantimicag.2005.09.002. 16323269 PMC7127073

[51] Li M, Weigmann B. 2022. A novel pathway of flavonoids protecting against inflammatory bowel disease: modulating enteroendocrine system. Metabolites 12:31. doi:10.3390/metabo12010031. 35050153 PMC8777795

[52] Gupta A, Dhakan DB, Maji A, Saxena R, Pk VP, Mahajan S, Pulikkan J, Kurian J, Gomez AM, Scaria J, Amato KR, Sharma AK, Sharma VK. 2019. Association of Flavonifractor plautii, a flavonoid-degrading bacterium, with the gut microbiome of colorectal cancer patients in India. mSystems 4. doi:10.1128/mSystems.00438-19. PMC7407896 31719139

[53] Martinez-Medina M, Naves P, Blanco J, Aldeguer X, Blanco JE, Blanco M, Ponte C, Soriano F, Darfeuille-Michaud A, Garcia-Gil LJ. 2009. Biofilm formation as a novel phenotypic feature of adherent-invasive Escherichia coli (AIEC). BMC Microbiol 9:202. doi:10.1186/1471-2180-9-202. 19772580 PMC2759958

[54] Kamada N, Kim YG, Sham HP, Vallance BA, Puente JL, Martens EC, Núñez G. 2012. Regulated virulence controls the ability of a pathogen to compete with the gut microbiota. Science 336:1325–1329. doi:10.1126/science.1222195. 22582016 PMC3439148

[55] Atarashi K, Suda W, Luo C, Kawaguchi T, Motoo I, Narushima S, Kiguchi Y, Yasuma K, Watanabe E, Tanoue T, Thaiss CA, Sato M, Toyooka K, Said HS, Yamagami H, Rice SA, Gevers D, Johnson RC, Segre JA, Chen K, Kolls JK, Elinav E, Morita H, Xavier RJ, Hattori M, Honda K. 2017. Ectopic colonization of oral bacteria in the intestine drives TH1 cell induction and inflammation. Science 358:359–365. doi:10.1126/science.aan4526. 29051379 PMC5682622

[56] Rashid T, Ebringer A, Wilson C. 2013. The role of Klebsiella in Crohn’s disease with a potential for the use of antimicrobial measures. Int J Rheumatol 2013:610393. doi:10.1155/2013/610393. 24223596 PMC3810322

[57] Nakamoto N, Sasaki N, Aoki R, Miyamoto K, Suda W, Teratani T, Suzuki T, Koda Y, Chu PS, Taniki N, Yamaguchi A, Kanamori M, Kamada N, Hattori M, Ashida H, Sakamoto M, Atarashi K, Narushima S, Yoshimura A, Honda K, Sato T, Kanai T. 2019. Gut pathobionts underlie intestinal barrier dysfunction and liver T helper 17 cell immune response in primary sclerosing cholangitis. Nat Microbiol 4:492–503. doi:10.1038/s41564-018-0333-1. 30643240

[58] Mullineaux-Sanders C, Collins JW, Ruano-Gallego D, Levy M, Pevsner-Fischer M, Glegola-Madejska IT, Sågfors AM, Wong JLC, Elinav E, Crepin VF, Frankel G. 2017. Citrobacter rodentium relies on commensals for colonization of the colonic mucosa. Cell Rep 21:3381–3389. doi:10.1016/j.celrep.2017.11.086. 29262319 PMC5746604

[59] Langholz E. 1999. Ulcerative colitis. An epidemiological study based on a regional inception cohort, with special reference to disease course and prognosis. Dan Med Bull 46:400–415. 10605619

[60] Munkholm P. 1997. Crohn’s disease-occurrence, course and prognosis. An epidemiological cohort-study. Ugeskr Laeger 159:2400–2401.

[61] D’Haens G, Ferrante M, Vermeire S, Baert F, Noman M, Moortgat L, Geens P, Iwens D, Aerden I, Van Assche G, Van Olmen G, Rutgeerts P. 2012. Fecal calprotectin is a surrogate marker for endoscopic lesions in inflammatory bowel disease. Inflamm Bowel Dis 18:2218–2224. doi:10.1002/ibd.22917. 22344983

[62] Trautmann T, Bang C, Franke A, Vincent D, Reinshagen K, Boettcher M. 2020. The impact of oral sodium chloride supplementation on thrive and the intestinal microbiome in neonates with small bowel ostomies: a prospective cohort study. Front Immunol 11:1421. doi:10.3389/fimmu.2020.01421. 32754153 PMC7365880

[63] Caporaso JG, Lauber CL, Walters WA, Berg-Lyons D, Huntley J, Fierer N, Owens SM, Betley J, Fraser L, Bauer M, Gormley N, Gilbert JA, Smith G, Knight R. 2012. Ultra-high-throughput microbial community analysis on the Illumina HiSeq and MiSeq platforms. ISME J 6:1621–1624. doi:10.1038/ismej.2012.8. 22402401 PMC3400413

[64] Callahan BJ, McMurdie PJ, Rosen MJ, Han AW, Johnson AJA, Holmes SP. 2016. DADA2: high-resolution sample inference from Illumina amplicon data. Nat Methods 13:581–583. doi:10.1038/nmeth.3869. 27214047 PMC4927377

[65] Cole JR, Wang Q, Fish JA, Chai B, McGarrell DM, Sun Y, Brown CT, Porras-Alfaro A, Kuske CR, Tiedje JM. 2014. Ribosomal Database Project: data and tools for high throughput rRNA analysis. Nucleic Acids Res 42:D633–D642. doi:10.1093/nar/gkt1244. 24288368 PMC3965039

[66] Wang Q, Garrity GM, Tiedje JM, Cole JR. 2007. Naïve Bayesian classifier for rapid assignment of rRNA sequences into the new bacterial taxonomy. Appl Environ Microbiol 73:5261–5267. doi:10.1128/AEM.00062-07. 17586664 PMC1950982

[67] Schloss PD. 2009. A high-throughput DNA sequence aligner for microbial ecology studies. PLoS One 4:e8230. doi:10.1371/journal.pone.0008230. 20011594 PMC2788221

[68] Schloss PD, Westcott SL, Ryabin T, Hall JR, Hartmann M, Hollister EB, Lesniewski RA, Oakley BB, Parks DH, Robinson CJ, Sahl JW, Stres B, Thallinger GG, Van Horn DJ, Weber CF. 2009. Introducing mothur: open-source, platform-independent, community-supported software for describing and comparing microbial communities. Appl Environ Microbiol 75:7537–7541. doi:10.1128/AEM.01541-09. 19801464 PMC2786419

[69] Pruesse E, Quast C, Knittel K, Fuchs BM, Ludwig W, Rg Peplies J, Glö CF. 2007. SILVA: a comprehensive online resource for quality checked and aligned ribosomal RNA sequence data compatible with ARB. Nucleic Acids Res 35:7188–7196. doi:10.1093/nar/gkm864. 17947321 PMC2175337

[70] Quast C, Pruesse E, Yilmaz P, Gerken J, Schweer T, Yarza P, Peplies J, Glöckner FO. 2013. The SILVA ribosomal RNA gene database project: improved data processing and web-based tools. Nucleic Acids Res 41:D590–D596. doi:10.1093/nar/gks1219. 23193283 PMC3531112

[71] Price MN, Dehal PS, Arkin AP. 2010. FastTree 2 - Approximately maximum-likelihood trees for large alignments. PLoS One 5:e9490. doi:10.1371/journal.pone.0009490. 20224823 PMC2835736

[72] Oksanen J, Guillaume BF, Kindt R. 2011. CRAN - Package vegan. https://CRAN.R-project.org/package=vegan.

[73] Gotelli NJ. 2000. Null model analysis of species co-occurrence patterns. Ecology 81:2606–2621. doi:10.1890/0012-9658(2000)081[2606:NMAOSC]2.0.CO;2.

[74] Kembel SW, Cowan PD, Helmus MR, Cornwell WK, Morlon H, Ackerly DD, Blomberg SP, Webb CO. 2010. Picante: r tools for integrating phylogenies and ecology. Bioinformatics 26:1463–1464. doi:10.1093/bioinformatics/btq166. 20395285

[75] Bray JR, Curtis JT. 1957. An ordination of the upland forest communities of Southern Wisconsin. Ecol Monogr 27:325–349. doi:10.2307/1942268.

[76] Jaccard P. 1901. Étude comparative de la distribution florale dans une portion des Alpes et des Jura. Bull la Société Vaudoise Des Sci Nat 37:547–579.

[77] Chen J, Bittinger K, Charlson ES, Hoffmann C, Lewis J, Wu GD, Collman RG, Bushman FD, Li H, Bateman A. 2012. Associating microbiome composition with environmental covariates using generalized UniFrac distances. Bioinformatics 28:2106–2113. doi:10.1093/bioinformatics/bts342. 22711789 PMC3413390

[78] Chen J. 2018. GUniFrac: generalized UniFrac distances. https://cran.r-project.org/web/packages/GUniFrac/GUniFrac.pdf. 10.1093/bioinformatics/bts342 PMC3413390 22711789

[79] Legendre P, Legendre L. 1998. Numerical ecology. 2nd English edition. Developments in environmental modelling. 20:1–853. Elsevier, Philadelphia, PA.

[80] Legendre P, Anderson MJ. 1999. Distance-based redundancy analysis: testing multispecies responses in multifactorial ecological experiments. Ecol Monogr 69:1–24. doi:10.1890/0012-9615(1999)069[0001:DBRATM]2.0.CO;2.

[81] Legendre P, Gallagher ED. 2001. Ecologically meaningful transformations for ordination of species data. Oecologia 129:271–280. doi:10.1007/s004420100716. 28547606

[82] Blanchet FG, Legendre P, Borcard D. 2008. Forward selection of explanatory variables. Ecology 89:2623–2632. doi:10.1890/07-0986.1. 18831183

[83] Love MI, Huber W, Anders S. 2014. Moderated estimation of fold change and dispersion for RNA-seq data with DESeq2. Genome Biol 15:550. doi:10.1186/s13059-014-0550-8. 25516281 PMC4302049

[84] Benjamini Y, Hochberg Y. 1995. Controlling the false discovery rate: a practical and powerful approach to multiple testing. J R Stat Soc Ser B 57:289–300. doi:10.1111/j.2517-6161.1995.tb02031.x.

[85] Friedman J, Alm EJ. 2012. Inferring correlation networks from genomic survey data. PLoS Comput Biol 8:e1002687. doi:10.1371/journal.pcbi.1002687. 23028285 PMC3447976

[86] Langfelder P, Horvath S. 2007. Eigengene networks for studying the relationships between co-expression modules. BMC Syst Biol 1:54. doi:10.1186/1752-0509-1-54. 18031580 PMC2267703

