# Supplementary material for: Dysbiosis in the Gut Microbiota in Patients with Inflammatory Bowel Disease during Remission
Source: Microbiol Spectr. 2022 May 9;10(3):e00616-22. doi: 10.1128/spectrum.00616-22 (PMC9241752; doi:10.1128/spectrum.00616-22)
Supplement: SUPPLEMENTAL FILE 1 — Supplemental material. Download spectrum.00616-22-s001.pdf, PDF file, 3.2 MB [file spectrum.00616-22-s001.pdf]

Appendix Tables and Figures:

	Diversity Index	Factor	DF	F value	P value
CD	Chao Index	BMI	1,30	1.79358	0.19055
	Simpson Index	Sub-A (Age of onset)	2,29	2.62785	0.08936
		Age	1,26	0.89156	0.35375
		Sub-B (Behaviour)	2,26	0.72176	0.49538
		Age:subB	2,26	2.56687	0.09608
	Net Relatedness Index	Sub-A (Age of onset)	2,29	4.16775	0.02564
		Age	1,28	2.62486	0.11641
		Sub-B (Behaviour)	2,28	2.80364	0.07765
		Sub-L (Location)	2,29	2.42440	0.10627
	Nearest Taxon Index	Sub-L (Location)	2,26	0.31998	0.72898
		BMI	1,26	0.76718	0.38911
		Sub-L (Location:BMI)	2,26	2.57193	0.09568
UC	Chao Index	Age	1,59	4.78449	0.03269
		Sub-E (Extent)	2,59	0.78579	0.46047
		BMI	1,59	0.63341	0.42930
		Sub-E (Extent):BMI	2,59	3.41319	0.03957
	Simpson Index	Age	1,59	7.76078	0.00717
		Sub-E (Extent)	2,59	2.31232	0.10794
		BMI	1,59	2.87050	0.09549
		Sub-E (Extent):BMI	2,59	2.89559	0.06316
	Nearest Taxon Index	Age	1,64	5.08071	0.02763

Appendix Table 1: Linear model results of the analysis of the different alpha diversity metrics with respect to the phenotype according to the Montreal Classification and BMI.

	Diversity index	Factor	DF	F value	P value
CD	Chao Index	Other medication	1,30	3.72959	0.06295
	Simpson Index	Age	1,28	0.91626	0.34665
		5-ASA	1,28	0.21315	0.64787
		Age:5-ASA	1,28	5.26633	0.02945
	Nearest Taxon Index	Age	1,28	0.47106	0.49815
		5-ASA	1,28	2.36664	0.13518
		Age:5-ASA	1,28	8.63595	0.00653
		Age	1,28	0.37638	0.54450
		Thiopurine	1,28	0.18712	0.66864
		Age:Thiopurine	1,28	2.97681	0.09549
	Net Relatedness Index	Age	1,28	3.03390	0.09252
		5-ASA	1,28	4.28040	0.04790
		Age:5-ASA	1,28	6.56397	0.01608
		Age	1,28	3.02086	0.09319
		Thiopurine	1,28	2.18367	0.15064
		Age:Thiopurine	1,28	8.49376	0.00693
UC	Chao Index	Age	1,61	4.87295	0.03105
		Thiopurine	1,61	2.14566	0.14811
		BMI	1,61	0.66450	0.41815
		Thiopurine: BMI	1,61	5.47906	0.02253
		Age	1,64	4.50098	0.03775
		Other medication	1,64	4.91756	0.03014
	Simpson Index	Age	1,61	7.85147	0.00680
		Thiopurine	1,61	3.85577	0.05414
		BMI	1,61	2.82403	0.09798
		Thiopurine: BMI	1,61	5.45123	0.02286
		Age	1,64	6.87115	0.01093
	Nearest Taxon Index	Age	1,64	5.08071	0.02763

Appendix Table 2: Linear model results of the analysis of the different alpha diversity metrics with respect to Age and Medication

	Distance	Factor	Sub-category	DF	F	*P* value	P_{FDR}	adj.R2
CD	Bray-Curtis	Anthropometric	Gender	1,30	1.11759	0.0477	0.21193	0.00378
			Smoking	2,29	1.08228	0.0458	0.21193	0.00528
		Medication	5-ASA	1,30	0.9755	0.61444	0.61934	-0.00079
			Thiopurine	1,30	0.99417	0.49725	0.56627	-0.00019
			Anti-TNF	1,30	1.01784	0.35756	0.56627	0.00058
			Other medication	1,30	1.09665	0.07439	0.23278	0.00311
		Subtypes	Age of onset (Sub-A)	2,29	1.02521	0.26887	0.56627	0.00162
			Behaviour (Sub-B)	2,29	1.0006	0.47555	0.56627	0.00004
			Location (Sub-L)	2,29	1.00443	0.42796	0.56627	0.00029
CD	Jaccard	Anthropometric	Gender	1,30	1.06139	0.0071	0.06344	0.00198
			Smoking	2,29	0.99867	0.50075	0.58179	-0.00009
		Medication	5-ASA	1,30	1.05515	0.0163	0.06344	0.00178
			Thiopurine	1,30	1.0217	0.16288	0.36964	0.00070
			Anti-TNF	1,30	0.99785	0.51045	0.58179	-0.00007
			Other medication	1,30	0.99167	0.62424	0.62214	-0.00027
		Subtypes	Age of onset (Sub-A)	2,29	0.9983	0.51715	0.58179	-0.00011
			Behaviour (Sub-B)	2,29	0.9993	0.48775	0.58179	-0.00005
			Location (Sub-L)	2,29	1.02224	0.08389	0.25167	0.00143
CD	Generalized UniFrac	Anthropometric	Gender	1,30	1.01522	0.40476	0.53173	0.00049
			Smoking	2,29	0.99537	0.47685	0.53173	-0.00030
		Medication	5-ASA	1,30	0.96159	0.57744	0.57584	-0.00124
			Thiopurine	1,30	0.99378	0.47175	0.53173	-0.00020
			Anti-TNF	1,30	1.03141	0.35026	0.53173	0.00101
			Other medication	1,30	1.05846	0.27367	0.53173	0.00188
		Subtypes	Age of onset (Sub-A)	2,29	1.16977	0.0413	0.29967	0.01083
			Behaviour (Sub-B)	2,29	1.05461	0.24818	0.53173	0.00351
			Location (Sub-L)	2,29	1.13881	0.06659	0.29967	0.00888
UC	Bray-Curtis	Anthropometric	Gender	1,64	1.03569	0.24608	0.47095	0.00055
			Smoking	2,63	1.09118	0.0228	0.13799	0.00280
		Medication	Thiopurine	1,64	0.97137	0.67043	0.66793	-0.00044
			Anti-TNF	1,64	0.99442	0.49925	0.66793	-0.00009
			Other medication	1,64	1.06269	0.13589	0.43286	0.00096
		Subtypes	Extent (Sub-E)	2,63	0.99021	0.56224	0.66793	-0.00030
UC	Jaccard	Anthropometric	Gender	1,64	1.01759	0.19918	0.52255	0.00027
			Smoking	2,63	0.99421	0.61294	0.74753	-0.00018
		Medication	Thiopurine	1,64	1.02091	0.17098	0.52255	0.00032
			Anti-TNF	1,64	0.98432	0.74153	0.74983	-0.00024
			Other medication	1,64	1.00253	0.41516	0.62034	0.00004
		Subtypes	Extent (Sub-E)	2,63	1.00945	0.26127	0.52255	0.00029
UC	Generalized UniFrac	Anthropometric	Gender	1,64	1.01551	0.38206	0.57084	0.00024
			Smoking	2,63	0.88643	0.91921	0.92391	-0.00351
		Medication	Thiopurine	1,64	1.16328	0.10819	0.31857	0.00251
			Anti-TNF	1,64	1.01818	0.37526	0.57084	0.00028
			Other medication	1,64	1.26839	0.0382	0.23398	0.00411
		Subtypes	Extent (Sub-E)	2,63	0.97485	0.56534	0.67841	-0.00077

Appendix Table 3: PERMANOVA results of the effect of anthropometric variables, medication and disease subtypes according to Montreal classification for CD and UC on bacterial community differences in CD and UC. Highlighted cells in the column "*P* value" have a *P* value less than 0.05 whilst highlighted cells in the "P_{FDR}" (adjusted via Benjamini-Hochberg procedure) column have a P_{FDR} value less than 0.1

Phylum	Mean	Stat	P value	P_{FDR}	Direction
Bacteroidetes	5453.87686	1.63809	0.44085	0.68668	-
Firmicutes	5176.34467	14.47254	0.00072	0.00264	UC> CD> Contr.
Proteobacteria	1392.06460	3.41796	0.18105	0.33193	-
Verrucomicrobia	74.06126	51.44171	6.75420×10^{-12}	7.42962×10^{-11}	Contr.> CD> UC
Actinobacteria	227.58876	23.18794	0.00001	0.00005	UC > Contr. > CD
Bacteria uncl.	72.66171	-9.12454	1.00000	1.00000	-
Fusobacteria	2.86444	7.67329	0.02157	0.05931	CD> Contr > UC
Cyanobacteria/Chloroplast	2.15357	1.38869	0.49940	0.68668	-
Synergistetes	0.82165	0.80621	0.66824	0.78139	-
Candidatus Saccharibacteria	0.76557	4.76003	0.09255	0.20361	-
Lentisphaerae	0.60454	0.68398	0.71036	0.78139	-

Appendix Table 4: Analyses of differential phylum abundances with respect to IBD status/health condition.

Highlighted rows have statistically significant differential abundance, with a *P* value less than 0.05 and a P_{FDR} (adjusted via Benjamini-Hochberg procedure) less than 0.1

ASV	Phylum	Organism	Mean	Stat	*P* value	P_{FDR}	Direction
ASV_497	P-Firmicutes	G-Dorea uncl.	4.54	108.47	0.000000	0.000000	CD>Contr/UC
ASV_709	P-Firmicutes	G-Subdoligranulum uncl.	4.06	23.68	0.000007	0.000263	CD>Contr/UC
ASV_6	P-Proteobacteria	G-Escherichia/Shigella uncl.	130.19	23.63	0.000007	0.000263	CD>Contr/UC
ASV_14	P-Bacteroidetes	G-Alistipes uncl.	82.59	23.70	0.000007	0.000263	Contr.>CD/UC
ASV_420	P-Firmicutes	G-Coprococcus uncl.	5.83	18.41	0.000101	0.003038	Contr.>CD/UC
ASV_254	P-Proteobacteria	G-Oxalobacter uncl.	9.13	182.92	0.000000	0.000000	Contr.>CD/UC
ASV_20	P-Verrucomicrobia	S-Akkermansia muciniphila	77.52	60.17	0.000000	0.000000	Contr.>CD/UC
ASV_39	P-Bacteroidetes	G-Bacteroides uncl.	40.89	12.53	0.001900	0.049885	Contr.>CD/UC
ASV_46	P-Firmicutes	F-Lachnospiraceae uncl.	42.20	1044.52	0.000000	0.000000	Contr.>CD/UC
ASV_90	P-Firmicutes	G-Oscillibacter uncl.	24.41	22.85	0.000011	0.000366	Contr.>CD/UC
ASV_182	P-Firmicutes	O-Clostridiales uncl.	14.95	189.06	0.000000	0.000000	Contr.>CD/UC
ASV_227	P-Firmicutes	G-Anaerostipes uncl.	7.58	225.38	0.000000	0.000000	Contr.>CD/UC
ASV_332	P-Firmicutes	O-Clostridiales uncl.	7.72	11.45	0.003265	0.078875	Contr.>CD/UC
ASV_883	P-Firmicutes	G-Faecalibacterium uncl.	2.75	14.00	0.000911	0.025005	Contr.>CD/UC
ASV_38	P-Proteobacteria	S-Haemophilus parainfluenzae	35.61	17.62	0.000149	0.004283	Contr.>CD/UC
ASV_66	P-Proteobacteria	G-Sutterella uncl.	30.18	673.29	0.000000	0.000000	Contr.>CD/UC
ASV_29	P-Firmicutes	S-Dialister invisus	36.30	20.12	0.000043	0.001358	CD/UC>Contr.
ASV_87	P-Firmicutes	S-Flavonifractor plautii	16.62	41.91	0.000000	0.000000	CD/UC>Contr.
ASV_378	P-Firmicutes	G-Oscillibacter uncl.	6.75	80.17	0.000000	0.000000	CD/UC>Contr.
ASV_73	P-Bacteroidetes	G-Bacteroides uncl.	20.97	618.02	0.000000	0.000000	CD/UC>Contr.
ASV_4	P-Firmicutes	G-Acidaminococcus uncl.	178.54	2940.79	0.000000	0.000000	CD/UC>Contr.
ASV_438	P-Firmicutes	F-Ruminococcaceae uncl.	6.48	43.74	0.000000	0.000000	CD/UC>Contr.
ASV_156	P-Bacteroidetes	G-Parabacteroides uncl.	14.20	26.00	0.000002	0.000098	UC>Contr/CD
ASV_373	P-Firmicutes	G-Faecalibacterium uncl.	5.59	102.23	0.000000	0.000000	UC>Contr/CD
ASV_836	P-Proteobacteria	G-Parasutterella uncl.	2.68	11.85	0.002668	0.067153	UC>Contr/CD

Appendix Table 5: Analyses of differential abundances at lower taxonomic levels with respect to IBD status/health condition

ASV	Phylum	Species	Association	Stat	*P* value	P_{FDR}
ASV_16	P-Bacteroidetes	S-Alistipes shahii	Contr.	0.59522	0.00630	0.26291
ASV_641	P-Bacteroidetes	G-Coprobacter uncl.	Contr.	0.45499	0.01640	0.31980
ASV_100	P-Bacteroidetes	S-Alistipes finegoldii	Contr.	0.43808	0.03240	0.34691
ASV_356	P-Bacteroidetes	S-Bacteroides thetaiotaomicron	Contr.	0.40410	0.03010	0.34691
ASV_384	P-Bacteroidetes	S-Butyricimonas faecihominis	Contr.	0.46418	0.03180	0.34691
ASV_58	P-Bacteroidetes	S-Bacteroides fragilis	Contr.	0.43124	0.03900	0.36188
ASV_99	P-Bacteroidetes	S-Alistipes indistinctus	Contr.	0.56801	0.03740	0.36188
ASV_296	P-Firmicutes	G-Oscillibacter uncl.	Contr.	0.47920	0.00040	0.09279
ASV_420	P-Firmicutes	G-Coprococcus uncl.	Contr.	0.51021	0.00050	0.09279
ASV_883	P-Firmicutes	G-Faecalibacterium uncl.	Contr.	0.44300	0.00150	0.17398
ASV_1089	P-Firmicutes	O-Clostridiales uncl.	Contr.	0.46397	0.00250	0.21651
ASV_2013	P-Firmicutes	F-Peptococcaceae 1 uncl.	Contr.	0.44984	0.00280	0.21651
ASV_402	P-Firmicutes	G-Oscillibacter uncl.	Contr.	0.40489	0.00470	0.24229
ASV_534	P-Firmicutes	G-Oscillibacter uncl.	Contr.	0.46460	0.00370	0.24229
ASV_587	P-Firmicutes	G-Faecalibacterium uncl.	Contr.	0.43186	0.00440	0.24229
ASV_553	P-Firmicutes	G-Subdoligranulum uncl.	Contr.	0.41432	0.00540	0.25053
ASV_1916	P-Firmicutes	O-Clostridiales uncl.	Contr.	0.40728	0.00930	0.30930
ASV_321	P-Firmicutes	O-Clostridiales uncl.	Contr.	0.49659	0.01530	0.31980
ASV_595	P-Firmicutes	P-Firmicutes uncl.	Contr.	0.40018	0.01740	0.31980
ASV_759	P-Firmicutes	O-Clostridiales uncl.	Contr.	0.38482	0.01720	0.31980
ASV_2367	P-Firmicutes	G-Oscillibacter uncl.	Contr.	0.36169	0.01930	0.31980
ASV_762	P-Firmicutes	F-Lachnospiraceae uncl.	Contr.	0.47540	0.02200	0.32926
ASV_989	P-Firmicutes	O-Clostridiales uncl.	Contr.	0.38073	0.02320	0.33637
ASV_625	P-Firmicutes	P-Firmicutes uncl.	Contr.	0.43357	0.02500	0.34387
ASV_90	P-Firmicutes	G-Oscillibacter uncl.	Contr.	0.47952	0.02760	0.34691
ASV_256	P-Firmicutes	F-Lachnospiraceae uncl.	Contr.	0.53884	0.03250	0.34691
ASV_665	P-Firmicutes	F-Lachnospiraceae uncl.	Contr.	0.37480	0.02820	0.34691
ASV_3007	P-Firmicutes	G-Clostridium IV uncl.	Contr.	0.36093	0.03160	0.34691

ASV_196	P-Firmicutes	O-Clostridiales uncl.	Contr.	0.36241	0.04180	0.37295
ASV_1238	P-Firmicutes	G-Ruminococcus uncl.	Contr.	0.33015	0.04850	0.40596
ASV_20	P-Verrucomicrobia	S-Akkermansia muciniphila	Contr.	0.50230	0.01300	0.30930
ASV_114	P-Bacteroidetes	S-Bacteroides fragilis	CD	0.41397	0.01330	0.30930
ASV_281	P-Bacteroidetes	S-Alistipes massiliensis	CD	0.44812	0.00940	0.30930
ASV_27	P-Bacteroidetes	G-Bacteroides uncl.	CD	0.43769	0.02620	0.34691
ASV_42	P-Bacteroidetes	G-Bacteroides uncl.	CD	0.44754	0.04540	0.39742
ASV_828	P-Firmicutes	F-Lachnospiraceae uncl.	CD	0.41126	0.01310	0.30930
ASV_70	P-Firmicutes	S-Lactobacillus gasseri	CD	0.42151	0.01850	0.31980
ASV_620	P-Firmicutes	G-Butyricicoccus uncl.	CD	0.48866	0.02060	0.32926
ASV_951	P-Firmicutes	G-Faecalibacterium uncl.	CD	0.42615	0.02160	0.32926
ASV_152	P-Firmicutes	G-Oscillibacter uncl.	CD	0.54499	0.03020	0.34691
ASV_29	P-Firmicutes	S-Dialister invisus	CD	0.38251	0.03850	0.36188
ASV_24	P-Proteobacteria	G-Escherichia/Shigella uncl.	CD	0.58834	0.00060	0.09279
ASV_6	P-Proteobacteria	G-Escherichia/Shigella uncl.	CD	0.61006	0.00680	0.26291
ASV_30	P-Proteobacteria	F-Enterobacteriaceae uncl.	CD	0.52840	0.01060	0.30930
ASV_648	P-Actinobacteria	S-Eggerthella lenta	UC	0.48262	0.02520	0.34387
ASV_143	P-Bacteroidetes	G-Odoribacter uncl.	UC	0.51498	0.03390	0.34951
ASV_4	P-Firmicutes	G-Acidaminococcus uncl.	UC	0.54701	0.01190	0.30930
ASV_440	P-Firmicutes	F-Lachnospiraceae uncl.	UC	0.49237	0.01400	0.30930
ASV_698	P-Firmicutes	F-Lachnospiraceae uncl.	UC	0.40527	0.01340	0.30930
ASV_2191	P-Firmicutes	F-Ruminococcaceae uncl.	UC	0.37475	0.01910	0.31980
ASV_977	P-Firmicutes	G-Romboutsia uncl.	UC	0.35837	0.03290	0.34691
ASV_1347	P-Firmicutes	F-Ruminococcaceae uncl.	UC	0.34871	0.03550	0.35805
ASV_696	P-Firmicutes	G-Blautia uncl.	UC	0.40196	0.03790	0.36188
ASV_647	P-Firmicutes	F-Ruminococcaceae uncl.	UC	0.34596	0.04040	0.36752

Appendix Table 6: Species indicator analysis for controls, CD and UC.

Highlighted rows can be considered as indicator species as they have a *P* value less than 0.05 and a P_{FDR} (adjusted via Benjamini-Hochberg procedure) less than 0.1

Phenotype	ASV	Mean	Phylum	Species	Stat	*P* value	P_{FDR}	Direction
A	ASV_3	189.71685	P-Bacteroidetes	S-Bacteroides-uniformis	208.579727	0.000000	0.000000	A2<A1,A3<A1,A3<A2
A	ASV_42	88.30275	P-Bacteroidetes	G-Bacteroides uncl.	12.914171	0.001569	0.012707	
A	ASV_57	16.83634	P-Firmicutes	G-Megamonas uncl.	12.163580	0.002284	0.017114	
A	ASV_60	14.19672	P-Actinobacteria	S-Collinsella-aerofaciens	46.317133	0.000000	0.000000	
A	ASV_133	53.75416	P-Firmicutes	S-Dialister-succinatiphilus	76.787796	0.000000	0.000000	
A	ASV_173	25.56731	P-Proteobacteria	G-Citrobacter uncl.	13.242794	0.001332	0.011109	
A	ASV_212	13.75428	P-Firmicutes	G-Streptococcus uncl.	31.647130	0.000000	0.000003	
A	ASV_247	12.64526	P-Bacteroidetes	S-Bacteroides-ovatus	19.214355	0.000067	0.000866	
A	ASV_270	6.05590	P-Bacteroidetes	G-Bacteroides uncl.	18.425628	0.000100	0.001162	
A	ASV_302	17.27634	P-Firmicutes	G-Dialister uncl.	40.896753	0.000000	0.000000	
A	ASV_311	20.19449	P-Bacteroidetes	G-Parabacteroides uncl.	56.667668	0.000000	0.000000	
A	ASV_390	4.47821	P-Bacteroidetes	G-Barnesiella uncl.	18.717521	0.000086	0.001056	
A	ASV_493	16.54697	P-Firmicutes	G-Streptococcus uncl.	23.103198	0.000010	0.000151	
A	ASV_658	8.17686	P-Actinobacteria	S-Collinsella-intestinalis	12.282386	0.002152	0.016371	
A	ASV_51	74.22143	P-Bacteroidetes	G-Barnesiella uncl.	32.760439	0.000000	0.000002	A2<A1,A3<A1,A3>A2
A	ASV_291	35.92474	P-Bacteroidetes	G-Bacteroides uncl.	49.817732	0.000000	0.000000	
A	ASV_375	27.62599	P-Bacteroidetes	G-Prevotella uncl.	16.903410	0.000214	0.002281	
A	ASV_413	5.27628	P-Firmicutes	F-Ruminococcaceae uncl.	11.659236	0.002939	0.021384	
A	ASV_480	9.14567	P-Firmicutes	G-Roseburia uncl.	15.057287	0.000537	0.005189	
A	ASV_659	6.95994	P-Proteobacteria	F-Enterobacteriaceae uncl.	19.261733	0.000066	0.000866	
A	ASV_760	7.43462	P-Proteobacteria	G-Escherichia/Shigella uncl.	19.758156	0.000051	0.000714	
A	ASV_17	29.72181	P-Bacteroidetes	G-Bacteroides uncl.	18.441866	0.000099	0.001162	A2<A1,A3>A1,A3>A2
A	ASV_26	26.66861	P-Bacteroidetes	S-Alistipes-onderdonkii	16.839986	0.000220	0.002305	
A	ASV_27	62.36070	P-Bacteroidetes	G-Bacteroides uncl.	14.527293	0.000701	0.006513	
A	ASV_54	36.80121	P-Bacteroidetes	G-Bacteroides uncl.	23.821106	0.000007	0.000112	
A	ASV_109	5.27331	P-Firmicutes	G-Holdemanella uncl.	10.024886	0.006655	0.047051	
A	ASV_113	39.56234	P-Firmicutes	F-Ruminococcaceae uncl.	14.477607	0.000718	0.006555	
A	ASV_119	15.41636	P-Bacteroidetes	S-Bacteroides-cellulosilyticus	13.546888	0.001144	0.009899	
A	ASV_140	23.93240	P-Firmicutes	G-Phascolarctobacterium uncl.	13.784218	0.001016	0.009106	

A	ASV_144	28.32549	P-Bacteroidetes	G-Prevotella uncl.	34.535139	0.000000	0.000001	
A	ASV_160	3.65863	P-Bacteroidetes	G-Bacteroides uncl.	10.079069	0.006477	0.046448	
A	ASV_200	43.11352	P-Bacteroidetes	G-Bacteroides uncl.	24.262135	0.000005	0.000093	
A	ASV_209	11.79678	P-Firmicutes	G-Subdoligranulum uncl.	35.590023	0.000000	0.000001	
A	ASV_336	11.97130	P-Firmicutes	G-Holdemanella uncl.	15.082334	0.000531	0.005189	
A	ASV_459	15.55499	P-Firmicutes	P-Firmicutes uncl.	12.518001	0.001913	0.015006	
A	ASV_583	4.50533	P-Firmicutes	G-Ruminococcus2 uncl.	15.565696	0.000417	0.004185	
A	ASV_836	5.78610	P-Proteobacteria	G-Parasutterella uncl.	12.010968	0.002465	0.018199	
A	ASV_847	8.79736	P-Firmicutes	G-Catenibacterium uncl.	24.569987	0.000005	0.000083	
A	ASV_1270	4.74114	P-Bacteroidetes	G-Alloprevotella uncl.	14.681596	0.000649	0.006143	
A	ASV_1329	6.05348	P-Bacteroidetes	G-Prevotella uncl.	18.896366	0.000079	0.000989	
A	ASV_8	101.33687	P-Firmicutes	G-Dialister uncl.	41.514175	0.000000	0.000000	A2>A1,A3<A1,A3<A2
A	ASV_35	40.38527	P-Bacteroidetes	S-Parabacteroides-distasonis	81.820263	0.000000	0.000000	
A	ASV_59	12.31894	P-Actinobacteria	S-Collinsella-aerofaciens	19.315222	0.000064	0.000866	
A	ASV_66	36.81866	P-Proteobacteria	G-Sutterella uncl.	23.146009	0.000009	0.000151	
A	ASV_86	55.92837	P-Firmicutes	S-Dialister-invisus	17.311821	0.000174	0.001924	
A	ASV_131	110.42208	P-Bacteroidetes	G-Bacteroides uncl.	38.309171	0.000000	0.000000	
A	ASV_142	21.58774	P-Actinobacteria	S-Collinsella-aerofaciens	12.879800	0.001597	0.012722	
A	ASV_146	26.51159	P-Bacteroidetes	G-Bacteroides uncl.	19.814023	0.000050	0.000714	
A	ASV_226	15.26128	P-Firmicutes	G-Ruminococcus2 uncl.	47.954538	0.000000	0.000000	
A	ASV_265	12.44200	P-Firmicutes	S-Dorea-longicatena	38.082170	0.000000	0.000000	
A	ASV_363	26.54383	P-Bacteroidetes	G-Bacteroides uncl.	72.267650	0.000000	0.000000	
A	ASV_423	4.04494	P-Firmicutes	F-Lachnospiraceae uncl.	12.437725	0.001992	0.015381	
A	ASV_473	7.25661	P-Bacteroidetes	G-Bacteroides uncl.	27.986086	0.000001	0.000017	
A	ASV_2822	3.75788	P-Firmicutes	G-Faecalibacterium uncl.	13.605052	0.001111	0.009784	
A	ASV_10	80.22210	P-Bacteroidetes	S-Bacteroides-uniformis	36.946024	0.000000	0.000000	A2>A1,A3>A1,A3<A2
A	ASV_20	62.05206	P-Verrucomicrobia	S-Akkermansia-muciniphila	25.872233	0.000002	0.000045	
A	ASV_23	83.57133	P-Bacteroidetes	G-Prevotella uncl.	20.586176	0.000034	0.000500	
A	ASV_158	17.09769	P-Bacteroidetes	S-Prevotella-copri	18.384100	0.000102	0.001162	
A	ASV_228	14.80294	P-Firmicutes	S-Faecalibacterium-prausnitzii	47.149156	0.000000	0.000000	
A	ASV_828	8.97145	P-Firmicutes	F-Lachnospiraceae uncl.	22.601910	0.000012	0.000188	

A	ASV_15	149.91432	P-Firmicutes	S-Dialister-invisus	15.575465	0.000415	0.004185	A2>A1,A3>A1,A3>A2
A	ASV_18	34.26563	P-Bacteroidetes	G-Bacteroides uncl.	27.391659	0.000001	0.000022	
A	ASV_19	46.49237	P-Firmicutes	F-Lachnospiraceae uncl.	97.258150	0.000000	0.000000	
A	ASV_29	79.28204	P-Firmicutes	S-Dialister-invisus	31.242254	0.000000	0.000004	
A	ASV_36	61.06020	P-Bacteroidetes	S-Bacteroides-dorei	56.669887	0.000000	0.000000	
A	ASV_75	24.85366	P-Proteobacteria	G-Klebsiella uncl.	13.454190	0.001198	0.010193	
A	ASV_78	27.93286	P-Firmicutes	G-Phascolarctobacterium uncl.	17.286960	0.000176	0.001924	
A	ASV_94	26.99081	P-Bacteroidetes	S-Bacteroides-vulgatus	54.489569	0.000000	0.000000	
A	ASV_157	29.03295	P-Bacteroidetes	S-Bacteroides-clarus	9.905626	0.007064	0.049248	
A	ASV_219	56.21588	P-Proteobacteria	G-Enterobacter uncl.	29.191994	0.000000	0.000010	
A	ASV_259	32.06328	P-Bacteroidetes	S-Bacteroides-finegoldii	13.215380	0.001350	0.011109	
A	ASV_527	12.64440	P-Bacteroidetes	G-Prevotella uncl.	32.390837	0.000000	0.000002	
B	ASV_17	29.72181	P-Bacteroidetes	G-Bacteroides uncl.	79.270850	0.000000	0.000000	B2<B1,B3<B1,B3<B2
B	ASV_23	83.57133	P-Bacteroidetes	G-Prevotella uncl.	39.846889	0.000000	0.000000	
B	ASV_35	40.38527	P-Bacteroidetes	S-Parabacteroides-distasonis	77.934731	0.000000	0.000000	
B	ASV_86	55.92837	P-Firmicutes	S-Dialister-invisus	19.161037	0.000069	0.000738	
B	ASV_137	22.66353	P-Bacteroidetes	S-Alistipes-finegoldii	13.633107	0.001095	0.008332	
B	ASV_178	10.82023	P-Firmicutes	G-Holdemanella uncl.	30.013120	0.000000	0.000005	
B	ASV_200	43.11352	P-Bacteroidetes	G-Bacteroides uncl.	31.849766	0.000000	0.000002	
B	ASV_221	7.23754	P-Bacteroidetes	G-Alistipes uncl.	19.244144	0.000066	0.000723	
B	ASV_224	8.50762	P-Firmicutes	G-Faecalibacterium uncl.	24.209665	0.000006	0.000068	
B	ASV_228	14.80294	P-Firmicutes	S-Faecalibacterium-prausnitzii	49.853322	0.000000	0.000000	
B	ASV_288	6.32319	P-Firmicutes	G-Faecalibacterium uncl.	12.815105	0.001649	0.012174	
B	ASV_291	35.92474	P-Bacteroidetes	G-Bacteroides uncl.	29.661248	0.000000	0.000006	
B	ASV_359	9.02824	P-Firmicutes	G-Dialister uncl.	34.049209	0.000000	0.000001	
B	ASV_497	10.15160	P-Firmicutes	G-Dorea uncl.	10.727475	0.004683	0.029388	
B	ASV_527	12.64440	P-Bacteroidetes	G-Prevotella uncl.	11.439983	0.003280	0.021382	
B	ASV_583	4.50533	P-Firmicutes	G-Ruminococcus2 uncl.	21.310790	0.000024	0.000275	
B	ASV_633	9.39460	P-Bacteroidetes	G-Bacteroides uncl.	18.785431	0.000083	0.000837	
B	ASV_663	5.20826	P-Firmicutes	S-Megasphaera-massiliensis	15.753466	0.000379	0.003072	
B	ASV_700	5.43721	P-Firmicutes	G-Megasphaera uncl.	16.625651	0.000245	0.002161	

B	ASV_829	4.20717	P-Firmicutes	G-Megasphaera uncl.	12.286740	0.002148	0.015185	
B	ASV_837	4.17246	P-Firmicutes	G-Megasphaera uncl.	12.709615	0.001738	0.012647	
B	ASV_1605	7.31292	P-Bacteroidetes	G-Bacteroides uncl.	17.765451	0.000139	0.001314	
B	ASV_14	131.39524	P-Bacteroidetes	G-Alistipes uncl.	18.577498	0.000092	0.000910	B2<B1,B3<B1,B3>B2
B	ASV_29	79.28204	P-Firmicutes	S-Dialister-invisus	29.173525	0.000000	0.000007	
B	ASV_511	9.79593	P-Firmicutes	G-Subdoligranulum uncl.	32.994198	0.000000	0.000001	
B	ASV_15	149.91432	P-Firmicutes	S-Dialister-invisus	30.403879	0.000000	0.000004	B2<B1,B3>B1,B3>B2
B	ASV_25	57.17168	P-Firmicutes	G-Subdoligranulum uncl.	155.362318	0.000000	0.000000	
B	ASV_26	26.66861	P-Bacteroidetes	S-Alistipes-onderdonkii	11.493336	0.003193	0.021093	
B	ASV_31	30.83048	P-Proteobacteria	G-Escherichia/Shigella uncl.	34.922907	0.000000	0.000001	
B	ASV_42	88.30275	P-Bacteroidetes	G-Bacteroides uncl.	17.540505	0.000155	0.001444	
B	ASV_53	83.04174	P-Proteobacteria	G-Parasutterella uncl.	92.361196	0.000000	0.000000	
B	ASV_67	10.35938	P-Bacteroidetes	S-Bacteroides-thetaiotaomicron	19.095979	0.000071	0.000746	
B	ASV_69	14.30750	P-Proteobacteria	G-Parasutterella uncl.	44.617236	0.000000	0.000000	
B	ASV_80	31.63949	P-Bacteroidetes	S-Bacteroides-thetaiotaomicron	86.491861	0.000000	0.000000	
B	ASV_95	11.27460	P-Firmicutes	F-Ruminococcaceae uncl.	33.168170	0.000000	0.000001	
B	ASV_101	19.13745	P-Proteobacteria	G-Klebsiella uncl.	56.255501	0.000000	0.000000	
B	ASV_157	29.03295	P-Bacteroidetes	S-Bacteroides-clarus	65.269856	0.000000	0.000000	
B	ASV_159	28.21102	P-Firmicutes	F-Ruminococcaceae uncl.	33.170136	0.000000	0.000001	
B	ASV_219	56.21588	P-Proteobacteria	G-Enterobacter uncl.	51.350832	0.000000	0.000000	
B	ASV_504	3.62849	P-Firmicutes	F-Ruminococcaceae uncl.	11.043490	0.003999	0.025410	
B	ASV_517	5.59885	P-Bacteroidetes	G-Alistipes uncl.	16.559029	0.000254	0.002195	
B	ASV_584	7.94324	P-Firmicutes	S-Dialister-invisus	28.349018	0.000001	0.000009	
B	ASV_820	3.17836	P-Proteobacteria	G-Oxalobacter uncl.	15.200577	0.000500	0.003987	
B	ASV_836	5.78610	P-Proteobacteria	G-Parasutterella uncl.	16.981002	0.000205	0.001841	
B	ASV_860	6.57191	P-Firmicutes	G-Clostridium-IV uncl.	17.342643	0.000171	0.001565	
B	ASV_11	76.70738	P-Bacteroidetes	G-Bacteroides uncl.	13.911353	0.000953	0.007362	B2>B1,B3<B1,B3<B2
B	ASV_40	70.67344	P-Bacteroidetes	G-Paraprevotella uncl.	25.594827	0.000003	0.000037	
B	ASV_58	3.96400	P-Bacteroidetes	S-Bacteroides-fragilis	14.777077	0.000618	0.004850	
B	ASV_78	27.93286	P-Firmicutes	G-Phascolarctobacterium uncl.	35.409388	0.000000	0.000000	
B	ASV_108	47.33334	P-Bacteroidetes	G-Bacteroides uncl.	18.307227	0.000106	0.001022	

B	ASV_131	110.42208	P-Bacteroidetes	G-Bacteroides uncl.	28.960468	0.000001	0.000007	
B	ASV_133	53.75416	P-Firmicutes	S-Dialister-succinatiphilus	145.874215	0.000000	0.000000	
B	ASV_142	21.58774	P-Actinobacteria	S-Collinsella-aerofaciens	29.898224	0.000000	0.000005	
B	ASV_167	10.08626	P-Firmicutes	S-Roseburia-faecis	11.049444	0.003987	0.025410	
B	ASV_217	15.67417	P-Firmicutes	G-Oscillibacter uncl.	13.571697	0.001130	0.008464	
B	ASV_226	15.26128	P-Firmicutes	G-Ruminococcus2 uncl.	47.715992	0.000000	0.000000	
B	ASV_243	11.97483	P-Firmicutes	S-Roseburia-hominis	34.259063	0.000000	0.000001	
B	ASV_265	12.44200	P-Firmicutes	S-Dorea-longicatena	40.316099	0.000000	0.000000	
B	ASV_282	35.33819	P-Proteobacteria	G-Escherichia/Shigella uncl.	87.122665	0.000000	0.000000	
B	ASV_292	10.94616	P-Bacteroidetes	S-Bacteroides-thetaiotaomicron	39.091947	0.000000	0.000000	
B	ASV_331	9.67979	P-Firmicutes	O-Clostridiales uncl.	11.521129	0.003149	0.021080	
B	ASV_358	7.16069	P-Bacteroidetes	G-Bacteroides uncl.	12.335862	0.002096	0.015028	
B	ASV_363	26.54383	P-Bacteroidetes	G-Bacteroides uncl.	81.161064	0.000000	0.000000	
B	ASV_422	17.42229	P-Proteobacteria	S-Escherichia/Shigella-coli	44.442654	0.000000	0.000000	
B	ASV_555	10.30578	P-Bacteroidetes	S-Bacteroides-intestinalis	11.831129	0.002697	0.018380	
B	ASV_575	8.91137	P-Bacteroidetes	G-Parabacteroides uncl.	19.571752	0.000056	0.000627	
B	ASV_22	49.05799	P-Proteobacteria	G-Parasutterella uncl.	16.501407	0.000261	0.002221	B2>B1,B3>B1,B3<B2
B	ASV_51	74.22143	P-Bacteroidetes	G-Barnesiella uncl.	60.982971	0.000000	0.000000	
B	ASV_54	36.80121	P-Bacteroidetes	G-Bacteroides uncl.	9.805806	0.007425	0.044908	
B	ASV_57	16.83634	P-Firmicutes	G-Megamonas uncl.	10.696618	0.004756	0.029477	
B	ASV_172	7.53794	P-Firmicutes	G-Phascolarctobacterium uncl.	11.822019	0.002709	0.018380	
B	ASV_259	32.06328	P-Bacteroidetes	S-Bacteroides-finegoldii	16.019855	0.000332	0.002779	
B	ASV_375	27.62599	P-Bacteroidetes	G-Prevotella uncl.	9.592126	0.008262	0.049377	
B	ASV_383	8.23577	P-Bacteroidetes	G-Parabacteroides uncl.	29.466581	0.000000	0.000006	
B	ASV_847	8.79736	P-Firmicutes	G-Catenibacterium uncl.	10.016477	0.006683	0.040911	
B	ASV_13	87.75980	P-Proteobacteria	G-Escherichia/Shigella uncl.	22.426999	0.000013	0.000161	B2>B1,B3>B1,B3>B2
B	ASV_75	24.85366	P-Proteobacteria	G-Klebsiella uncl.	24.230029	0.000005	0.000068	
B	ASV_113	39.56234	P-Firmicutes	F-Ruminococcaceae uncl.	44.808336	0.000000	0.000000	
B	ASV_198	5.27175	P-Firmicutes	G-Oscillibacter uncl.	20.702772	0.000032	0.000365	
B	ASV_212	13.75428	P-Firmicutes	G-Streptococcus uncl.	31.314747	0.000000	0.000003	
B	ASV_266	8.89045	P-Proteobacteria	G-Bilophila uncl.	11.851180	0.002670	0.018380	

B	ASV_372	4.90039	P-Bacteroidetes	G-Alistipes uncl.	18.804578	0.000083	0.000837	
B	ASV_493	16.54697	P-Firmicutes	G-Streptococcus uncl.	25.287443	0.000003	0.000042	
B	ASV_514	9.92883	P-Firmicutes	O-Clostridiales uncl.	15.820695	0.000367	0.003020	
E	ASV_29	48.05438	P-Firmicutes	S-Dialister-invisus	51.269270	0.000000	0.000000	E2<E1,E3<E1,E3<E2
E	ASV_326	5.68406	P-Firmicutes	F-Ruminococcaceae uncl.	18.464894	0.000098	0.002222	
E	ASV_6	49.58314	P-Proteobacteria	G-Escherichia/Shigella uncl.	11.565491	0.003080	0.048976	E2<E1,E3<E1,E3>E2
E	ASV_13	40.96810	P-Proteobacteria	G-Escherichia/Shigella uncl.	12.226725	0.002213	0.037699	
E	ASV_77	23.53274	P-Proteobacteria	S-Haemophilus-parainfluenzae	19.806635	0.000050	0.001325	
E	ASV_290	8.27016	P-Firmicutes	G-Faecalibacterium uncl.	54.784523	0.000000	0.000000	
E	ASV_323	14.52845	P-Firmicutes	G-Oscillibacter uncl.	21.914223	0.000017	0.000520	
E	ASV_555	3.06671	P-Bacteroidetes	S-Bacteroides-intestinalis	11.494075	0.003192	0.049119	
E	ASV_24	28.80730	P-Proteobacteria	G-Escherichia/Shigella uncl.	12.250523	0.002187	0.037699	E2<E1,E3>E1,E3>E2
E	ASV_30	21.73724	P-Proteobacteria	F-Enterobacteriaceae uncl.	14.103552	0.000866	0.016521	
E	ASV_40	38.16365	P-Bacteroidetes	G-Paraprevotella uncl.	147.316836	0.000000	0.000000	
E	ASV_78	20.68871	P-Firmicutes	G-Phascolarctobacterium uncl.	15.572122	0.000415	0.008258	
E	ASV_85	18.92772	P-Firmicutes	G-Dialister uncl.	12.423721	0.002006	0.036793	
E	ASV_107	30.50817	P-Firmicutes	G-Faecalibacterium uncl.	191.428384	0.000000	0.000000	
E	ASV_110	38.62665	P-Proteobacteria	S-Escherichia/Shigella-coli	17.775942	0.000138	0.002993	
E	ASV_122	15.92610	P-Firmicutes	S-Anaerostipes-hadrus	137.175958	0.000000	0.000000	
E	ASV_588	6.38008	P-Firmicutes	F-Ruminococcaceae uncl.	20.450642	0.000036	0.001017	
E	ASV_8	175.98817	P-Firmicutes	G-Dialister uncl.	118.662243	0.000000	0.000000	E2>E1,E3<E1,E3<E2
E	ASV_28	76.37325	P-Bacteroidetes	G-Barnesiella uncl.	51.730026	0.000000	0.000000	
E	ASV_87	26.68811	P-Firmicutes	S-Flavonifractor-plautii	19.670012	0.000054	0.001344	
E	ASV_146	19.35994	P-Bacteroidetes	G-Bacteroides uncl.	97.747962	0.000000	0.000000	
E	ASV_563	5.32369	P-Bacteroidetes	G-Bacteroides uncl.	18.733356	0.000086	0.002040	
E	ASV_46	30.68286	P-Firmicutes	F-Lachnospiraceae uncl.	175.087215	0.000000	0.000000	E2>E1,E3>E1,E3<E2
E	ASV_49	49.13892	P-Firmicutes	G-Ruminococcus uncl.	37.216664	0.000000	0.000000	
E	ASV_169	9.43471	P-Bacteroidetes	G-Alistipes uncl.	42.672046	0.000000	0.000000	
E	ASV_206	11.43925	P-Firmicutes	G-Blautia uncl.	100.063563	0.000000	0.000000	
E	ASV_17	85.52746	P-Bacteroidetes	G-Bacteroides uncl.	70.934878	0.000000	0.000000	E2>E1,E3>E1,E3>E2

E	ASV_23	55.18226	P-Bacteroidetes	G-Prevotella uncl.	48.798769	0.000000	0.000000	
E	ASV_42	30.61443	P-Bacteroidetes	G-Bacteroides uncl.	12.156672	0.002292	0.037699	
E	ASV_126	23.60610	P-Firmicutes	F-Lachnospiraceae uncl.	16.544462	0.000256	0.005299	
E	ASV_175	11.23955	P-Firmicutes	S-Streptococcus-salivarius	84.552836	0.000000	0.000000	
L	ASV_14	131.39524	P-Bacteroidetes	G-Alistipes uncl.	15.643988	0.000401	0.003565	L2<L1,L3<L1,L3<L2
L	ASV_160	3.65863	P-Bacteroidetes	G-Bacteroides uncl.	10.152054	0.006245	0.040729	
L	ASV_258	19.97560	P-Proteobacteria	F-Desulfovibrionaceae uncl.	10.624222	0.004932	0.033273	
L	ASV_266	8.89045	P-Proteobacteria	G-Bilophila uncl.	9.622099	0.008139	0.049769	
L	ASV_291	35.92474	P-Bacteroidetes	G-Bacteroides uncl.	37.365289	0.000000	0.000000	
L	ASV_378	12.10670	P-Firmicutes	G-Oscillibacter uncl.	16.703869	0.000236	0.002270	
L	ASV_514	9.92883	P-Firmicutes	O-Clostridiales uncl.	20.476464	0.000036	0.000412	
L	ASV_633	9.39460	P-Bacteroidetes	G-Bacteroides uncl.	23.692039	0.000007	0.000093	
L	ASV_2	304.13659	P-Bacteroidetes	S-Alistipes-putredinis	250.558980	0.000000	0.000000	L2<L1,L3<L1,L3>L2
L	ASV_13	87.75980	P-Proteobacteria	G-Escherichia/Shigella uncl.	21.548728	0.000021	0.000251	
L	ASV_16	42.36141	P-Bacteroidetes	S-Alistipes-shahii	48.325182	0.000000	0.000000	
L	ASV_36	61.06020	P-Bacteroidetes	S-Bacteroides-dorei	42.861379	0.000000	0.000000	
L	ASV_40	70.67344	P-Bacteroidetes	G-Paraprevotella uncl.	24.230089	0.000005	0.000075	
L	ASV_60	14.19672	P-Actinobacteria	S-Collinsella-aerofaciens	51.132171	0.000000	0.000000	
L	ASV_80	31.63949	P-Bacteroidetes	S-Bacteroides-thetaiotaomicron	88.715429	0.000000	0.000000	
L	ASV_94	26.99081	P-Bacteroidetes	S-Bacteroides-vulgatus	52.928337	0.000000	0.000000	
L	ASV_111	9.14257	P-Bacteroidetes	G-Bacteroides uncl.	16.061363	0.000325	0.003031	
L	ASV_131	110.42208	P-Bacteroidetes	G-Bacteroides uncl.	19.763079	0.000051	0.000577	
L	ASV_172	7.53794	P-Firmicutes	G-Phascolarctobacterium uncl.	14.241282	0.000808	0.006589	
L	ASV_173	25.56731	P-Proteobacteria	G-Citrobacter uncl.	79.200873	0.000000	0.000000	
L	ASV_243	11.97483	P-Firmicutes	S-Roseburia-hominis	36.085521	0.000000	0.000000	
L	ASV_247	12.64526	P-Bacteroidetes	S-Bacteroides-ovatus	15.461175	0.000439	0.003848	
L	ASV_282	35.33819	P-Proteobacteria	G-Escherichia/Shigella uncl.	22.468641	0.000013	0.000165	
L	ASV_363	26.54383	P-Bacteroidetes	G-Bacteroides uncl.	38.178278	0.000000	0.000000	
L	ASV_422	17.42229	P-Proteobacteria	S-Escherichia/Shigella-coli	11.211538	0.003677	0.026644	
L	ASV_493	16.54697	P-Firmicutes	G-Streptococcus uncl.	15.119424	0.000521	0.004498	
L	ASV_17	29.72181	P-Bacteroidetes	G-Bacteroides uncl.	89.150285	0.000000	0.000000	L2<L1,L3>L1,L3>L2

L	ASV_22	49.05799	P-Proteobacteria	G-Parasutterella uncl.	119.517335	0.000000	0.000000	
L	ASV_53	83.04174	P-Proteobacteria	G-Parasutterella uncl.	73.335026	0.000000	0.000000	
L	ASV_101	19.13745	P-Proteobacteria	G-Klebsiella uncl.	12.895142	0.001584	0.012568	
L	ASV_113	39.56234	P-Firmicutes	F-Ruminococcaceae uncl.	14.817719	0.000606	0.005154	
L	ASV_219	56.21588	P-Proteobacteria	G-Enterobacter uncl.	54.657435	0.000000	0.000000	
L	ASV_246	6.37084	P-Firmicutes	G-Roseburia uncl.	12.945354	0.001545	0.012424	
L	ASV_249	11.36984	P-Proteobacteria	G-Parasutterella uncl.	10.064336	0.006525	0.041630	
L	ASV_259	32.06328	P-Bacteroidetes	S-Bacteroides-finegoldii	25.566247	0.000003	0.000039	
L	ASV_288	6.32319	P-Firmicutes	G-Faecalibacterium uncl.	12.503625	0.001927	0.014690	
L	ASV_292	10.94616	P-Bacteroidetes	S-Bacteroides-thetaiotaomicron	38.322502	0.000000	0.000000	
L	ASV_348	7.18832	P-Bacteroidetes	S-Odoribacter-splanchnicus	17.070776	0.000196	0.001921	
L	ASV_352	6.00618	P-Bacteroidetes	G-Bacteroides uncl.	17.077656	0.000196	0.001921	
L	ASV_359	9.02824	P-Firmicutes	G-Dialister uncl.	10.654900	0.004856	0.033148	
L	ASV_473	7.25661	P-Bacteroidetes	G-Bacteroides uncl.	28.160190	0.000001	0.000013	
L	ASV_480	9.14567	P-Firmicutes	G-Roseburia uncl.	12.370390	0.002060	0.015501	
L	ASV_510	10.21546	P-Proteobacteria	G-Parasutterella uncl.	11.225627	0.003651	0.026644	
L	ASV_1309	10.82900	P-Proteobacteria	G-Sutterella uncl.	17.340804	0.000172	0.001799	
L	ASV_1709	2.70813	P-Firmicutes	O-Clostridiales uncl.	11.186085	0.003724	0.026656	
L	ASV_8	101.33687	P-Firmicutes	G-Dialister uncl.	17.104545	0.000193	0.001921	L2>L1,L3<L1,L3<L2
L	ASV_20	62.05206	P-Verrucomicrobia	S-Akkermansia-muciniphila	22.030068	0.000016	0.000201	
L	ASV_23	83.57133	P-Bacteroidetes	G-Prevotella uncl.	98.522939	0.000000	0.000000	
L	ASV_29	79.28204	P-Firmicutes	S-Dialister-invisus	115.648650	0.000000	0.000000	
L	ASV_86	55.92837	P-Firmicutes	S-Dialister-invisus	86.411234	0.000000	0.000000	
L	ASV_96	23.38052	P-Firmicutes	F-Ruminococcaceae uncl.	9.927452	0.006987	0.044100	
L	ASV_119	15.41636	P-Bacteroidetes	S-Bacteroides-cellulosilyticus	60.653917	0.000000	0.000000	
L	ASV_158	17.09769	P-Bacteroidetes	S-Prevotella-copri	19.173451	0.000069	0.000752	
L	ASV_207	12.00106	P-Firmicutes	G-Oscillibacter uncl.	11.084116	0.003918	0.027383	
L	ASV_210	12.87547	P-Proteobacteria	F-Enterobacteriaceae uncl.	14.460255	0.000724	0.006075	
L	ASV_211	2.53080	P-Proteobacteria	G-Citrobacter uncl.	10.186352	0.006138	0.040486	
L	ASV_226	15.26128	P-Firmicutes	G-Ruminococcus2 uncl.	51.686823	0.000000	0.000000	
L	ASV_228	14.80294	P-Firmicutes	S-Faecalibacterium-prausnitzii	49.880289	0.000000	0.000000	

L	ASV_248	15.59033	P-Firmicutes	G-Ruminococcus uncl.	10.671570	0.004816	0.033148	
L	ASV_255	34.57826	P-Bacteroidetes	G-Prevotella uncl.	52.833068	0.000000	0.000000	
L	ASV_448	11.91290	P-Firmicutes	G-Subdoligranulum uncl.	51.552657	0.000000	0.000000	
L	ASV_458	8.41102	P-Bacteroidetes	G-Bacteroides uncl.	20.541486	0.000035	0.000407	
L	ASV_497	10.15160	P-Firmicutes	G-Dorea uncl.	9.624011	0.008132	0.049769	
L	ASV_527	12.64440	P-Bacteroidetes	G-Prevotella uncl.	9.679301	0.007910	0.049394	
L	ASV_583	4.50533	P-Firmicutes	G-Ruminococcus2 uncl.	24.090995	0.000006	0.000078	
L	ASV_15	149.91432	P-Firmicutes	S-Dialister-invisus	26.667095	0.000002	0.000026	L2>L1,L3>L1,L3<L2
L	ASV_18	34.26563	P-Bacteroidetes	G-Bacteroides uncl.	36.888948	0.000000	0.000000	
L	ASV_83	20.05108	P-Bacteroidetes	G-Bacteroides uncl.	17.073219	0.000196	0.001921	
L	ASV_114	67.15736	P-Bacteroidetes	S-Bacteroides-fragilis	85.700366	0.000000	0.000000	
L	ASV_120	15.22814	P-Proteobacteria	G-Parasutterella uncl.	11.109351	0.003869	0.027365	
L	ASV_168	53.57739	P-Firmicutes	F-Ruminococcaceae uncl.	27.481267	0.000001	0.000018	
L	ASV_186	11.29574	P-Firmicutes	G-Subdoligranulum uncl.	35.806873	0.000000	0.000000	
L	ASV_212	13.75428	P-Firmicutes	G-Streptococcus uncl.	32.167001	0.000000	0.000002	
L	ASV_214	11.33370	P-Firmicutes	G-Faecalibacterium uncl.	25.892199	0.000002	0.000034	
L	ASV_221	7.23754	P-Bacteroidetes	G-Alistipes uncl.	19.157470	0.000069	0.000752	
L	ASV_287	18.43047	P-Firmicutes	G-Ruminococcus uncl.	22.969979	0.000010	0.000131	
L	ASV_294	24.38929	P-Bacteroidetes	G-Bacteroides uncl.	35.349860	0.000000	0.000000	
L	ASV_351	5.90574	P-Firmicutes	G-Faecalibacterium uncl.	15.837075	0.000364	0.003287	
L	ASV_554	3.64345	P-Proteobacteria	G-Sutterella uncl.	12.652908	0.001788	0.013997	
L	ASV_801	9.08824	P-Firmicutes	F-Ruminococcaceae uncl.	12.575162	0.001859	0.014360	
L	ASV_10	80.22210	P-Bacteroidetes	S-Bacteroides-uniformis	26.595765	0.000002	0.000026	L2>L1,L3>L1,L3>L2
L	ASV_19	46.49237	P-Firmicutes	F-Lachnospiraceae uncl.	102.702938	0.000000	0.000000	
L	ASV_44	15.23001	P-Bacteroidetes	S-Bacteroides-faecis	57.325805	0.000000	0.000000	
L	ASV_51	74.22143	P-Bacteroidetes	G-Barnesiella uncl.	27.594981	0.000001	0.000017	
L	ASV_54	36.80121	P-Bacteroidetes	G-Bacteroides uncl.	26.063906	0.000002	0.000032	
L	ASV_61	15.53205	P-Bacteroidetes	G-Alistipes uncl.	10.419955	0.005462	0.036433	
L	ASV_75	24.85366	P-Proteobacteria	G-Klebsiella uncl.	16.283483	0.000291	0.002756	
L	ASV_78	27.93286	P-Firmicutes	G-Phascolarctobacterium uncl.	16.027265	0.000331	0.003035	
L	ASV_95	11.27460	P-Firmicutes	F-Ruminococcaceae uncl.	32.578431	0.000000	0.000002	

L	ASV_157	29.03295	P-Bacteroidetes	S-Bacteroides-clarus	14.372772	0.000757	0.006257	
L	ASV_159	28.21102	P-Firmicutes	F-Ruminococcaceae uncl.	31.247668	0.000000	0.000003	
L	ASV_199	17.47131	P-Proteobacteria	G-Parasutterella uncl.	57.038416	0.000000	0.000000	
L	ASV_267	6.69520	P-Firmicutes	G-Subdoligranulum uncl.	26.338832	0.000002	0.000029	
L	ASV_511	9.79593	P-Firmicutes	G-Subdoligranulum uncl.	18.797064	0.000083	0.000884	
L	ASV_965	3.10426	P-Proteobacteria	G-Escherichia/Shigella uncl.	11.278842	0.003555	0.026414	
L	ASV_1278	2.58845	P-Firmicutes	S-Veillonella-parvula	10.071882	0.006500	0.041630	

Appendix Table 7: Analysis of differential abundance with respect to Montreal Classification.

A: Age of onset in CD, B: Behaviour in CD, E: Extent in CD, L: Location

ASV	Phylum	Organism	Mean	Stat	*P* value	P_{FDR}	Direction
ASV_126	P-Firmicutes	F-Lachnospiraceae uncl.	16.19969	403.50851	0.00000	0.00000	Ex>Yes>No
ASV_36	P-Bacteroidetes	S-Bacteroides_dorei	41.93582	15.84767	0.00036	0.02550	Ex>No>Yes
ASV_73	P-Bacteroidetes	G-Bacteroides uncl.	20.96816	603.33003	0.00000	0.00000	Ex>No>Yes
ASV_441	P-Proteobacteria	G-Parasutterella uncl.	3.67220	20.14265	0.00004	0.00347	Ex>No>Yes
ASV_557	P-Bacteroidetes	G-Bacteroides uncl.	4.68444	49.54323	0.00000	0.00000	Ex>No>Yes
ASV_56	P-Bacteroidetes	S-Bacteroides_ovatus	24.90248	104.78186	0.00000	0.00000	No>Ex>Yes
ASV_211	P-Proteobacteria	G-Citrobacter uncl.	9.40224	57.81672	0.00000	0.00000	No>Ex>Yes
ASV_227	P-Firmicutes	G-Anaerostipes uncl.	7.58258	221.59755	0.00000	0.00000	No>Ex>Yes
ASV_594	P-Firmicutes	F-Ruminococcaceae uncl.	4.71044	53.48079	0.00000	0.00000	No>Ex>Yes
ASV_188	P-Proteobacteria	G-Bilophila uncl.	14.06760	366.61668	0.00000	0.00000	No>Yes>Ex
ASV_288	P-Firmicutes	G-Faecalibacterium uncl.	7.35843	235.49710	0.00000	0.00000	No>Yes>Ex
ASV_55	P-Bacteroidetes	G-Bacteroides uncl.	32.60709	19.13993	0.00007	0.00529	Yes>Ex>No
ASV_373	P-Firmicutes	G-Faecalibacterium uncl.	5.59123	103.16126	0.00000	0.00000	Yes>Ex>No
ASV_66	P-Proteobacteria	G-Sutterella uncl.	30.18223	678.52946	0.00000	0.00000	Yes>No>Ex

Appendix Table 9: Differential abundance analysis for smoking behaviour

	Medication	ASV	Phylum	Organism	Mean	Stat	*P* value	P_{FDR}
CD	Thiopurine	ASV_60	P-Actinobacteria	S-Collinsella_aerofaciens	14.19672	43.47643	0.00000	0.00000
CD	Thiopurine	ASV_142	P-Actinobacteria	S-Collinsella_aerofaciens	21.58774	8.93692	0.00279	0.03281
CD	Thiopurine	ASV_23	P-Bacteroidetes	G-Prevotella uncl.	83.57133	9.97438	0.00159	0.02026
CD	Thiopurine	ASV_27	P-Bacteroidetes	G-Bacteroides uncl.	62.36070	10.64210	0.00111	0.01475
CD	Thiopurine	ASV_34	P-Bacteroidetes	G-Parabacteroides uncl.	70.13668	8.14894	0.00431	0.04684
CD	Thiopurine	ASV_35	P-Bacteroidetes	S-Parabacteroides_distasonis	40.38527	26.52610	0.00000	0.00001
CD	Thiopurine	ASV_36	P-Bacteroidetes	S-Bacteroides_dorei	61.06020	44.55756	0.00000	0.00000
CD	Thiopurine	ASV_40	P-Bacteroidetes	G-Paraprevotella uncl.	70.67344	16.80038	0.00004	0.00070
CD	Thiopurine	ASV_51	P-Bacteroidetes	G-Barnesiella uncl.	74.22143	30.86448	0.00000	0.00000
CD	Thiopurine	ASV_67	P-Bacteroidetes	S-Bacteroides_thetaiotaomicron	10.35938	11.37024	0.00075	0.01043
CD	Thiopurine	ASV_259	P-Bacteroidetes	S-Bacteroides_finegoldii	32.06328	22.33450	0.00000	0.00005
CD	Thiopurine	ASV_473	P-Bacteroidetes	G-Bacteroides uncl.	7.25661	27.90642	0.00000	0.00000
CD	Thiopurine	ASV_527	P-Bacteroidetes	G-Prevotella uncl.	12.64440	32.65729	0.00000	0.00000
CD	Thiopurine	ASV_8	P-Firmicutes	G-Dialister uncl.	101.33687	81.22337	0.00000	0.00000
CD	Thiopurine	ASV_133	P-Firmicutes	S-Dialister_succinatiphilus	53.75416	43.47817	0.00000	0.00000
CD	Thiopurine	ASV_145	P-Firmicutes	S-Anaerostipes_hadrus	14.94033	40.49459	0.00000	0.00000
CD	Thiopurine	ASV_212	P-Firmicutes	G-Streptococcus uncl.	13.75428	33.25737	0.00000	0.00000
CD	Thiopurine	ASV_243	P-Firmicutes	S-Roseburia_hominis	11.97483	35.41120	0.00000	0.00000
CD	Thiopurine	ASV_321	P-Firmicutes	O-Clostridiales uncl.	2.99449	8.25610	0.00406	0.04498
CD	Thiopurine	ASV_354	P-Firmicutes	G-Blautia uncl.	6.50442	26.77561	0.00000	0.00001
CD	Thiopurine	ASV_2822	P-Firmicutes	G-Faecalibacterium uncl.	3.75788	13.57362	0.00023	0.00345
CD	Thiopurine	ASV_13	P-Proteobacteria	G-Escherichia/Shigella uncl.	87.75980	22.04538	0.00000	0.00006
CD	Thiopurine	ASV_31	P-Proteobacteria	G-Escherichia/Shigella uncl.	30.83048	61.27177	0.00000	0.00000
CD	Thiopurine	ASV_75	P-Proteobacteria	G-Klebsiella uncl.	24.85366	13.23324	0.00028	0.00404
CD	Thiopurine	ASV_282	P-Proteobacteria	G-Escherichia/Shigella uncl.	35.33819	32.24550	0.00000	0.00000
CD	Thiopurine	ASV_422	P-Proteobacteria	S-Escherichia/Shigella_coli	17.42229	15.98763	0.00006	0.00101
CD	Thiopurine	ASV_1309	P-Proteobacteria	G-Sutterella uncl.	10.82900	9.62824	0.00192	0.02295

CD	no Thiopurine	ASV_5	P-Bacteroidetes	S-Bacteroides_dorei	319.34058	16.90676	0.00004	0.00068
CD	no Thiopurine	ASV_14	P-Bacteroidetes	G-Alistipes uncl.	131.39524	16.56329	0.00005	0.00077
CD	no Thiopurine	ASV_83	P-Bacteroidetes	G-Bacteroides uncl.	20.05108	10.65147	0.00110	0.01475
CD	no Thiopurine	ASV_108	P-Bacteroidetes	G-Bacteroides uncl.	47.33334	9.67689	0.00187	0.02282
CD	no Thiopurine	ASV_114	P-Bacteroidetes	S-Bacteroides_fragilis	67.15736	72.68424	0.00000	0.00000
CD	no Thiopurine	ASV_119	P-Bacteroidetes	S-Bacteroides_cellulosilyticus	15.41636	14.60434	0.00013	0.00205
CD	no Thiopurine	ASV_131	P-Bacteroidetes	G-Bacteroides uncl.	110.42208	61.23881	0.00000	0.00000
CD	no Thiopurine	ASV_160	P-Bacteroidetes	G-Bacteroides uncl.	3.65863	10.27813	0.00135	0.01756
CD	no Thiopurine	ASV_200	P-Bacteroidetes	G-Bacteroides uncl.	43.11352	46.81482	0.00000	0.00000
CD	no Thiopurine	ASV_291	P-Bacteroidetes	G-Bacteroides uncl.	35.92474	45.56114	0.00000	0.00000
CD	no Thiopurine	ASV_340	P-Bacteroidetes	G-Parabacteroides uncl.	10.13770	34.99490	0.00000	0.00000
CD	no Thiopurine	ASV_495	P-Bacteroidetes	G-Bacteroides uncl.	2.30933	8.06006	0.00453	0.04830
CD	no Thiopurine	ASV_19	P-Firmicutes	F-Lachnospiraceae uncl.	46.49237	100.24616	0.00000	0.00000
CD	no Thiopurine	ASV_29	P-Firmicutes	S-Dialister_invisus	79.28204	105.46554	0.00000	0.00000
CD	no Thiopurine	ASV_96	P-Firmicutes	F-Ruminococcaceae uncl.	23.38052	55.91235	0.00000	0.00000
CD	no Thiopurine	ASV_140	P-Firmicutes	G-Phascolarctobacterium uncl.	23.93240	35.16024	0.00000	0.00000
CD	no Thiopurine	ASV_226	P-Firmicutes	G-Ruminococcus2 uncl.	15.26128	54.71519	0.00000	0.00000
CD	no Thiopurine	ASV_265	P-Firmicutes	S-Dorea_longicatena	12.44200	25.30188	0.00000	0.00001
CD	no Thiopurine	ASV_275	P-Firmicutes	G-Clostridium_IV uncl.	22.38124	19.17958	0.00001	0.00024
CD	no Thiopurine	ASV_313	P-Firmicutes	G-Faecalibacterium uncl.	3.43980	8.67663	0.00322	0.03710
CD	no Thiopurine	ASV_423	P-Firmicutes	F-Lachnospiraceae uncl.	4.04494	11.68511	0.00063	0.00902
CD	no Thiopurine	ASV_448	P-Firmicutes	G-Subdoligranulum uncl.	11.91290	16.92308	0.00004	0.00068
CD	no Thiopurine	ASV_583	P-Firmicutes	G-Ruminococcus2 uncl.	4.50533	23.74547	0.00000	0.00002
CD	no Thiopurine	ASV_801	P-Firmicutes	F-Ruminococcaceae uncl.	9.08824	8.34532	0.00387	0.04365
CD	no Thiopurine	ASV_30	P-Proteobacteria	F-Enterobacteriaceae uncl.	78.47085	17.36301	0.00003	0.00058
CD	no Thiopurine	ASV_173	P-Proteobacteria	G-Citrobacter uncl.	25.56731	17.00196	0.00004	0.00068
CD	no Thiopurine	ASV_211	P-Proteobacteria	G-Citrobacter uncl.	2.53080	9.84499	0.00170	0.02127
CD	no Thiopurine	ASV_219	P-Proteobacteria	G-Enterobacter uncl.	56.21588	17.56571	0.00003	0.00054
CD	anti-TNF	ASV_59	P-Actinobacteria	S-Collinsella_aerofaciens	12.31894	15.54724	0.00008	0.00146
CD	anti-TNF	ASV_60	P-Actinobacteria	S-Collinsella_aerofaciens	14.19672	43.42412	0.00000	0.00000
CD	anti-TNF	ASV_5	P-Bacteroidetes	S-Bacteroides_dorei	319.34058	230.12230	0.00000	0.00000

CD	anti-TNF	ASV_14	P-Bacteroidetes	G-Alistipes uncl.	131.39524	22.54891	0.00000	0.00005
CD	anti-TNF	ASV_36	P-Bacteroidetes	S-Bacteroides_dorei	61.06020	36.55790	0.00000	0.00000
CD	anti-TNF	ASV_42	P-Bacteroidetes	G-Bacteroides uncl.	88.30275	19.90299	0.00001	0.00016
CD	anti-TNF	ASV_65	P-Bacteroidetes	G-Parabacteroides uncl.	10.89093	31.52635	0.00000	0.00000
CD	anti-TNF	ASV_67	P-Bacteroidetes	S-Bacteroides_thetaiotaomicron	10.35938	19.32795	0.00001	0.00021
CD	anti-TNF	ASV_119	P-Bacteroidetes	S-Bacteroides_cellulosilyticus	15.41636	59.00576	0.00000	0.00000
CD	anti-TNF	ASV_137	P-Bacteroidetes	S-Alistipes_finegoldii	22.66353	10.84866	0.00099	0.01281
CD	anti-TNF	ASV_281	P-Bacteroidetes	S-Alistipes_massiliensis	24.73205	7.98390	0.00472	0.04937
CD	anti-TNF	ASV_292	P-Bacteroidetes	S-Bacteroides_thetaiotaomicron	10.94616	40.00895	0.00000	0.00000
CD	anti-TNF	ASV_473	P-Bacteroidetes	G-Bacteroides uncl.	7.25661	27.23242	0.00000	0.00001
CD	anti-TNF	ASV_25	P-Firmicutes	G-Subdoligranulum uncl.	57.17168	123.88154	0.00000	0.00000
CD	anti-TNF	ASV_78	P-Firmicutes	G-Phascolarctobacterium uncl.	27.93286	22.13463	0.00000	0.00006
CD	anti-TNF	ASV_209	P-Firmicutes	G-Subdoligranulum uncl.	11.79678	9.04843	0.00263	0.02980
CD	anti-TNF	ASV_267	P-Firmicutes	G-Subdoligranulum uncl.	6.69520	26.77125	0.00000	0.00001
CD	anti-TNF	ASV_511	P-Firmicutes	G-Subdoligranulum uncl.	9.79593	9.23148	0.00238	0.02753
CD	anti-TNF	ASV_847	P-Firmicutes	G-Catenibacterium uncl.	8.79736	9.65112	0.00189	0.02394
CD	anti-TNF	ASV_1278	P-Firmicutes	S-Veillonella_parvula	2.58845	9.24886	0.00236	0.02753
CD	anti-TNF	ASV_31	P-Proteobacteria	G-Escherichia/Shigella uncl.	30.83048	61.56989	0.00000	0.00000
CD	anti-TNF	ASV_53	P-Proteobacteria	G-Parasutterella uncl.	83.04174	30.21357	0.00000	0.00000
CD	anti-TNF	ASV_66	P-Proteobacteria	G-Sutterella uncl.	36.81866	19.70672	0.00001	0.00018
CD	anti-TNF	ASV_75	P-Proteobacteria	G-Klebsiella uncl.	24.85366	23.28482	0.00000	0.00003
CD	anti-TNF	ASV_101	P-Proteobacteria	G-Klebsiella uncl.	19.13745	14.67692	0.00013	0.00224
CD	anti-TNF	ASV_1309	P-Proteobacteria	G-Sutterella uncl.	10.82900	12.61136	0.00038	0.00632
CD	no anti-TNF	ASV_142	P-Actinobacteria	S-Collinsella_aerofaciens	21.58774	10.87949	0.00097	0.01281
CD	no anti-TNF	ASV_23	P-Bacteroidetes	G-Prevotella uncl.	83.57133	67.84398	0.00000	0.00000
CD	no anti-TNF	ASV_40	P-Bacteroidetes	G-Paraprevotella uncl.	70.67344	8.93665	0.00280	0.03103
CD	no anti-TNF	ASV_94	P-Bacteroidetes	S-Bacteroides_vulgatus	26.99081	53.70900	0.00000	0.00000
CD	no anti-TNF	ASV_108	P-Bacteroidetes	G-Bacteroides uncl.	47.33334	8.64126	0.00329	0.03576
CD	no anti-TNF	ASV_200	P-Bacteroidetes	G-Bacteroides uncl.	43.11352	29.39999	0.00000	0.00000
CD	no anti-TNF	ASV_255	P-Bacteroidetes	G-Prevotella uncl.	34.57826	11.98712	0.00054	0.00809
CD	no anti-TNF	ASV_363	P-Bacteroidetes	G-Bacteroides uncl.	26.54383	11.54601	0.00068	0.00972

CD	no anti-TNF	ASV_495	P-Bacteroidetes	G-Bacteroides uncl.	2.30933	8.39950	0.00375	0.04003
CD	no anti-TNF	ASV_575	P-Bacteroidetes	G-Parabacteroides uncl.	8.91137	11.62720	0.00065	0.00956
CD	no anti-TNF	ASV_633	P-Bacteroidetes	G-Bacteroides uncl.	9.39460	21.64990	0.00000	0.00007
CD	no anti-TNF	ASV_1009	P-Bacteroidetes	G-Prevotella uncl.	12.80145	37.16526	0.00000	0.00000
CD	no anti-TNF	ASV_29	P-Firmicutes	S-Dialister_invisus	79.28204	73.50062	0.00000	0.00000
CD	no anti-TNF	ASV_70	P-Firmicutes	S-Lactobacillus_gasseri	22.34708	12.91553	0.00033	0.00554
CD	no anti-TNF	ASV_86	P-Firmicutes	S-Dialister_invisus	55.92837	25.57107	0.00000	0.00001
CD	no anti-TNF	ASV_89	P-Firmicutes	G-Turicibacter uncl.	24.01321	66.77813	0.00000	0.00000
CD	no anti-TNF	ASV_106	P-Firmicutes	G-Ruminococcus uncl.	28.60468	12.06005	0.00052	0.00801
CD	no anti-TNF	ASV_133	P-Firmicutes	S-Dialister_succinatiphilus	53.75416	11.33493	0.00076	0.01061
CD	no anti-TNF	ASV_145	P-Firmicutes	S-Anaerostipes_hadrus	14.94033	40.72723	0.00000	0.00000
CD	no anti-TNF	ASV_167	P-Firmicutes	S-Roseburia_faecis	10.08626	11.10233	0.00086	0.01173
CD	no anti-TNF	ASV_224	P-Firmicutes	G-Faecalibacterium uncl.	8.50762	21.02371	0.00000	0.00009
CD	no anti-TNF	ASV_226	P-Firmicutes	G-Ruminococcus2 uncl.	15.26128	50.42096	0.00000	0.00000
CD	no anti-TNF	ASV_228	P-Firmicutes	S-Faecalibacterium_prausnitzii	14.80294	46.87617	0.00000	0.00000
CD	no anti-TNF	ASV_288	P-Firmicutes	G-Faecalibacterium uncl.	6.32319	12.09015	0.00051	0.00801
CD	no anti-TNF	ASV_737	P-Firmicutes	G-Clostridium_XlVb uncl.	5.10448	9.43122	0.00213	0.02637
CD	no anti-TNF	ASV_211	P-Proteobacteria	G-Citrobacter uncl.	2.53080	9.27741	0.00232	0.02753
CD	5-ASA	ASV_12	P-Bacteroidetes	G-Bacteroides uncl.	61.27566	9.80708	0.00174	0.02202
CD	5-ASA	ASV_17	P-Bacteroidetes	G-Bacteroides uncl.	29.72181	85.94572	0.00000	0.00000
CD	5-ASA	ASV_18	P-Bacteroidetes	G-Bacteroides uncl.	34.26563	20.02466	0.00001	0.00015
CD	5-ASA	ASV_51	P-Bacteroidetes	G-Barnesiella uncl.	74.22143	9.69579	0.00185	0.02266
CD	5-ASA	ASV_54	P-Bacteroidetes	G-Bacteroides uncl.	36.80121	19.00528	0.00001	0.00023
CD	5-ASA	ASV_94	P-Bacteroidetes	S-Bacteroides_vulgatus	26.99081	54.05942	0.00000	0.00000
CD	5-ASA	ASV_108	P-Bacteroidetes	G-Bacteroides uncl.	47.33334	24.70527	0.00000	0.00002
CD	5-ASA	ASV_200	P-Bacteroidetes	G-Bacteroides uncl.	43.11352	38.94290	0.00000	0.00000
CD	5-ASA	ASV_259	P-Bacteroidetes	S-Bacteroides_finegoldii	32.06328	12.48352	0.00041	0.00609
CD	5-ASA	ASV_291	P-Bacteroidetes	G-Bacteroides uncl.	35.92474	8.31351	0.00394	0.04125
CD	5-ASA	ASV_292	P-Bacteroidetes	S-Bacteroides_thetaiotaomicron	10.94616	39.48413	0.00000	0.00000
CD	5-ASA	ASV_348	P-Bacteroidetes	S-Odoribacter_splanchnicus	7.18832	20.09802	0.00001	0.00015
CD	5-ASA	ASV_603	P-Bacteroidetes	G-Barnesiella uncl.	8.36375	27.53053	0.00000	0.00000

CD	5-ASA	ASV_29	P-Firmicutes	S-Dialister_invisus	79.28204	44.09620	0.00000	0.00000
CD	5-ASA	ASV_78	P-Firmicutes	G-Phascolarctobacterium uncl.	27.93286	15.41380	0.00009	0.00142
CD	5-ASA	ASV_86	P-Firmicutes	S-Dialister_invisus	55.92837	21.68615	0.00000	0.00007
CD	5-ASA	ASV_89	P-Firmicutes	G-Turicibacter uncl.	24.01321	72.89843	0.00000	0.00000
CD	5-ASA	ASV_145	P-Firmicutes	S-Anaerostipes_hadrus	14.94033	41.22169	0.00000	0.00000
CD	5-ASA	ASV_167	P-Firmicutes	S-Roseburia_faecis	10.08626	9.14058	0.00250	0.02898
CD	5-ASA	ASV_224	P-Firmicutes	G-Faecalibacterium uncl.	8.50762	23.02151	0.00000	0.00004
CD	5-ASA	ASV_226	P-Firmicutes	G-Ruminococcus2 uncl.	15.26128	50.45938	0.00000	0.00000
CD	5-ASA	ASV_243	P-Firmicutes	S-Roseburia_hominis	11.97483	37.54946	0.00000	0.00000
CD	5-ASA	ASV_287	P-Firmicutes	G-Ruminococcus uncl.	18.43047	12.28335	0.00046	0.00662
CD	5-ASA	ASV_313	P-Firmicutes	G-Faecalibacterium uncl.	3.43980	8.70595	0.00317	0.03444
CD	5-ASA	ASV_369	P-Firmicutes	F-Ruminococcaceae uncl.	8.33066	23.13216	0.00000	0.00004
CD	5-ASA	ASV_480	P-Firmicutes	G-Roseburia uncl.	9.14567	8.76194	0.00308	0.03400
CD	5-ASA	ASV_583	P-Firmicutes	G-Ruminococcus2 uncl.	4.50533	17.05591	0.00004	0.00063
CD	5-ASA	ASV_120	P-Proteobacteria	G-Parasutterella uncl.	15.22814	10.45542	0.00122	0.01616
CD	5-ASA	ASV_554	P-Proteobacteria	G-Sutterella uncl.	3.64345	11.95569	0.00054	0.00770
CD	no 5-ASA	ASV_59	P-Actinobacteria	S-Collinsella_aerofaciens	12.31894	13.38733	0.00025	0.00385
CD	no 5-ASA	ASV_60	P-Actinobacteria	S-Collinsella_aerofaciens	14.19672	45.26667	0.00000	0.00000
CD	no 5-ASA	ASV_142	P-Actinobacteria	S-Collinsella_aerofaciens	21.58774	24.52526	0.00000	0.00002
CD	no 5-ASA	ASV_16	P-Bacteroidetes	S-Alistipes_shahii	42.36141	101.98612	0.00000	0.00000
CD	no 5-ASA	ASV_42	P-Bacteroidetes	G-Bacteroides uncl.	88.30275	43.21321	0.00000	0.00000
CD	no 5-ASA	ASV_56	P-Bacteroidetes	S-Bacteroides_ovatus	27.20263	43.16877	0.00000	0.00000
CD	no 5-ASA	ASV_67	P-Bacteroidetes	S-Bacteroides_thetaiotaomicron	10.35938	19.74511	0.00001	0.00016
CD	no 5-ASA	ASV_131	P-Bacteroidetes	G-Bacteroides uncl.	110.42208	32.76828	0.00000	0.00000
CD	no 5-ASA	ASV_363	P-Bacteroidetes	G-Bacteroides uncl.	26.54383	64.07030	0.00000	0.00000
CD	no 5-ASA	ASV_517	P-Bacteroidetes	G-Alistipes uncl.	5.59885	9.67936	0.00186	0.02266
CD	no 5-ASA	ASV_95	P-Firmicutes	F-Ruminococcaceae uncl.	11.27460	14.49375	0.00014	0.00225
CD	no 5-ASA	ASV_133	P-Firmicutes	S-Dialister_succinatiphilus	53.75416	8.12119	0.00438	0.04433
CD	no 5-ASA	ASV_159	P-Firmicutes	F-Ruminococcaceae uncl.	28.21102	20.03541	0.00001	0.00015
CD	no 5-ASA	ASV_164	P-Firmicutes	G-Anaerotruncus uncl.	9.48149	9.12156	0.00253	0.02898
CD	no 5-ASA	ASV_207	P-Firmicutes	G-Oscillibacter uncl.	12.00106	8.15735	0.00429	0.04420

CD	no 5-ASA	ASV_288	P-Firmicutes	G-Faecalibacterium uncl.	6.32319	14.07769	0.00018	0.00273
CD	no 5-ASA	ASV_413	P-Firmicutes	F-Ruminococcaceae uncl.	5.27628	8.62680	0.00331	0.03533
CD	no 5-ASA	ASV_493	P-Firmicutes	G-Streptococcus uncl.	16.54697	37.05840	0.00000	0.00000
CD	no 5-ASA	ASV_584	P-Firmicutes	S-Dialister_invisus	7.94324	8.82445	0.00297	0.03346
CD	no 5-ASA	ASV_737	P-Firmicutes	G-Clostridium_XlVb uncl.	5.10448	10.27725	0.00135	0.01742
CD	no 5-ASA	ASV_6	P-Proteobacteria	G-Escherichia/Shigella uncl.	205.74941	110.60941	0.00000	0.00000
CD	no 5-ASA	ASV_13	P-Proteobacteria	G-Escherichia/Shigella uncl.	87.75980	19.84251	0.00001	0.00016
CD	no 5-ASA	ASV_30	P-Proteobacteria	F-Enterobacteriaceae uncl.	78.47085	7.82966	0.00514	0.04960
CD	no 5-ASA	ASV_53	P-Proteobacteria	G-Parasutterella uncl.	83.04174	41.15830	0.00000	0.00000
CD	no 5-ASA	ASV_69	P-Proteobacteria	G-Parasutterella uncl.	14.30750	16.72852	0.00004	0.00073
CD	no 5-ASA	ASV_75	P-Proteobacteria	G-Klebsiella uncl.	24.85366	10.90598	0.00096	0.01295
CD	no 5-ASA	ASV_101	P-Proteobacteria	G-Klebsiella uncl.	19.13745	7.88384	0.00499	0.04891
CD	no 5-ASA	ASV_125	P-Proteobacteria	S-Escherichia/Shigella_coli	14.82968	36.78960	0.00000	0.00000
CD	no 5-ASA	ASV_219	P-Proteobacteria	G-Enterobacter uncl.	56.21588	7.93355	0.00485	0.04837
CD	no 5-ASA	ASV_266	P-Proteobacteria	G-Bilophila uncl.	8.89045	9.44655	0.00212	0.02522
CD	no 5-ASA	ASV_282	P-Proteobacteria	G-Escherichia/Shigella uncl.	35.33819	42.48093	0.00000	0.00000
CD	no 5-ASA	ASV_422	P-Proteobacteria	S-Escherichia/Shigella_coli	17.42229	21.46240	0.00000	0.00008
CD	no 5-ASA	ASV_20	P-Verrucomicrobia	S-Akkermansia_muciniphila	62.05206	27.74975	0.00000	0.00000
CD	no 5-ASA	ASV_446	P-Verrucomicrobia	G-Akkermansia uncl.	17.26252	11.88280	0.00057	0.00783
UC	Thiopurine	ASV_7	P-Bacteroidetes	S-Bacteroides_massiliensis	93.75372	16.30049	0.00005	0.00200
UC	Thiopurine	ASV_11	P-Bacteroidetes	G-Bacteroides uncl.	116.03086	23.88769	0.00000	0.00005
UC	Thiopurine	ASV_23	P-Bacteroidetes	G-Prevotella uncl.	55.18226	15.20973	0.00010	0.00338
UC	Thiopurine	ASV_61	P-Bacteroidetes	G-Alistipes uncl.	52.67899	16.35787	0.00005	0.00200
UC	Thiopurine	ASV_63	P-Bacteroidetes	S-Parabacteroides_distasonis	29.69888	187.87032	0.00000	0.00000
UC	Thiopurine	ASV_65	P-Bacteroidetes	G-Parabacteroides uncl.	38.24075	12.16267	0.00049	0.01414
UC	Thiopurine	ASV_99	P-Bacteroidetes	S-Alistipes_indistinctus	13.72506	9.85372	0.00169	0.04187
UC	Thiopurine	ASV_111	P-Bacteroidetes	G-Bacteroides uncl.	25.21531	19.06920	0.00001	0.00056
UC	Thiopurine	ASV_107	P-Firmicutes	G-Faecalibacterium uncl.	30.50817	197.01912	0.00000	0.00000
UC	Thiopurine	ASV_241	P-Firmicutes	G-Faecalibacterium uncl.	17.50134	10.63740	0.00111	0.02843
UC	Thiopurine	ASV_618	P-Firmicutes	G-Faecalibacterium uncl.	7.46440	34.64334	0.00000	0.00000
UC	Thiopurine	ASV_696	P-Firmicutes	G-Blautia uncl.	4.62685	13.09191	0.00030	0.00899

UC	Thiopurine	ASV_873	P-Firmicutes	G-Faecalibacterium uncl.	4.66522	14.67740	0.00013	0.00425
UC	Thiopurine	ASV_13	P-Proteobacteria	G-Escherichia/Shigella uncl.	40.96810	13.98356	0.00018	0.00586
UC	Thiopurine	ASV_24	P-Proteobacteria	G-Escherichia/Shigella uncl.	28.80730	19.89377	0.00001	0.00039
UC	no Thiopurine	ASV_231	K-Bacteria uncl.	K-Bacteria uncl.	12.11011	38.98587	0.00000	0.00000
UC	no Thiopurine	ASV_60	P-Actinobacteria	S-Collinsella_aerofaciens	26.12970	11.94348	0.00055	0.01524
UC	no Thiopurine	ASV_10	P-Bacteroidetes	S-Bacteroides_uniformis	149.19106	547.97880	0.00000	0.00000
UC	no Thiopurine	ASV_28	P-Bacteroidetes	G-Barnesiella uncl.	76.37325	32.41888	0.00000	0.00000
UC	no Thiopurine	ASV_129	P-Bacteroidetes	G-Prevotella uncl.	12.80860	37.86088	0.00000	0.00000
UC	no Thiopurine	ASV_131	P-Bacteroidetes	G-Bacteroides uncl.	7.92473	29.02149	0.00000	0.00000
UC	no Thiopurine	ASV_555	P-Bacteroidetes	S-Bacteroides_intestinalis	3.06671	10.83821	0.00099	0.02653
UC	no Thiopurine	ASV_571	P-Bacteroidetes	P-Bacteroidetes uncl. uncl.	10.73504	32.57117	0.00000	0.00000
UC	no Thiopurine	ASV_8	P-Firmicutes	G-Dialister uncl.	175.98817	26.26327	0.00000	0.00002
UC	no Thiopurine	ASV_93	P-Firmicutes	G-Phascolarctobacterium uncl.	35.03571	18.75535	0.00001	0.00062
UC	no Thiopurine	ASV_168	P-Firmicutes	F-Ruminococcaceae uncl.	15.66380	9.49117	0.00206	0.04918
UC	no Thiopurine	ASV_136	P-Proteobacteria	G-Escherichia/Shigella uncl.	10.46331	27.07010	0.00000	0.00001
UC	no Thiopurine	ASV_183	P-Proteobacteria	S-Escherichia/Shigella_coli	11.10272	29.90194	0.00000	0.00000
UC	anti-TNF	ASV_14	P-Bacteroidetes	G-Alistipes uncl.	38.50806	10.96346	0.00093	0.03109
UC	anti-TNF	ASV_23	P-Bacteroidetes	G-Prevotella uncl.	55.18226	29.84287	0.00000	0.00000
UC	anti-TNF	ASV_47	P-Bacteroidetes	G-Prevotella uncl.	37.43223	16.75986	0.00004	0.00173
UC	anti-TNF	ASV_63	P-Bacteroidetes	S-Parabacteroides_distasonis	29.69888	193.24090	0.00000	0.00000
UC	anti-TNF	ASV_99	P-Bacteroidetes	S-Alistipes_indistinctus	13.72506	132.76420	0.00000	0.00000
UC	anti-TNF	ASV_146	P-Bacteroidetes	G-Bacteroides uncl.	19.35994	11.41230	0.00073	0.02685
UC	anti-TNF	ASV_563	P-Bacteroidetes	G-Bacteroides uncl.	5.32369	19.31046	0.00001	0.00051
UC	anti-TNF	ASV_29	P-Firmicutes	S-Dialister_invisus	48.05438	45.06402	0.00000	0.00000
UC	anti-TNF	ASV_46	P-Firmicutes	F-Lachnospiraceae uncl.	30.68286	178.56040	0.00000	0.00000
UC	anti-TNF	ASV_57	P-Firmicutes	G-Megamonas uncl.	54.82247	52.31388	0.00000	0.00000
UC	anti-TNF	ASV_62	P-Firmicutes	S-Dialister_invisus	45.06592	55.77379	0.00000	0.00000
UC	anti-TNF	ASV_207	P-Firmicutes	G-Oscillibacter uncl.	8.62687	42.01588	0.00000	0.00000
UC	anti-TNF	ASV_220	P-Firmicutes	P-Firmicutes uncl. uncl.	15.44142	12.69837	0.00037	0.01418
UC	anti-TNF	ASV_326	P-Firmicutes	F-Ruminococcaceae uncl.	5.68406	18.82492	0.00001	0.00062
UC	anti-TNF	ASV_599	P-Firmicutes	G-Veillonella uncl.	2.62374	10.37155	0.00128	0.03767

UC	anti-TNF	ASV_1278	P-Firmicutes	S-Veillonella_parvula	2.36116	10.72403	0.00106	0.03243
UC	no anti-TNF	ASV_60	P-Actinobacteria	S-Collinsella_aerofaciens	26.12970	10.77956	0.00103	0.03243
UC	no anti-TNF	ASV_3	P-Bacteroidetes	S-Bacteroides_uniformis	235.37479	9.72249	0.00182	0.04962
UC	no anti-TNF	ASV_12	P-Bacteroidetes	G-Bacteroides uncl.	124.60847	452.46028	0.00000	0.00000
UC	no anti-TNF	ASV_26	P-Bacteroidetes	S-Alistipes_onderdonkii	32.11301	10.11399	0.00147	0.04165
UC	no anti-TNF	ASV_131	P-Bacteroidetes	G-Bacteroides uncl.	7.92473	35.48600	0.00000	0.00000
UC	no anti-TNF	ASV_555	P-Bacteroidetes	S-Bacteroides_intestinalis	3.06671	11.26305	0.00079	0.02771
UC	no anti-TNF	ASV_8	P-Firmicutes	G-Dialister uncl.	175.98817	92.60010	0.00000	0.00000
UC	no anti-TNF	ASV_85	P-Firmicutes	G-Dialister uncl.	18.92772	59.15271	0.00000	0.00000
UC	no anti-TNF	ASV_209	P-Firmicutes	G-Subdoligranulum uncl.	10.63879	42.91489	0.00000	0.00000
UC	no anti-TNF	ASV_359	P-Firmicutes	G-Dialister uncl.	10.66606	35.65416	0.00000	0.00000
UC	no anti-TNF	ASV_120	P-Proteobacteria	G-Parasutterella uncl.	10.26303	49.11269	0.00000	0.00000

Appendix Table 10: Differential abundance analysis based on medication. Cells highlighted in grey have ASVs abundant across both types of IBD for the same or different medications. Cells highlighted in grey are differentially abundant in the absence or presence of a medication in both CD and UC. Cells highlighted in yellow are differentially abundant in the absence or presence of more than one medication within the same cohort (UC or CD)

Index	ASV	Phylum	Organism	obs	Z	*P* value	P_{FDR}
Betweenness	ASV_648	P-Actinobacteria	S-Eggerthella_lenta	0.12383	7.24805	0.00000	0.00000
	ASV_187	P-Firmicutes	F-Ruminococcaceae uncl.	0.11085	6.65116	0.00000	0.00000
	ASV_64	P-Firmicutes	G-Oscillibacter uncl.	0.09139	5.24487	0.00000	0.00001
	ASV_312	P-Firmicutes	G-Veillonella uncl.	0.08315	4.94726	0.00000	0.00004
	ASV_321	P-Firmicutes	O-Clostridiales uncl.	0.07982	4.71878	0.00000	0.00009
	ASV_16	P-Bacteroidetes	S-Alistipes_shahii	0.06616	3.65589	0.00013	0.00839
	ASV_2	P-Bacteroidetes	S-Alistipes_putredinis	0.06539	3.60561	0.00016	0.00874
	ASV_198	P-Firmicutes	G-Oscillibacter uncl.	0.06235	3.43502	0.00030	0.01455
	ASV_130	P-Firmicutes	G-Faecalibacterium uncl.	0.05308	2.81110	0.00247	0.08820
	ASV_265	P-Firmicutes	S-Dorea_longicatena	0.05257	2.86373	0.00209	0.08227
	ASV_698	P-Firmicutes	F-Lachnospiraceae uncl.	0.05154	2.89276	0.00191	0.08227
	ASV_87	P-Firmicutes	S-Flavonifractor_plautii	0.05052	2.58883	0.00482	0.13517
	ASV_283	P-Firmicutes	G-Dorea uncl.	0.04987	2.70804	0.00338	0.11083
	ASV_960	P-Firmicutes	O-Clostridiales uncl.	0.04913	2.59007	0.00480	0.13517
	ASV_92	P-Firmicutes	G-Romboutsia uncl.	0.04857	2.44389	0.00726	0.17844
	ASV_774	P-Firmicutes	G-Ruminococcus uncl.	0.04827	2.54171	0.00552	0.14451
	ASV_38	P-Proteobacteria	S-Haemophilus_parainfluenzae	0.04363	2.21996	0.01321	0.30540
	ASV_89	P-Firmicutes	G-Turicibacter uncl.	0.03898	1.89014	0.02937	0.60748
	ASV_70	P-Firmicutes	S-Lactobacillus_gasseri	0.03873	1.89192	0.02925	0.60748
	ASV_30	P-Proteobacteria	F-Enterobacteriaceae uncl.	0.03573	1.73127	0.04170	0.72178
	ASV_1	P-Bacteroidetes	G-Bacteroides uncl.	0.03420	1.67585	0.04688	0.72178
Degree	ASV_64	P-Firmicutes	G-Oscillibacter uncl.	0.07143	5.31404	0.00000	0.00001
	ASV_648	P-Actinobacteria	S-Eggerthella_lenta	0.07143	5.30283	0.00000	0.00001
	ASV_87	P-Firmicutes	S-Flavonifractor_plautii	0.06888	4.94780	0.00000	0.00005
	ASV_321	P-Firmicutes	O-Clostridiales uncl.	0.06378	4.71998	0.00000	0.00012
	ASV_198	P-Firmicutes	G-Oscillibacter uncl.	0.05612	3.97299	0.00004	0.00279
	ASV_16	P-Bacteroidetes	S-Alistipes_shahii	0.04592	3.04532	0.00116	0.06525

	ASV_187	P-Firmicutes	F-Ruminococcaceae uncl.	0.04592	3.10525	0.00095	0.06226
	ASV_90	P-Firmicutes	G-Oscillibacter uncl.	0.04337	2.84258	0.00224	0.08802
	ASV_92	P-Firmicutes	G-Romboutsia uncl.	0.04337	2.80351	0.00253	0.09030
	ASV_541	P-Firmicutes	G-Oscillibacter uncl.	0.04337	2.84227	0.00224	0.08802
	ASV_960	P-Firmicutes	O-Clostridiales uncl.	0.04337	2.88532	0.00196	0.08802
	ASV_2	P-Bacteroidetes	S-Alistipes_putredinis	0.03827	2.39472	0.00832	0.20428
	ASV_24	P-Proteobacteria	G-Escherichia/Shigella uncl.	0.03827	2.33855	0.00968	0.21148
	ASV_38	P-Proteobacteria	S-Haemophilus_parainfluenzae	0.03827	2.42379	0.00768	0.20428
	ASV_45	P-Firmicutes	F-Lachnospiraceae uncl.	0.03827	2.40312	0.00813	0.20428
	ASV_296	P-Firmicutes	G-Oscillibacter uncl.	0.03827	2.33828	0.00969	0.21148
	ASV_635	P-Firmicutes	F-Ruminococcaceae uncl.	0.03827	2.48397	0.00650	0.20428
	ASV_698	P-Firmicutes	F-Lachnospiraceae uncl.	0.03827	2.44393	0.00726	0.20428
	ASV_30	P-Proteobacteria	F-Enterobacteriaceae uncl.	0.03571	2.15676	0.01551	0.31245
	ASV_88	P-Proteobacteria	G-Haemophilus uncl.	0.03571	2.11198	0.01734	0.31245
	ASV_547	P-Firmicutes	F-Ruminococcaceae uncl.	0.03571	2.13016	0.01658	0.31245
	ASV_759	P-Firmicutes	O-Clostridiales uncl.	0.03571	2.10857	0.01749	0.31245
	ASV_197	P-Firmicutes	G-Blautia uncl.	0.03316	1.95094	0.02553	0.41150
	ASV_312	P-Firmicutes	G-Veillonella uncl.	0.03316	1.97473	0.02415	0.41150
	ASV_387	P-Firmicutes	G-Romboutsia uncl.	0.03316	1.94021	0.02618	0.41150
	ASV_89	P-Firmicutes	G-Turicibacter uncl.	0.03061	1.67049	0.04741	0.60197
	ASV_95	P-Firmicutes	F-Ruminococcaceae uncl.	0.03061	1.71382	0.04328	0.60197
	ASV_206	P-Firmicutes	G-Blautia uncl.	0.03061	1.74454	0.04053	0.60197
	ASV_412	P-Firmicutes	G-Romboutsia uncl.	0.03061	1.66976	0.04748	0.60197
	ASV_519	P-Firmicutes	G-Blautia uncl.	0.03061	1.70973	0.04366	0.60197
	ASV_846	P-Actinobacteria	S-Eggerthella_lenta	0.03061	1.68313	0.04617	0.60197
Eigenvector-centrality	ASV_24	P-Proteobacteria	G-Escherichia/Shigella uncl.	1.00000	7.72436	0.00000	0.00000
	ASV_30	P-Proteobacteria	F-Enterobacteriaceae uncl.	0.99386	8.13304	0.00000	0.00000
	ASV_6	P-Proteobacteria	G-Escherichia/Shigella uncl.	0.89549	7.10792	0.00000	0.00000
	ASV_13	P-Proteobacteria	G-Escherichia/Shigella uncl.	0.85750	6.74362	0.00000	0.00000
	ASV_173	P-Proteobacteria	G-Citrobacter uncl.	0.74353	5.74151	0.00000	0.00000
	ASV_110	P-Proteobacteria	S-Escherichia/Shigella_coli	0.70618	5.26222	0.00000	0.00000

	ASV_118	P-Proteobacteria	G-Escherichia/Shigella uncl.	0.68691	5.31704	0.00000	0.00000
	ASV_181	P-Proteobacteria	G-Escherichia/Shigella uncl.	0.60090	4.70750	0.00000	0.00006
	ASV_74	P-Proteobacteria	G-Citrobacter uncl.	0.36782	2.86773	0.00207	0.09027
	ASV_31	P-Proteobacteria	G-Escherichia/Shigella uncl.	0.33805	2.42667	0.00762	0.27221
	ASV_121	P-Proteobacteria	G-Citrobacter uncl.	0.33248	2.35220	0.00933	0.30561
	ASV_125	P-Proteobacteria	S-Escherichia/Shigella_coli	0.31623	2.46316	0.00689	0.27062
	ASV_101	P-Proteobacteria	G-Klebsiella uncl.	0.30870	2.28864	0.01105	0.33406
	ASV_219	P-Proteobacteria	G-Enterobacter uncl.	0.24968	1.80194	0.03578	0.58635
	ASV_75	P-Proteobacteria	G-Klebsiella uncl.	0.24759	1.84495	0.03252	0.58635
PageRank	ASV_87	P-Firmicutes	S-Flavonifractor_plautii	0.01090	4.93413	0.00000	0.00007
	ASV_648	P-Actinobacteria	S-Eggerthella_lenta	0.01072	4.93428	0.00000	0.00007
	ASV_64	P-Firmicutes	G-Oscillibacter uncl.	0.01052	4.82590	0.00000	0.00007
	ASV_321	P-Firmicutes	O-Clostridiales uncl.	0.01049	4.87490	0.00000	0.00007
	ASV_960	P-Firmicutes	O-Clostridiales uncl.	0.00810	3.40325	0.00033	0.02617
	ASV_198	P-Firmicutes	G-Oscillibacter uncl.	0.00781	3.18779	0.00072	0.04695
	ASV_16	P-Bacteroidetes	S-Alistipes_shahii	0.00756	3.01898	0.00127	0.07120
	ASV_38	P-Proteobacteria	S-Haemophilus_parainfluenzae	0.00736	2.97369	0.00147	0.07227
	ASV_92	P-Firmicutes	G-Romboutsia uncl.	0.00734	2.89395	0.00190	0.08063
	ASV_187	P-Firmicutes	F-Ruminococcaceae uncl.	0.00721	2.87010	0.00205	0.08063
	ASV_88	P-Proteobacteria	G-Haemophilus uncl.	0.00682	2.55329	0.00534	0.19063
	ASV_45	P-Firmicutes	F-Lachnospiraceae uncl.	0.00648	2.41913	0.00778	0.25476
	ASV_90	P-Firmicutes	G-Oscillibacter uncl.	0.00638	2.34461	0.00952	0.27929
	ASV_2	P-Bacteroidetes	S-Alistipes_putredinis	0.00634	2.32826	0.00995	0.27929
	ASV_1	P-Bacteroidetes	G-Bacteroides uncl.	0.00627	2.29925	0.01075	0.28153
	ASV_541	P-Firmicutes	G-Oscillibacter uncl.	0.00626	2.26109	0.01188	0.29172
	ASV_24	P-Proteobacteria	G-Escherichia/Shigella uncl.	0.00625	2.23035	0.01286	0.29734
	ASV_296	P-Firmicutes	G-Oscillibacter uncl.	0.00600	2.05924	0.01974	0.37442
	ASV_698	P-Firmicutes	F-Lachnospiraceae uncl.	0.00599	2.12710	0.01671	0.36474
	ASV_759	P-Firmicutes	O-Clostridiales uncl.	0.00598	2.05360	0.02001	0.37442
	ASV_478	P-Firmicutes	G-Romboutsia uncl.	0.00582	2.05407	0.01998	0.37442
	ASV_368	P-Firmicutes	G-Blautia uncl.	0.00566	1.81749	0.03457	0.52255

	ASV_635	P-Firmicutes	F-Ruminococcaceae uncl.	0.00566	1.96285	0.02483	0.44359
	ASV_30	P-Proteobacteria	F-Enterobacteriaceae uncl.	0.00565	1.89533	0.02902	0.49593
	ASV_308	P-Firmicutes	G-Blautia uncl.	0.00557	1.85226	0.03199	0.52255
	ASV_130	P-Firmicutes	G-Faecalibacterium uncl.	0.00555	1.83314	0.03339	0.52255
	ASV_412	P-Firmicutes	G-Romboutsia uncl.	0.00545	1.72807	0.04199	0.58934
	ASV_95	P-Firmicutes	F-Ruminococcaceae uncl.	0.00541	1.75835	0.03934	0.57268
	ASV_387	P-Firmicutes	G-Romboutsia uncl.	0.00531	1.69236	0.04529	0.59699
	ASV_312	P-Firmicutes	G-Veillonella uncl.	0.00526	1.68940	0.04557	0.59699

Appendix Table 11: ASVs with significant ($P \leq 0.05$) Betweenness, Degree, Eigenvector-centrality and PageRank and their association to different anthropometric characteristics, as detected via indicators species analysis and analysis of differential abundances.

								Pfdr-Pairwise comparisons		
	Module	Module size	DF	Deviance	resid. Deviance	*P* value	P_{FDR}	Contr. vs CD	Contr. vs UC	CD vs. UC
IBD	1	71	2,191	43.14465	385.50012	4.27818×10^{-10}	2.13909×10^{-09}	0.000001	7.0217×10^{-08}	0.564310
	2	21	2,191	21.99344	548.51677	0.00002	0.00005	0.000231	0.001077	0.169022
	3	39	2,191	10.67231	549.60323	0.00481	0.00903	0.003887	0.266523	0.035488
	4	36	2,191	15.31021	597.84164	0.00047	0.00102	0.004401	0.002982	0.681132
	5	21	2,191	1.91023	614.29172	0.38477	0.44396	0.720780	0.526357	0.735706
	6	39	2,191	0.04117	448.47274	0.97962	0.97962	0.990931	0.990931	0.990931
	7	43	2,191	107.88079	688.12327	3.74959×10^{-24}	5.62439×10^{-23}	4.50590×10^{-08}	0.0000×10^{-23}	0.039773
	8	23	2,191	81.31984	688.31717	2.19594×10^{-18}	1.64695×10^{-17}	0.557495	0.0000×10^{-23}	1.3938×10^{-09}
	9	18	2,191	15.29184	266.47650	0.00048	0.00102	0.104495	0.000395	0.256325
	10	22	2,191	4.67754	449.65478	0.09645	0.12056	0.261581	0.131123	0.836605
	11	14	2,191	26.81159	318.12124	1.50639×10^{-06}	0.00001	0.000218	0.000040	0.639867
	12	8	2,191	7.50059	346.80341	0.02351	0.03918	0.909799	0.032680	0.063934
	13	9	2,191	6.55967	158.97946	0.03763	0.05132	0.066406	0.066406	0.596533
	15	9	2,191	1.73983	197.04399	0.41899	0.44892	0.474149	0.474149	0.745097
	19	7	2,191	6.58451	421.25314	0.03717	0.05132	0.095629	0.089471	0.845696
CD-CDAI	1	69	1,30	1.74830	65.31906	0.18609	0.39877			
	2	21	1,30	6.70546	61.90378	0.00961	0.07209			
	3	39	1,30	0.52493	75.46105	0.46875	0.63920			
	4	36	1,30	1.19968	93.38026	0.27338	0.51260			
	5	21	1,30	0.01157	130.05412	0.91433	0.91433			
	6	38	1,30	2.61028	46.62347	0.10617	0.26543			
	7	42	1,30	3.15501	76.12333	0.07569	0.26543			
	8	23	1,30	9.61382	103.56816	0.00193	0.02897			
	9	18	1,30	0.70941	57.60207	0.39964	0.63920			
	10	22	1,30	0.58298	77.03995	0.44515	0.63920			
	11	14	1,30	0.06594	52.84305	0.79734	0.91433			

	12	8	1,30	0.10083	38.19203	0.75084	0.91433			
	13	9	1,30	0.03315	30.83453	0.85553	0.91433			
	15	9	1,30	2.70826	26.76960	0.09983	0.26543			
	19	7	1,30	5.07305	66.79878	0.02430	0.12150			
UC-SCCAI	1	71	1,64	1.87819	109.51206	0.17054	0.42635			
	2	21	1,64	1.58903	156.61858	0.20746	0.44457			
	3	39	1,64	6.16333	170.10207	0.01304	0.15881			
	4	36	1,64	0.30372	184.17175	0.58156	0.73476			
	5	21	1,64	0.29379	208.11642	0.58780	0.73476			
	6	39	1,64	0.12963	178.15099	0.71882	0.82941			
	7	43	1,64	3.10750	227.84358	0.07793	0.23380			
	8	23	1,64	4.61104	233.10203	0.03177	0.15883			
	9	18	1,64	0.29653	88.62721	0.58607	0.73476			
	10	22	1,64	5.31233	134.12029	0.02118	0.15881			
	11	14	1,64	0.43183	109.99598	0.51109	0.73476			
	12	8	1,64	0.00968	140.31806	0.92161	0.92298			
	13	9	1,64	3.70239	58.06920	0.05433	0.20375			
	15	9	1,64	0.48104	65.14203	0.48795	0.73476			
	19	7	1,64	0.00935	145.35029	0.92298	0.92298			

Appendix Table 12: Analyses of module completeness with respect to IBD status and disease severity measures (CDAI in CD patients and SCCAI in UC patients) via binomial generalized linear models.

Highlighted cells have a *P* value and a P_{FDR} (adjusted via Benjamini-Hochberg procedure) less than 0.05

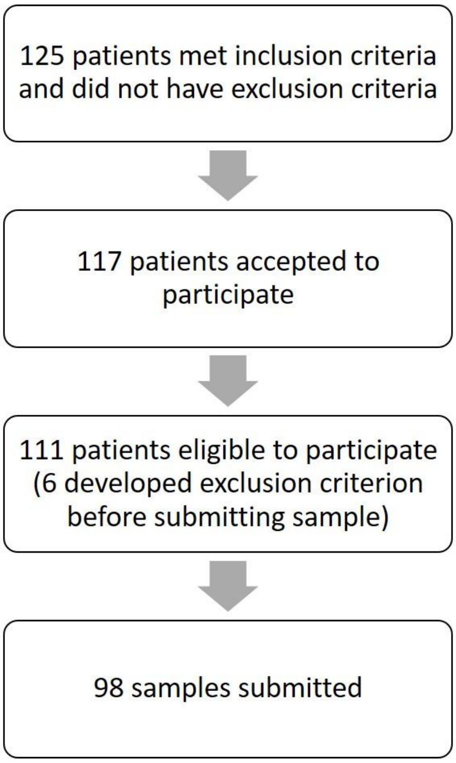

Appendix Figure 1: Numbers of individuals at each stage of the study

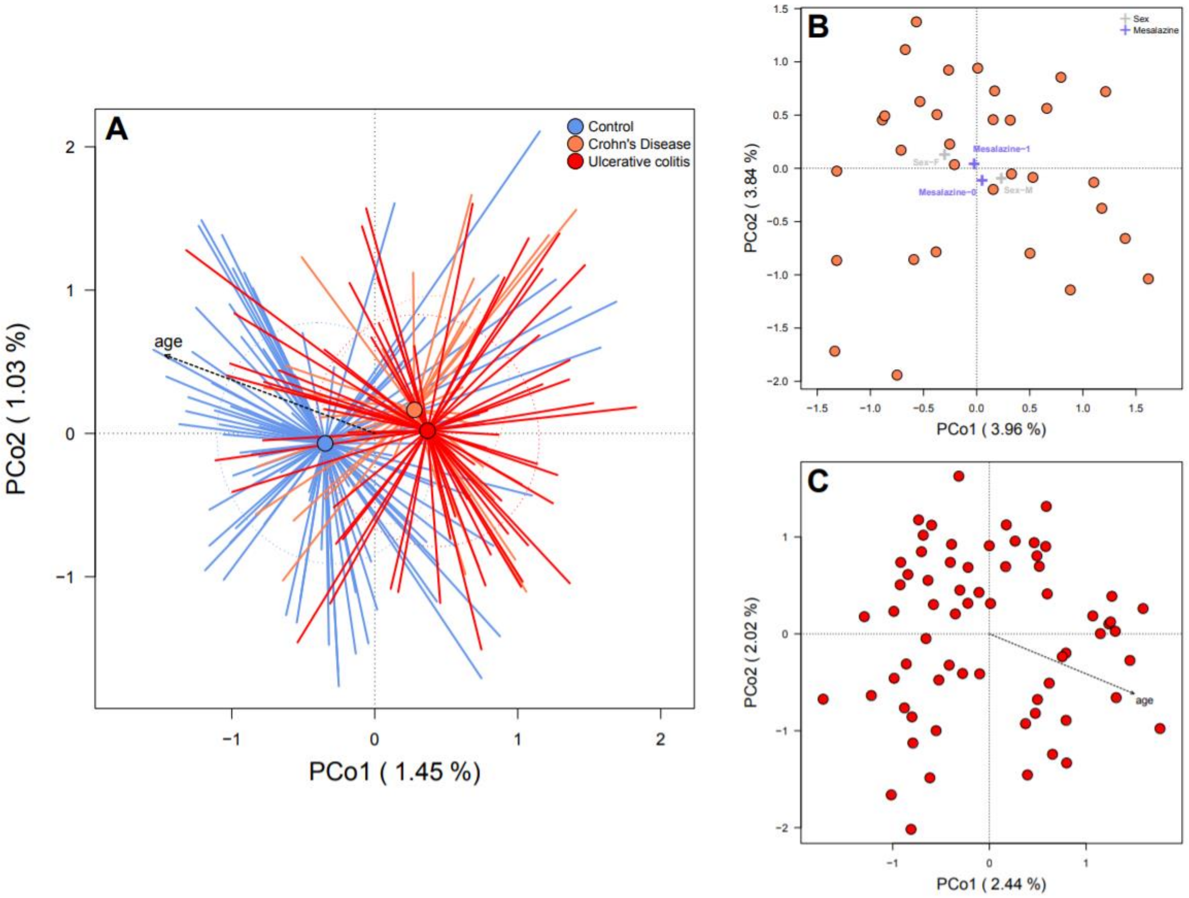

Appendix Figure 2 A) PCoA of Jaccard distance with respect to IBD status, including the significant correlations of community distance to age. B) PCoA to visualize community differences among only CD patients and (C) UC patients

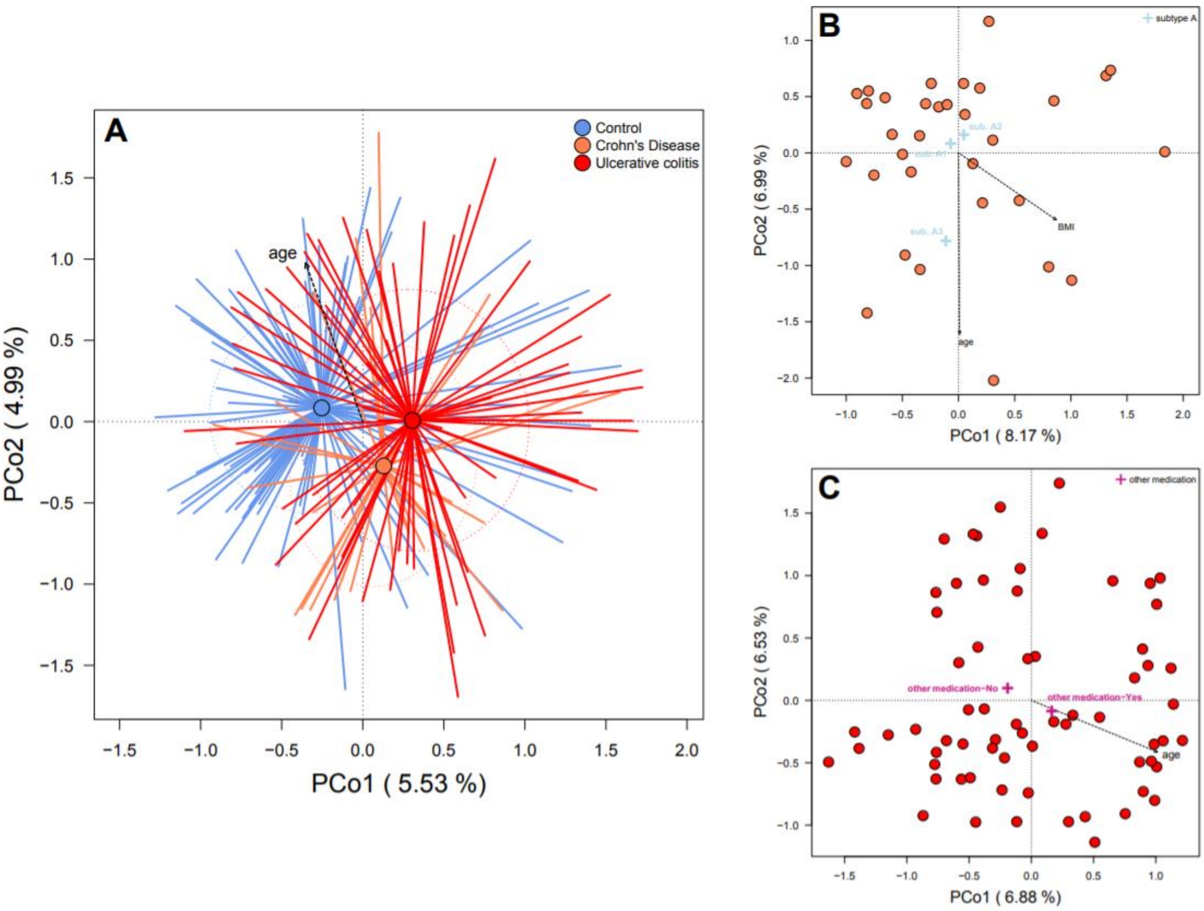

Appendix Figure 3: A) PCoA of the generalized UniFrac distance with respect to IBD status, including the significant correlations of community distance to age. B) PCoA to visualize community differences among only CD patients and (C) UC patients.

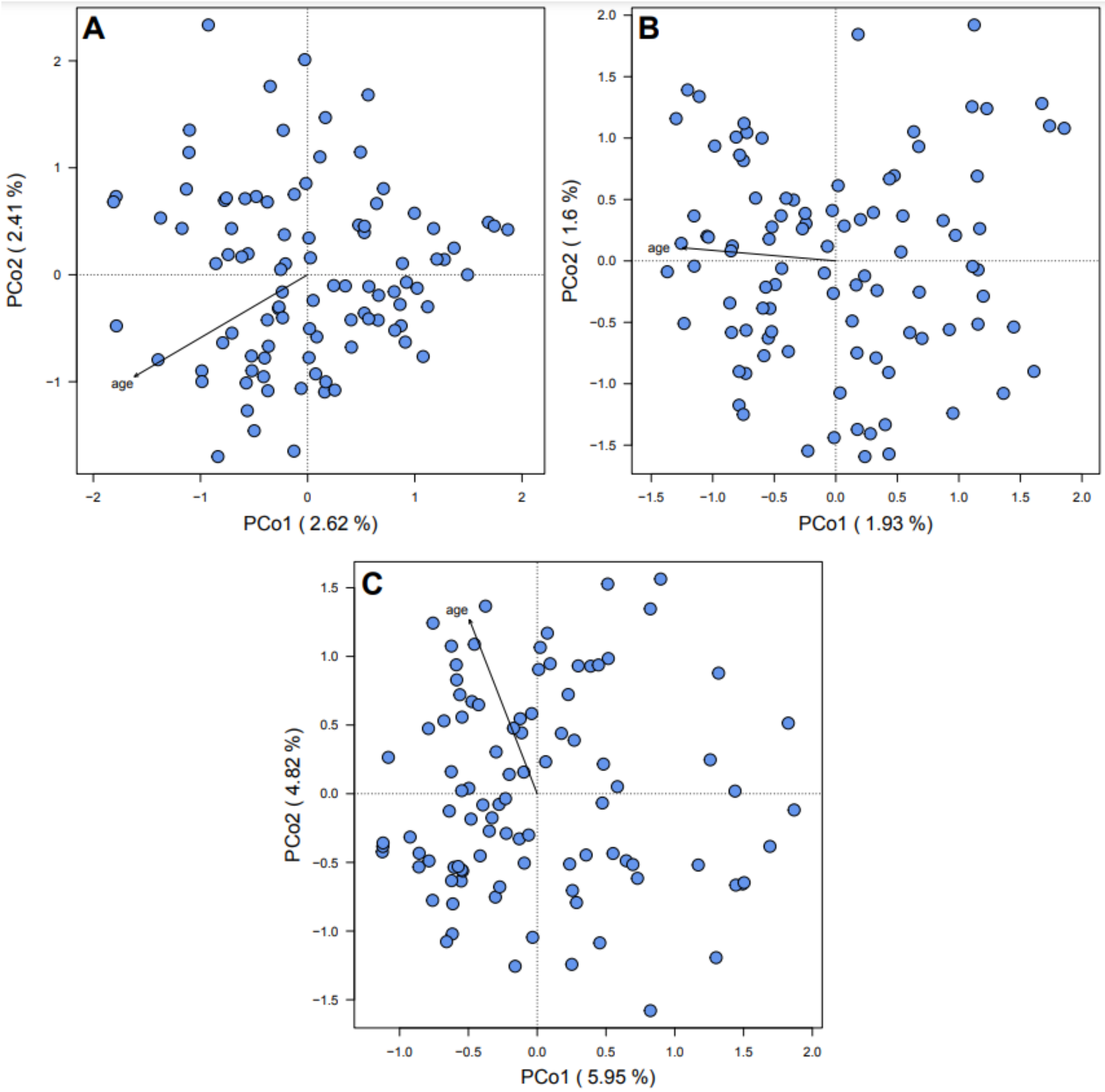

Appendix Figure 4: A) Principal Coordinate Analyses (PCoA) of Bray-Curtis dissimilarity, B) Jaccard distance, and generalized UniFrac distance among healthy control individuals. Arrows depict the significant correlations of community distances with age in healthy individuals (Bray-Curtis: $F_{1,94}$=1.10976, P=0.02670, Jaccard: $F_{1,94}$=1.08228, P=0.00430,gUF: $F_{1,94}$=1.30822, P=0.01860).

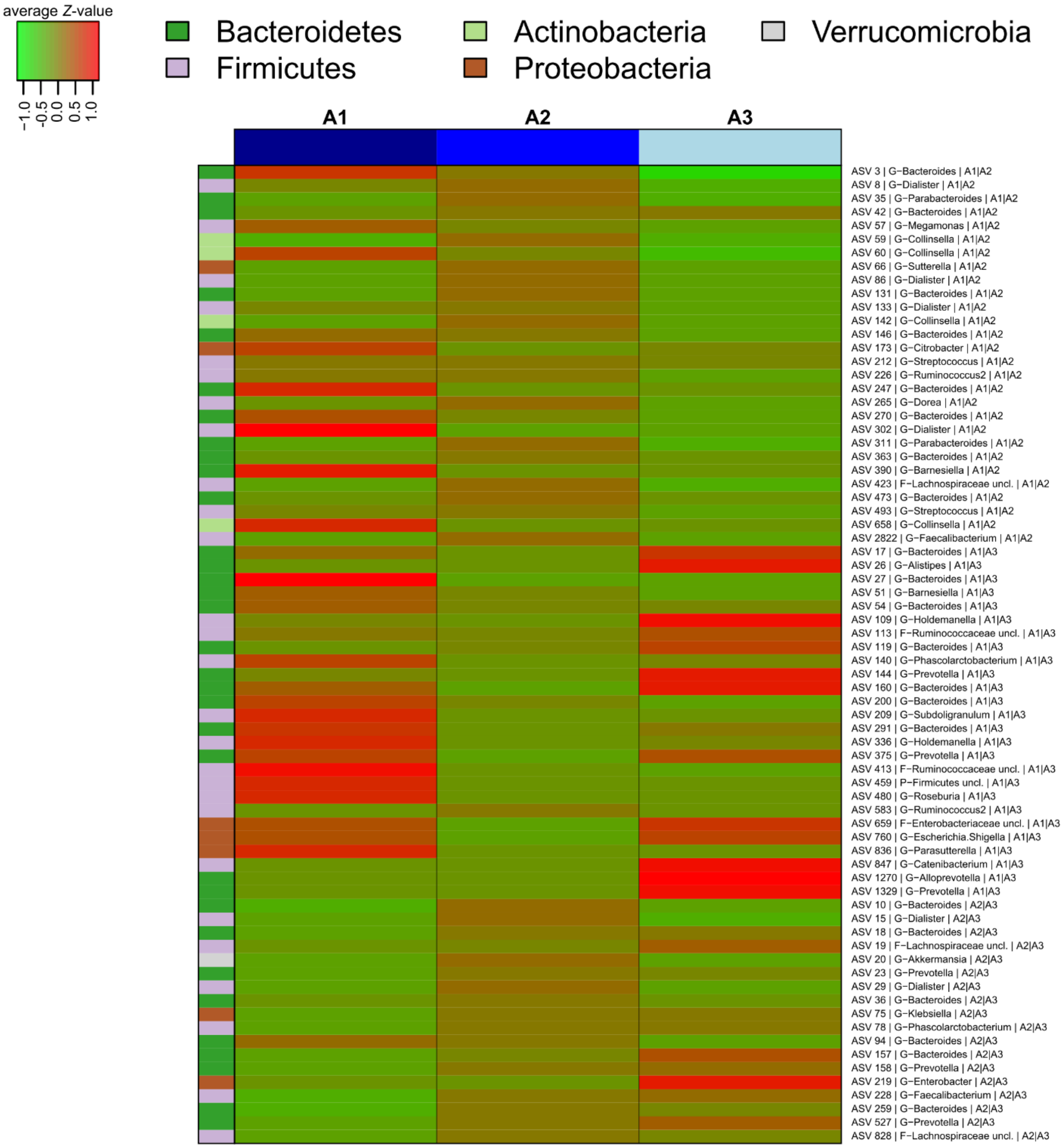

Appendix Figure 5: Heatmap visualizing significant differentially abundant ASVs in CD patients with respect to age subgroups following the Montreal classification (A1: <16 years at

diagnosis, A2: 17-40 years at diagnosis, A3: >40 years at diagnosis) (Appendix Table 8)

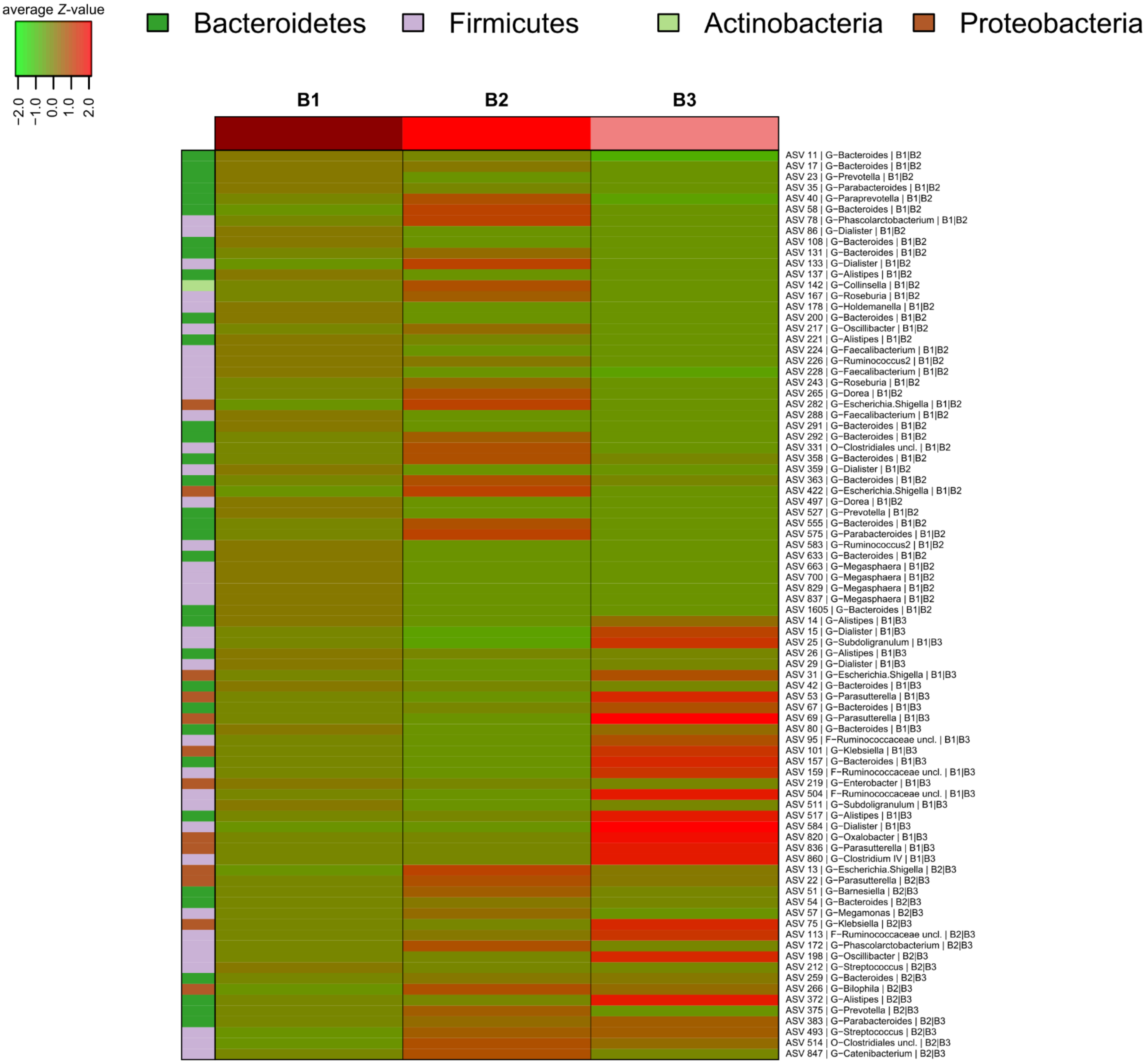

Appendix Figure 6: Heatmap visualizing significant differentially abundant ASVs in CD patients with respect to pathology/behaviour subgroups following the Montreal classification (B1: non-stricturing/non-penetrating, B2: stricturing, B3: penetrating). (Appendix Table 8)

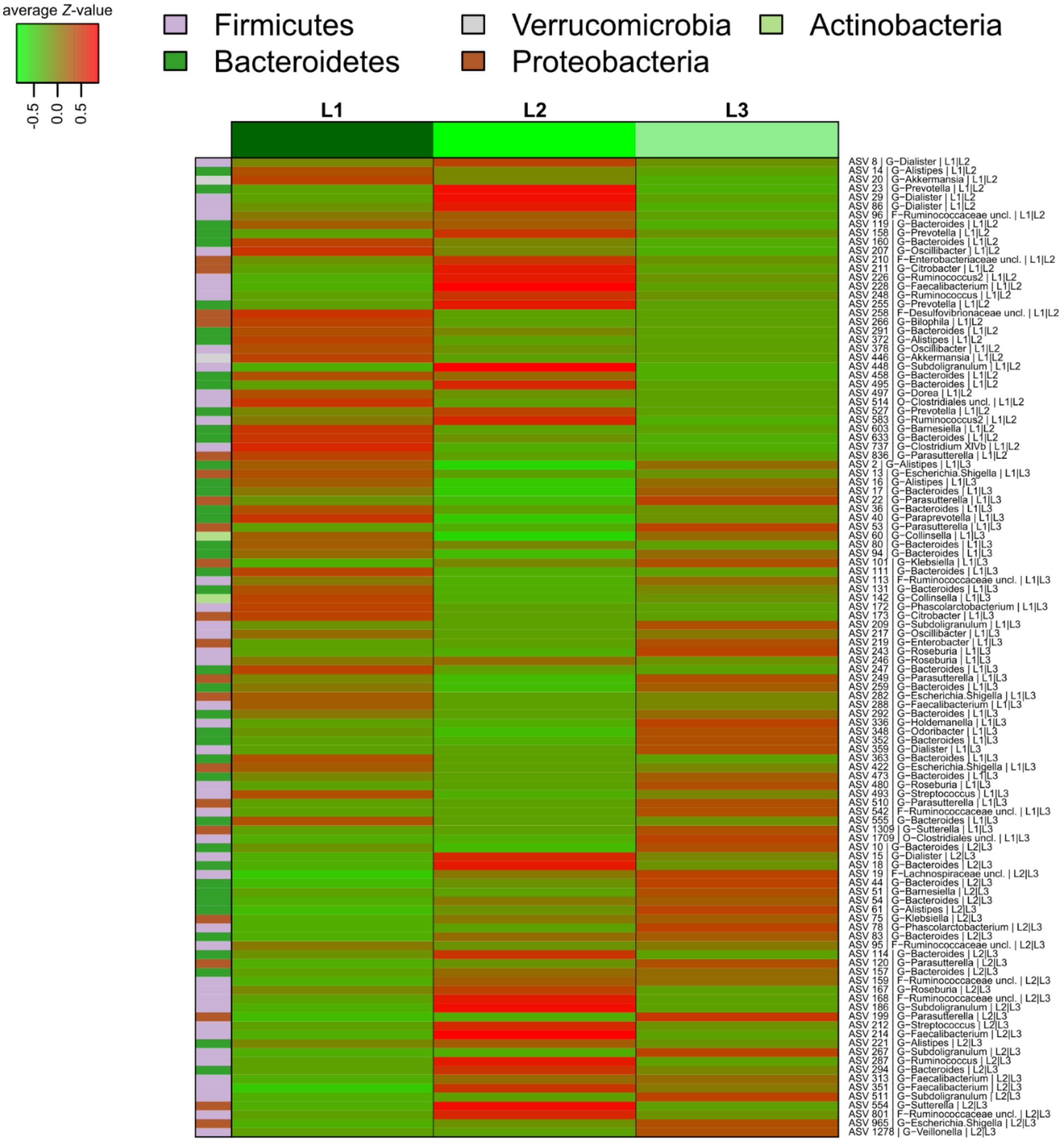

Appendix Figure 7: Heatmap visualizing significant differentially abundant ASVs in CD patients with respect to disease location subgroups following the Montreal classification (L1: ileal, L2: colonic, L3: ileocolonic) (Appendix Table 8).

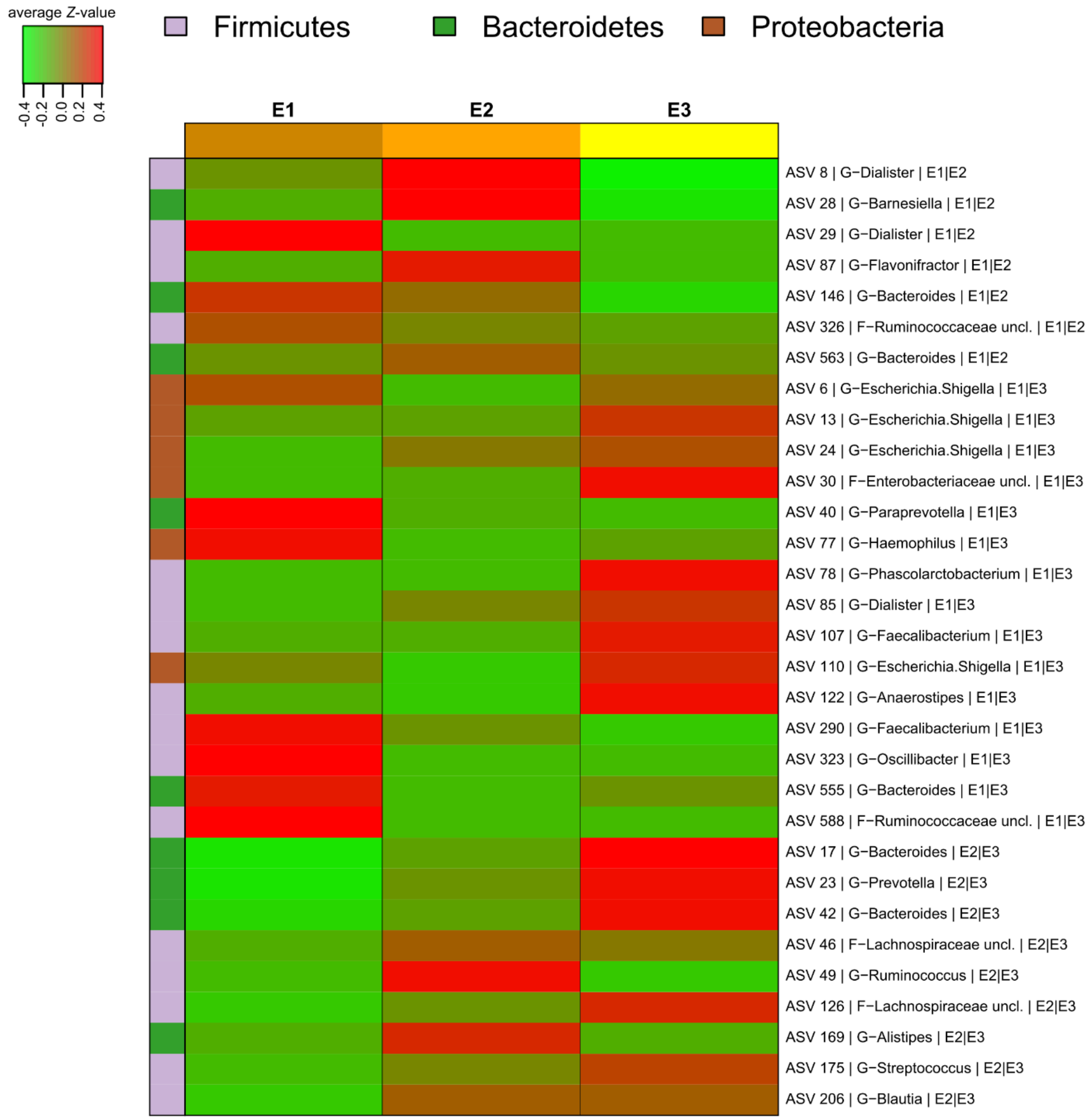

Appendix Figure 8: Heatmap visualizing significant differentially abundant ASVs in UC patients with respect to age subgroups following the Montreal classification. (E1: Ulcerative proctitis, E2: left sided UC (distal UC), E3: extensive UC (pancolitis) (Appendix Table 8).

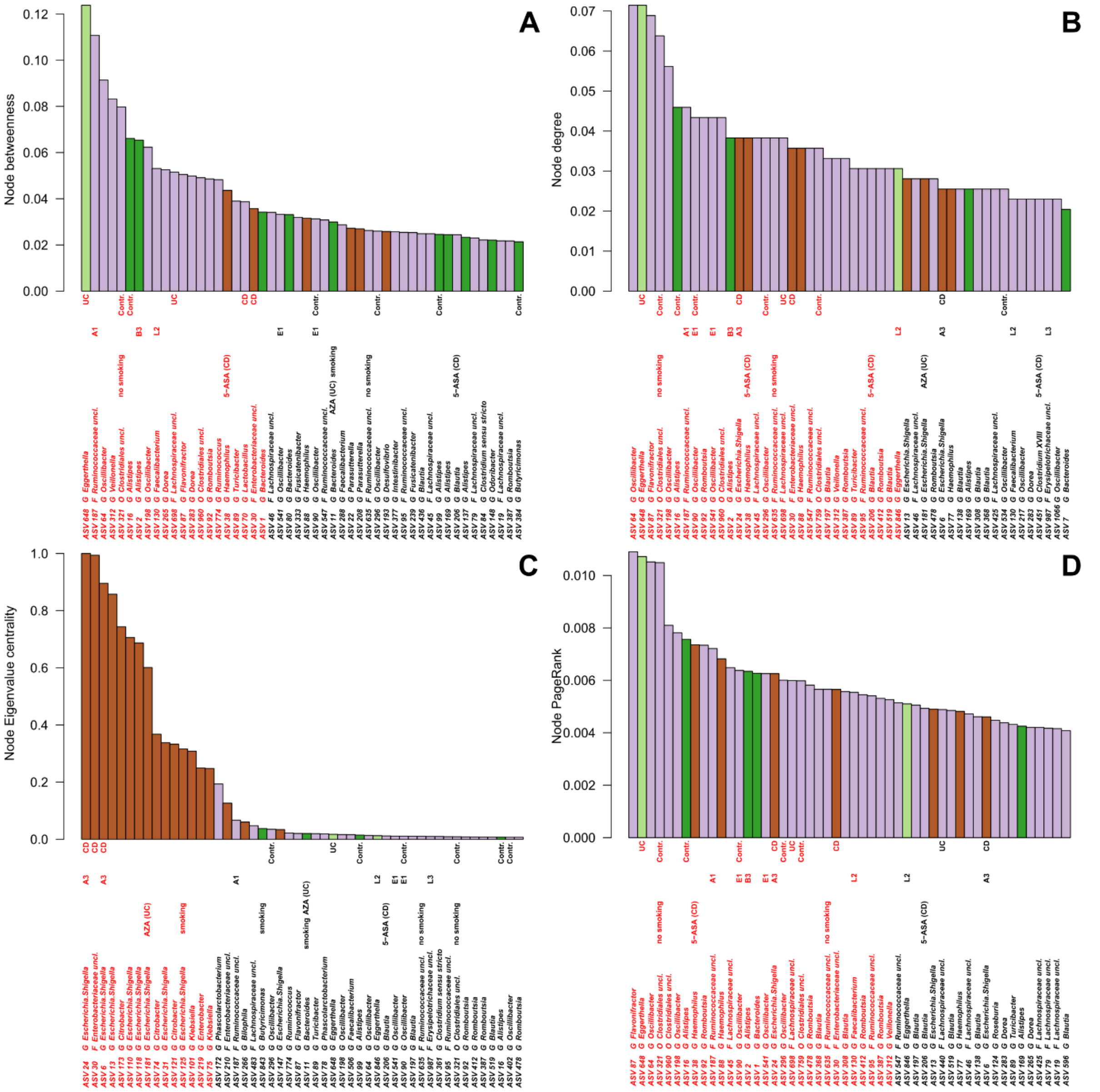

Appendix Figure 9: The barplots show the top 50 bacteria within the network based on their centrality measures (Betweenness, Degree, Eigenvector centrality, PageRank™).

Highlighted in red are network members with a higher importance than expected by chance, based on a Z-test against 10’000 randomized networks ($P \leq 0.05$). Highlighted are as well the associations of the respective ASVs to IBD status, medication, disease subtypes and smoking behaviour, as detected by indicator species analysis ($P \leq 0.05$, Appendix Table 11).

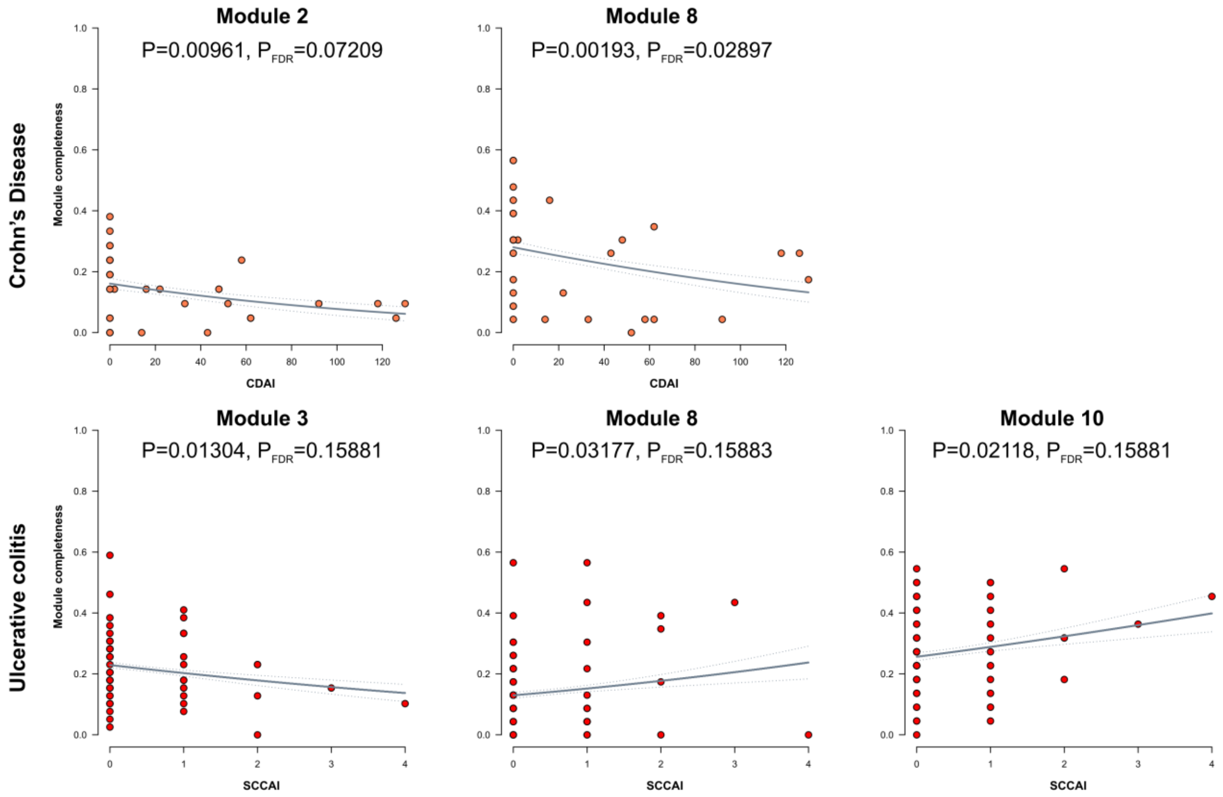

Appendix Figure 10: Correlation of module completeness with indicators of disease severity CDAI and SCCAI in CD patients and people suffering from UC, respectively.
